# Prevalence and Homology of the Pneumococcal Serine-Rich Repeat Protein at the Global Scale

**DOI:** 10.1128/spectrum.03252-22

**Published:** 2023-03-30

**Authors:** Javid Aceil, Aarya Venkat, Eric Pan, Natarajan Kannan, Fikri Y. Avci

**Affiliations:** a Department of Biochemistry and Molecular Biology, University of Georgia, Athens, Georgia, USA; b Department of Biochemistry, Emory Vaccine Center, Emory University School of Medicine, Atlanta, Georgia, USA; Institut Pasteur

**Keywords:** PsrP, Streptococcus pneumoniae, bioinformatics, epidemiology, protein vaccine

## Abstract

Pneumococcal pneumonia remains a WHO high-priority disease despite multivalent conjugate vaccines administered in clinical practice worldwide. A protein-based, serotype-independent vaccine has long-promised comprehensive coverage of most clinical isolates of the pneumococcus. Along with numerous pneumococcal surface protein immunogens, the pneumococcal serine-rich repeat protein (PsrP) has been investigated as a potential vaccine target due to its surface exposure and functions toward bacterial virulence and lung infection. Three critical criteria for its vaccine potential — the clinical prevalence, serotype distribution, and sequence homology of PsrP — have yet to be well characterized. Here, we used genomes of 13,454 clinically isolated pneumococci from the Global Pneumococcal Sequencing project to investigate PsrP presence among isolates, distribution among serotypes, and interrogate its homology as a protein across species. These isolates represent all age groups, countries worldwide, and types of pneumococcal infection. We found PsrP present in at least 50% of all isolates across all determined serotypes and nontypeable (NT) clinical isolates. Using a combination of peptide matching and HMM profiles built on full-length and individual PsrP domains, we identified novel variants that expand PsrP diversity and prevalence. We also observed sequence variability in its basic region (BR) between isolates and serotypes. PsrP has a strong vaccine potential due to its breadth of coverage, especially in nonvaccine serotypes (NVTs) when exploiting its regions of conservation in vaccine design.

**IMPORTANCE** An updated outlook on PsrP prevalence and serotype distribution sheds new light on the comprehensiveness of a PsrP-based protein vaccine. The protein is present in all vaccine serotypes and highly present in the next wave of potentially disease-causing serotypes not included in the current multivalent conjugate vaccines. Furthermore, PsrP is strongly correlated with clinical isolates harboring pneumococcal disease as opposed to pneumococcal carriage. PsrP is also highly present in strains and serotypes from Africa, where the need for a protein-based vaccine is the greatest, giving new reasoning to pursue PsrP as a protein vaccine.

## INTRODUCTION

The pneumococcal conjugate vaccines have been instrumental in reducing mortality; however, Streptococcus pneumoniae (Spn), as an infectious agent causing invasive pneumococcal disease (IPD), remains responsible for death in approximately 14% of all children under the age of 5 globally (1). These vaccines are overall effective for select serotypes. Still, with already over 100 serotypes in existence, a serotype-independent vaccine would serve as a line of defense against new serotypes on the rise, as well as bacterial evolutionary mechanisms such as serotype switching and antibiotic resistance (2). A conserved protein vaccine would further reduce pneumococcal disease incidence rates and be a crucial alternative for low-income countries exhibiting vastly different serotype distribution based on geography (3). Many Spn surface proteins have been evaluated in clinical trials. While some are still ongoing in phase II trials, it has been challenging to determine a conserved protein-based vaccine that can provide clear protection across a breadth of different serotypes (2).

Proteins such as pneumococcal histidine triad protein D (PhtD) and pneumococcal surface protein A (PspA) have shown promise as vaccine candidates with the potential ability to confer protection (4–8). While these proteins are present on the surface of almost all strains of the pneumococcus, it has recently been theorized that the capsular polysaccharide (CPS) of the pneumococcus can shield these surface proteins during invasive infection as an immune evasion mechanism (9, 10). A protein that can extend past the CPS may be a necessary consideration for a next-generation, protein-based pneumococcal vaccine.

Pneumococcal serine-rich repeat protein (PsrP) belongs to the Serine-rich Repeat Protein (SRRP) family commonly found in Gram-positive bacteria and previously characterized throughout the Streptococcus genus as well as S. aureus (11–13). These proteins are also heavily glycosylated (12). PsrP was first found in a transposon mutagenesis screening attempting to determine all virulence factors necessary for a lung infection in mice (14). When expressed as a recombinant protein with no posttranslational modifications (PTMs), PsrP has been linked to biofilm formation, lung adhesion, and lung infection in mice (15, 16). Importantly, PsrP extends past the capsule, which may avoid the possibility of capsular shielding of the host immune response during invasive infection (15). Recently, individual glycosyltransferases (GTs) that putatively act on PsrP were also implicated in the PsrP virulence (17). Thus, native, glycosylated PsrP has an important role in pneumococcal virulence. However, a clear understanding of its expression among clinical isolates and different serotypes and its potential for coverage on the global scale has been largely lacking.

PsrP function and virulence have been linked to its basic region (BR). The BR binds Keratin-10 on host lung cells (16) and has also been shown to bind extracellular DNA to promote biofilm formation (18). These characteristics, though well established, have been assessed using PsrP BR from specific strains, such as Spn TIGR4. Therefore, it is essential to determine PsrP virulence in other PsrP-containing clinical isolates and elucidate if PsrP basic region is conserved throughout pneumococcal isolates.

Here, we use an extensive database of 13,454 whole-genome clinical isolates compiled by the Global Pneumococcal Sequencing consortium to investigate the prevalence of PsrP in bacterial strains from hospitalized patients around the world. We find PsrP to be present in at least 50% of all strains, ranging up to over 70%, and it is heavily expressed in both vaccine and nonvaccine serotypes. We also observe PsrP homology as less conserved than previously thought, especially in the basic region.

## RESULTS

### Working data set, validation, and approach.

**FIG 1:**
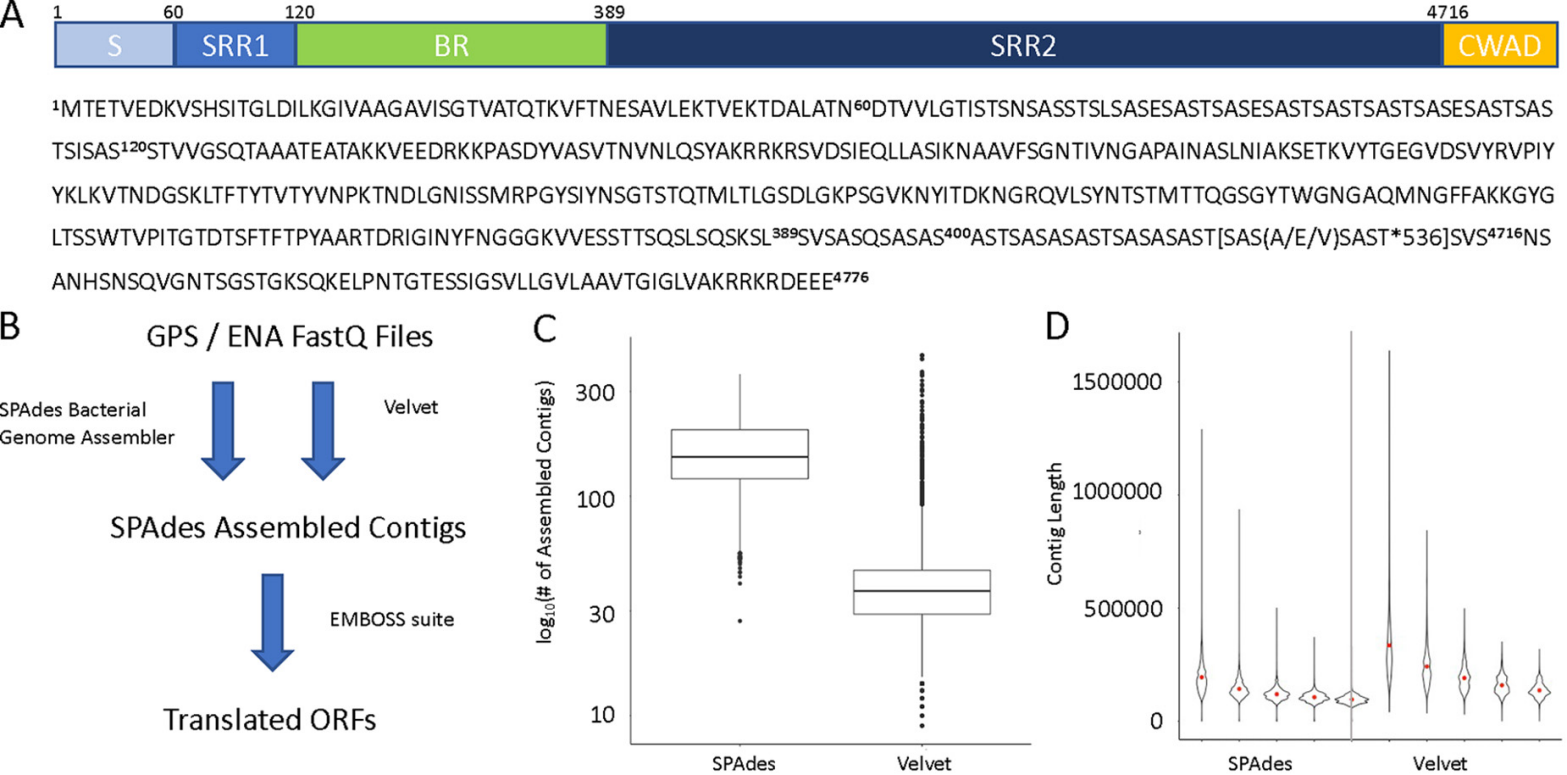
Bioinformatics design and workflow validation. (A) Schematic representation of PsrP by protein domains (signal peptide, S; serine-rich repeat region 1, SRR1; basic region, BR; serine-rich repeat region 2, SRR2; cell wall adhesion domain, CWAD) and protein sequence from the TIGR reference genome. Peptide 10-mers of interest are highlighted in yellow. (B) Workflow from raw sequence data to translated ORFs of each clinical isolate. (C and D) Comparison of SPAdes and Velvet assemblies by the number of contigs generated (C), and average contig length for each of the first five assembled contigs (D).

The pneumococcal serine-rich repeat protein comprises 4,776 amino acids and resides in a 37-kb locus in the pneumococcal genome (19). The protein can be subdivided into multiple domains: a signal peptide region (S), a serine-rich repeat region 1 (SRR1), a basic region (BR), a serine-rich repeat region 2 (SRR2), and a cell wall adhesion domain (CWAD) (Fig. 1A). The sequence of the protein comes from the reference sequence of the Spn TIGR4 strain (Fig. 1A) (20). Using this reference genome and the expected PsrP sequence, we interrogated a 13,454 pneumococcal clinical isolate database initially compiled by the Global Pneumococcal Sequencing project for the global assessment of geographical serotype distribution and antibiotic resistance (21). The workflow to generate translated ORFs of each assembled contig node involved pulling raw FASTQ files from the European Nucleotide Archive (ENA), using the bacterial genome assembler SPAdes, and creating protein translation of all files and contigs with EMBOSS (Fig. 1B). For comparison, we also obtained the Velvet assemblies from the original study (21). Each assembly was checked with the QUality ASsessment Tool (QUAST v5.0.2) (22). The number of contigs for each assembly (Fig. 1C) and contig length for each of the first five generated contigs in each assembly (Fig. 1D) show that the Velvet assemblies generated a smaller count of contigs that were larger on average. SPAdes, however, can outperform traditional assemblers like Velvet when working with bacterial genome assemblies (23). By using both a SPAdes and a Velvet assembly of the same whole-genome FASTQ reads, we aimed for increased rigor when reporting on the presence or absence of proteins in metagenomic analyses.

### Overall prevalence of PsrP at the global scale.

**FIG 2:**
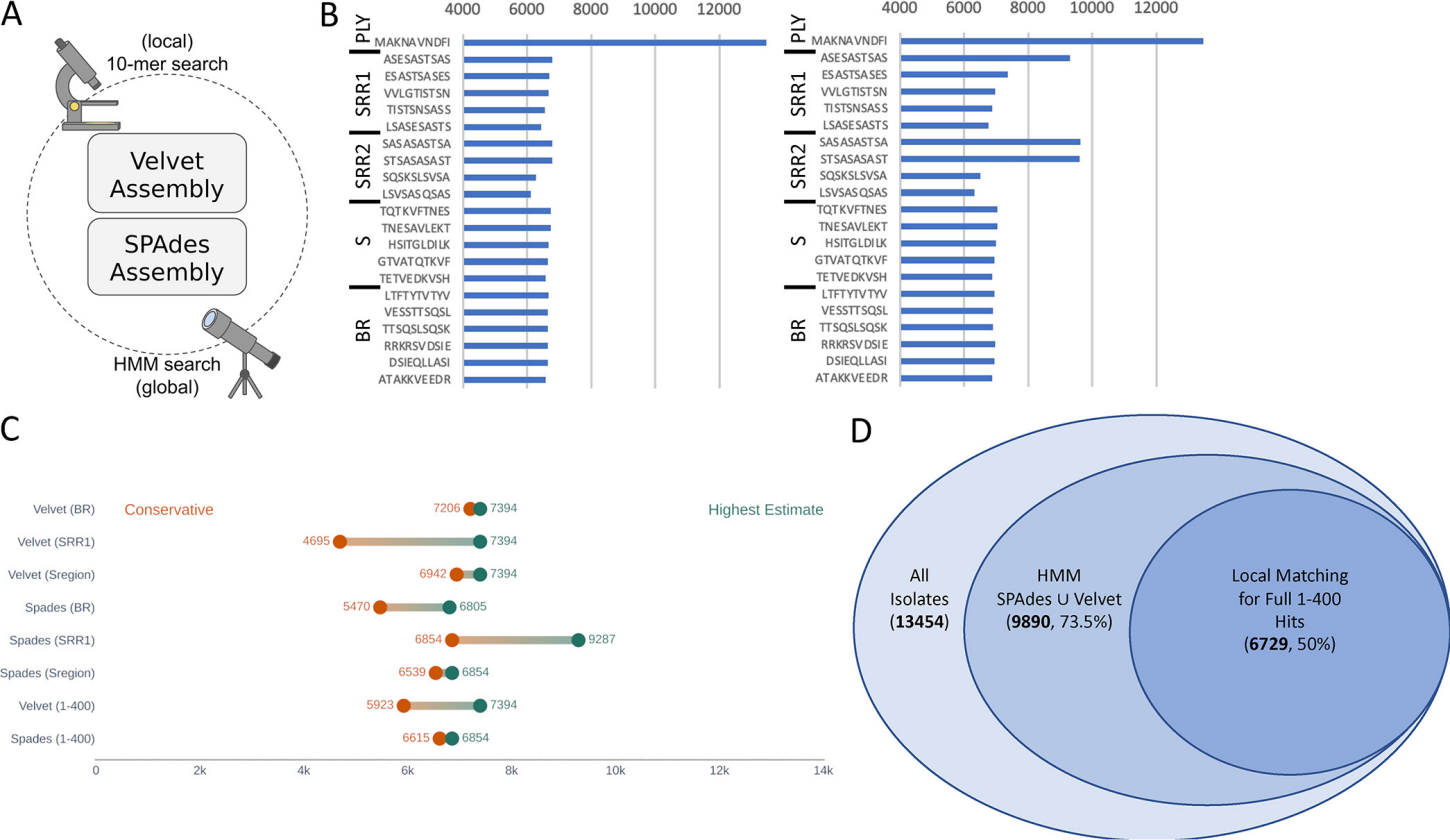
Local and global analysis of PsrP across both assemblies. (A) Cartoon of the methodology employed. (B) Select 10-mer peptides from each N-terminal region of PsrP (S, SRR1, BR, and SRR2) between assemblies. (C) Global analysis with trained HMM profiles against each region of PsrP and full-length (1 to 400) PsrP. (D) Venn diagram showcasing all isolates in the data set down to the final 6,729 hits.

To assess the presence of PsrP in our working data set, we utilized a local regex matching approach and a global HMM approach on both our SPAdes and Velvet assemblies (Fig. 2A). The protein sequence of the N-terminal regions of PsrP (AAs 1 to 412) was fragmented into all 403 potential 10 amino acid long peptides and iteratively searched for among all clinical isolate files (Table S1). Each 10-mer was BLASTed against the reference genome and shown to be unique to TIGR4 PsrP and no other proteins. Each 10-mer, as well as the entire N-terminal region of PsrP used for testing and the contigs in which PsrP were found, were also screened for bacteriophages using MetaPhinder and a previously published compilation of bacteriophage databases to add further confidence that false positives from potential phage assemblies were not involved (24). Multiple peptide matches covering each N-terminal region of PsrP were compared between the SPAdes and Velvet assemblies (Fig. 2B). For example, among the SPAdes assemblies, a minimum of 6,875 isolates were positive for PsrP by exact match when using the 10-mer peptide “TETVEDKVSH,” a portion of the signal peptide, while over 9,300 isolates were positive for PsrP when using the peptides “ASESASTSAS” and “ASTSASASAS” from the SRR1 and SRR2 regions, respectively. For Velvet assemblies, there were 6,567 matches for the S-region 10-mer peptide TETVEDKVSH, 6,786 matches for the SRR1-region 10-mer peptide ASESASTSAS, and 6,786 matches for the SRR2-region 10-mer peptide ASTSASASAS. The 10 amino acid peptide sequence “MANKAVNDFI” from pneumolysin (PLY), having 13,452 matches, was also used from the reference genome as a validation criterion for the working data set since PLY is expected to be present in all strains (25). Cumulatively, these positive isolates were cross-referenced between assemblies to show that a range of 6,000 to 9,600 isolates was positive for regex matching of the 10-mers used for screening and provided evidence that full-length N-terminal PsrP ranging from the S to the beginning of the SRR2 region can be recognized, validated, and extracted from the working data set for each clinical isolate that PsrP is present. Hidden Markov model (HMM) profiles were generated from isolates containing full-length N-terminal PsrP for both assemblies and used as an orthogonal approach to validate positive hits. Profiles for the whole N-terminal region (1 to 400), as well as profiles for each region of the N terminus (S, SRR1, and BR), were used to conduct a high-confidence rescan for sequence homology of all 13,454 isolates (Fig. 2C, Fig. S1). This resulted in a conservative estimate and a high estimate for each region where a conservative estimate represents near exact matches, and high estimate allows for more variations down to an E value confidence threshold of 1e-3. While no individual 10-mer peptide had over 7,000 hits in the Velvet assemblies, the trained HMM profiles for each region unanimously identified the potential for 7,394 hits, highlighting hundreds of new diverse sequences. In the SPAdes assemblies, the global approach was far more similar to the peptide approach with peptide matches from each region nearly identical to the global estimates.

**FIG 3:**
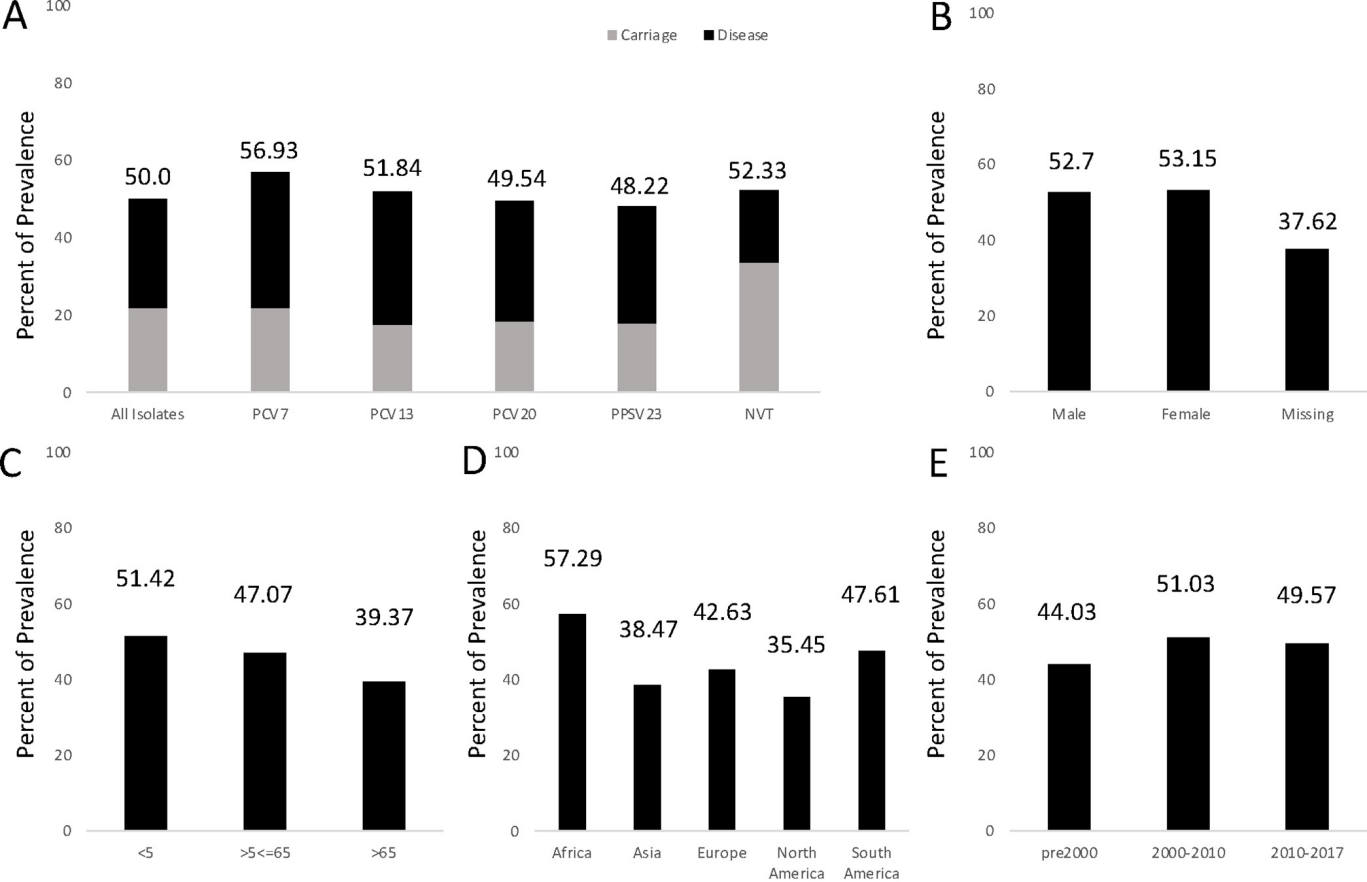
Conservative estimate of the overall prevalence of PsrP. (A) Percentage of PsrP-positive clinical isolates among all database isolates. PsrP-positive clinical isolates were also categorized by biological sex (B), patient age (C), geographical location (D), and time of infection (E).

When performing a union (∪) of the “PsrP-positive” isolates among Velvet and SPAdes assemblies by our global approach, unique positive isolates corresponded to each assembly resulting in a maximum of 9,890 PsrP-positive hits. However, to ensure a highly accurate count with tangible sequencing data of our target protein, we took the intersection (∩) of matching positive isolates from all four methods (SPAdes-HMM, Velvet-HMM, SPAdes-regex, Velvet-regex) to obtain a final working number of 6,729 PsrP-positive hits. Thus, PsrP is present in a minimum of 50% (*n* = 6,729/13,454) of all clinical and a potential maximum of 73.5% (*n* = 9,890/13,454) of all isolates when looking at 10-mers from the SRR1 and SRR2 regions (Fig. 2D and 3A).

To assess the landscape of PsrP-positive isolates, we utilized our conservative estimate from the intersection of all four analyses (*n* = 6,729) in relation to the known epidemiological data surrounding these patients. PsrP was present in ~50% of isolates in serotypes in multiple iterations of the pneumococcal conjugate vaccines. It is also important to note that the higher number of PsrP-positive isolates belong to patients hospitalized with the disease as opposed to carriage isolates (Fig. 3A). Beyond serotype data, we also assessed PsrP prevalence among biological sex, age, geographical location, and period of infection (Fig. 3B to E). While biological sex is not a distinguishing factor among isolates (Fig. 3B), a higher percentage of PsrP-containing isolates was observed in children under five than in other age groups (Fig. 3C). Geographically, Africa and South America had the highest rates of PsrP-containing isolates while Asia and North America had relatively lower PrsP prevalence (Fig. 3D). We also looked at the time of clinical infection among patients and observed similar percentages in PsrP-containing isolates despite the multidecade introductions of the conjugate vaccines. Thus, all metadata show similar trends in PsrP prevalence with a range from 35.45% up to 57.29%, depending on clinical factors and using the most conservative estimate. When using our highest potential estimate of 9,890 PsrP-positive hits (Fig. 2D), the range is escalated to being between 65.06% up to 80.38% (Fig. S2).

### PsrP in vaccine and nonvaccine serotypes.

**FIG 4:**
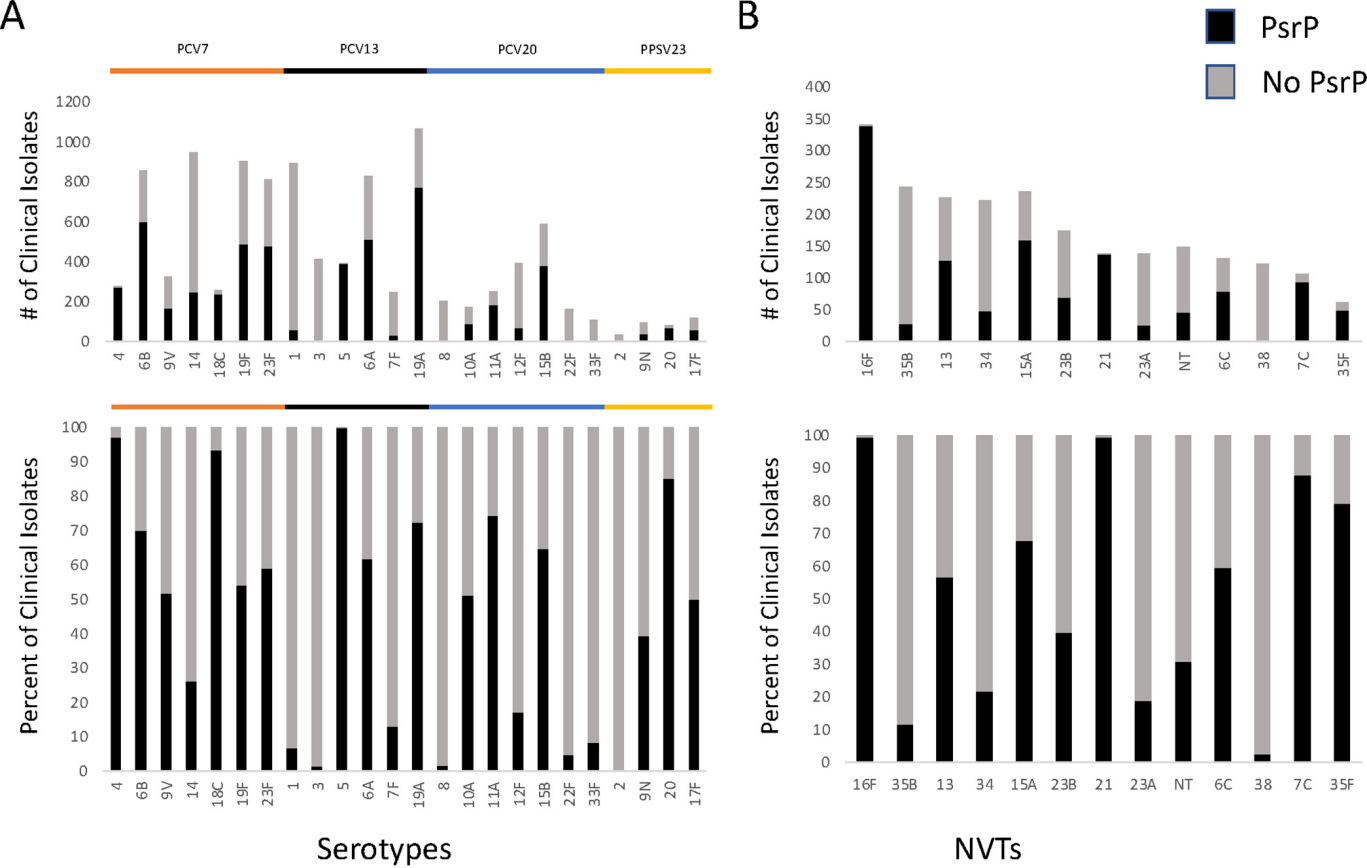
Conservative estimate of PsrP in vaccine and nonvaccine serotypes. (A) PsrP prevalence as the number of clinical isolates and the percentage for each serotype among the multiple conjugate vaccines on the market, and (B) in serotypes of decreasing incidence that are not included in the current conjugate vaccine formulations.

PsrP has had long-standing research as a potential vaccine candidate, although it is not present in all strains like some other protein immunogens (2). PsrP had previously been documented in TIGR4 and other serotype 4 strains and was predicted to be present in up to 50% of clinical isolates (26–28). Having observed PsrP as present in more disease-causing strains than previously reported, we assessed PsrP ubiquity with our conservative estimate among the high incidence rate serotypes included in the multivalent conjugate vaccines (29, 30). Serotype 4 strains showed a high volume of PsrP-positive isolates as expected, but interestingly, every serotype that is included in the PCV7 conjugate vaccine except serotype 14 had a greater than 50% abundance for PsrP in the bacterial genomes (Fig. 4A). This trend continued through PCV13 and PCV20 with only less than 50% PsrP presence in strains belonging to serotypes 1 (*n* = 59/893, 6.6%), 3 (*n* = 6/414, 1.45%), 8 (*n* = 3/203, 1.48%), 12F (*n* = 67/391, 17.14%), 22F (*n* = 8/165, 4.85%), and 33F (*n* = 9/107, 8.41%). While some of these serotypes are quite low, many of these serotypes have not been shown to carry this surface protein previously. When looking at serotypes not included in the vaccines, each has PsrP presence, with some close to 100% presence (Fig. 4B). Examining PsrP prevalence in serotypes with our highest estimate of 9,890 hits (Fig. 2D) substantiates our findings further (Fig. S3). It is likely that serotypes considered to be completely void of PsrP in fact have a nontraditional variant of the protein. For example, serotypes 3 (*n* = 181/414, 43.72%) or 8 (*n* = 55/203, 27.09%) show a significant difference by removing the peptide-matching stringency. Overall, these data showcase that PsrP covers important clinical serotypes and further implicate PsrP in bacterial isolates that cause disease.

### PsrP basic region is highly variable.

**FIG 5:**
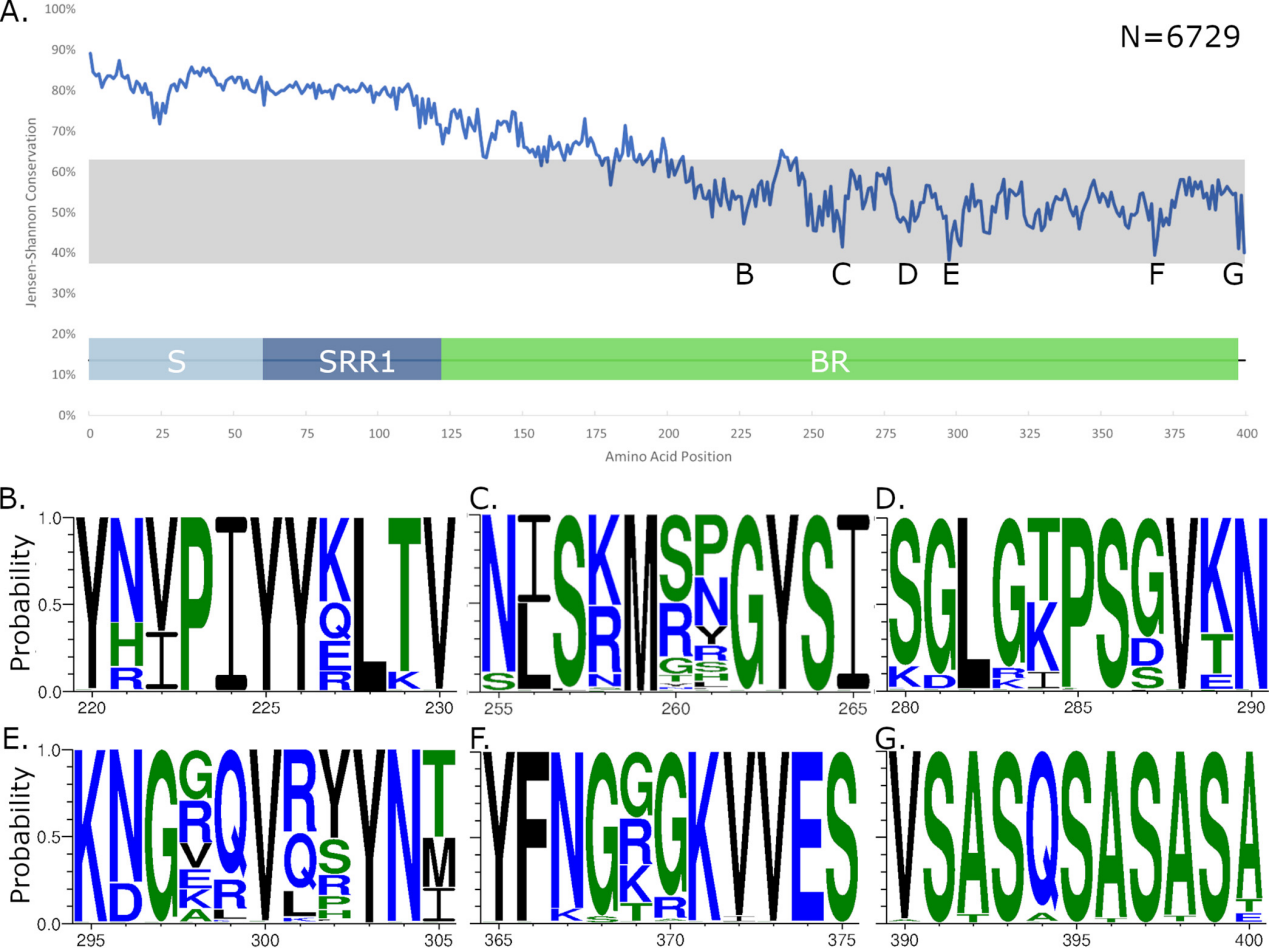
Homology of PsrP among clinical isolates. (A) Contig assemblies (*n* = 6,729) containing full-length N-terminal PsrP were extracted, and an alignment was created using MAFFT. Conservation of each residue from 1 to 400 of PsrP is shown. (B to G) Conservation of amino acid regions that drop below 60% is displayed as web logos of variation. The probability of potential amino acids is shown.

Outstanding previous research for PsrP as a vaccine candidate utilized the entire basic region to create a recombinantly expressed truncated version of PsrP (2, 15, 31). It was also observed that the basic region of PsrP interacts with Keratin-10 to adhere to the host lung cells (16, 32). PsrP has previously been thought to be a well-conserved protein, including all the associated GTs in the locus — either the entire locus was present or absent, and there was not any heterogeneity in the protein (12, 15, 28). To assess the state of conservation in PsrP among isolates, PsrP-positive bacterial genomes were selected based on the criterion that the first 400 AAs of the full-length N-terminal PsrP could be extracted from contig assemblies resulting in 6,729 “full” hits. These 6,729 PsrP sequences from clinical isolate raw sequence data were aligned using multiple sequence alignment based on fast Fourier transform (MAFFT), and sequence conservation was scored using Jensen-Shannon divergence (Fig. 5A) (33, 34). A web logo of the full 400 AA alignment also shows the sequence conservation through different regions of the protein (Fig. S4). Regions that drop below a threshold of 60% conservation are shown as web logos to visualize diversity in the PsrP sequence (Fig. 5B to G). The signal peptide and SRR1 regions have large conservation; however, the BR region, especially after amino acid 200, begins a clear region of high variability spanning the remaining BR region until the SRR2 region. This is important considering that this portion of the BR is where binding with Keratin-10 takes place. The data suggest that PsrP is more diverse as a protein than originally anticipated and should be strongly considered when attempting to \choose a conserved epitope of PsrP in vaccine design.

## DISCUSSION

When investigating a vaccine target, there must be a clear need and application for it among the infected population. While Spn continues to be a major threat to human health, the Spn protein-based vaccine design field has not looked favorably on PsrP for numerous reasons. It is a very large and heterogenous mucin-like protein that is difficult to isolate, purify, or fragment due to its highly glycosylated serine-rich regions. When surveying the pneumococcal protein vaccine field, there are hypothetically better options for Spn surface proteins to attempt a protein vaccine (2). However, few Spn proteins have the characteristics that PsrP does in terms of biofilm formation, lung cell adhesion, and lung infection. Furthermore, glycosylated repeat proteins have been implicated in pathogen evolution to invade more sensitive host tissues (35).

Our research used 13,454 whole-genome sequence isolates to investigate PsrP globally from the clinical setting. Contiguous sequences were generated from the raw sequence files and interrogated by peptide-matching and HMM approaches, as well as utilizing a previously published assembly to build confidence in the analysis. While a large portion of contigs contained full length PsrP, we are aware that the quality of assembly would impact PsrP counts, especially since PsrP contains repeat regions that could negatively impact the accuracy of contig assemblies. Future research will focus on more sophisticated genome assembly techniques for repeat region proteins as well as expand on the number of isolates.

An updated outlook on PsrP prevalence and serotype distribution sheds new light on the comprehensiveness of a PsrP-based protein vaccine. The protein is present in all vaccine serotypes and is also highly present in the next wave of potentially disease-causing serotypes not included in the current multivalent conjugate vaccines. Furthermore, the evidence highlights PsrP as strongly correlated with clinical isolates harboring pneumococcal disease as opposed to pneumococcal carriage, which is in concurrence with previous literature (26).

The finding of increased protein sequence variability in the BR is educating. Unglycosylated PsrP has been previously studied for protective capabilities as a vaccine, and while it conferred protection in mice for TIGR4, PsrP did not work well for other serotypes (31). The BR has also been implicated in biofilm formation and lung cell adhesion in Spn TIGR4. Our data showing high sequence diversity of the PsrP BR gives reason to postulate that these previous findings might be strain specific to TIGR4. The heterogeneity in the BR, especially from residues 200 to 375, showcases that a reevaluation of the protein holistically across serotypes and strains should be strongly considered. Furthermore, the discovery of highly mutated forms of the S or SRR1 region gives potential evidence of horizontal gene transfer among isolates and introduces a need for a better understanding of SRRP acquisition and utility. Because the GTs within the PsrP locus are putatively present unanimously when the protein is present, glycans associated with the function and virulence of the protein may represent a more conserved epitope for future vaccine formulations (17). Glycan portions of glycoconjugate vaccines and viral glycoprotein immunogens not only serve as the antigenic determinant but also contribute to the immunogenicity of these vaccines through eliciting T cell help (36–41). Thus, incorporating PsrP glycans in a glycoprotein vaccine may potentially enhance both the antigenic and immunogenic potential of PsrP and allow for a highly conserved vaccine target.

In conclusion, next-generation serotype-independent protein vaccine attempts have continued for over 4 decades without a consensus on the best targets to pursue. With advancements in feasibility and accessibility, whole-genome sequencing and analyses can be useful for knowledge-based vaccine design. Our findings serve to revitalize the potential of PsrP as a protein and/or glycoprotein vaccine target. Formulating a new PsrP protein vaccine in a region with high conservation or incorporating the glycan modifications to the protein as a glycoprotein immunogen may better serve the goal of a specific immune response that can provide broader protection against a multitude of IPD-causing serotypes.

## MATERIALS AND METHODS

### Data collection.

The Global Pneumococcal Sequencing (GPS) project's original database of 13,454 clinical isolates and associated metadata were used for this study, as previously described (21, 42). Illumina sequenced raw FASTQ files for each isolate were retrieved from the European Nucleotide Archive (ENA). Contig assembly and quality control with this data set were performed with the SPAdes bacterial genome assembler to create large contig FASTA files for each isolate (23). The complete list of accession numbers can be found in List S1 in the supplemental material. Of the 13,454 initial isolates, 13,417 remained after using SPAdes, as 37 isolates did not meet SPAdes confidence criteria. We compared this assembly against a previously published Velvet assembly (21). Qualitative analysis of each assembly was performed using QUality ASessment Tool v.5.0.2 (QUAST) (22). Each assembled nucleotide contig was translated into the six possible open reading frames (ORFs) using EMBOSS transeq to create the working data sets (43).

### Peptide match search.

The reference genome for the PsrP protein, also known as SP_1772, was the Streptococcus pneumoniae TIGR4, complete sequence (NCBI reference sequence NC_003028.3), spanning 4,776 amino acids in length (20). We truncated the amino acid sequence to the first 412 amino acids, ending the sequence after a few repeats of the SRR2 region of PsrP. We generated scripts using Perl and BioPerl (version 5.30 and 1.72, respectively) and performed regex matching using 10-mers of the PsrP sequence, resulting in 403 unique 10-mer peptides (Table S1). These 10-mers were used to search each translated ORF contig file for the presence of PsrP by amino acid sequence. We additionally scanned each ORF contig file with larger 20-mer peptides to validate our choice for using 10-mers.

### Sequence conservation analysis.

As mentioned before, the PsrP sequence was trimmed to 412 amino acids, from its N terminus through to the beginning of the SRR2 region. Due to the high repetitiveness of the SRR2 region, we delineate the first 400 amino acids as the primary PsrP domain. Using 6,729 “full” hits from our global HMM search, and local peptide matching approaches, a sequence alignment was generated using MAFFT v.7.490 (33). To analyze sequence conservation, we employed Jensen-Shannon divergence to score amino acid positions in the alignment (34). Domains were predicted using CDD-search (44), and AlphaFold2 (45), and the reference sequence was used in the context of the Jensen-Shannon score to analyze domain-level conservation. Sequence logos were produced from the alignment for regions below 60% conservation using WebLogo 3 (46).

### HMM search.

The sequence alignment from the 6,729 full hits from the SPAdes data set was also used to generate a hidden Markov model (HMM) profile using the HMMER3 suite (47). We further generated a sequence alignment using the Velvet data set and subsequently generated an HMM profile as a point of comparison. These profiles were used to further validate our hits by rescanning all possible ORFs of each file with HMM search. HMM searches allow for domain-level analyses, providing a global perspective as an orthogonal approach to our local peptide matching analysis. We further split our HMM profiles from the 400 amino acid PsrP domain to individual regions of the PsrP: the Signal peptide region (S), Serine Rich Repeat region 1 (SRR1), and the Basic region (BR). These were generated for both the SPAdes and Velvet data sets. This allowed us to assess the performance of the HMM search on the whole domain (400 amino acids) and each individual region of the PsrP domain. Performance was measured by counting the number of significant hits to the full domain or full region. Significant hits were categorized into two groups: a lower range of estimates (>1e-3) and an upper range of estimates (>1e-10). Hits that fell below the upper range of estimates were used to generate a “conservative estimate” of the hits by each HMM profile (Fig. 2C). Hits that fell below the lower range of estimates were included with the upper range to generate the “highest estimate” against each profile.

### Metadata analysis.

These data were compiled with metadata from the initial report (21) to associate serotype, age, sex, and other clinically relevant data for each isolate and evaluated using R statistical software. We additionally paired the hits against the contigs generated from the local peptide matching analysis with the global HMM search to identify the overlap of hits shared between the two methods, allowing for a more confident estimate of PsrP hits in each assembly. MetaPhinder was used to check each PsrP-positive contig for false positives against a bacteriophage database, as previously described (24).
