# Supplementary material for: Prevalence and Homology of the Pneumococcal Serine-Rich Repeat Protein at the Global Scale
Source: Microbiol Spectr. 2023 Mar 30;11(3):e03252-22. doi: 10.1128/spectrum.03252-22 (PMC10269691; doi:10.1128/spectrum.03252-22)
Supplement: Supplemental file 1 — Supplemental material. Download spectrum.03252-22-s0001.pdf, PDF file, 1.6 MB [file spectrum.03252-22-s0001.pdf]

Supplemental Figure S1. Histogram of HMM hits in SPAdes and Velvet assemblies with a match between residues 1-400 generated from HMMsearch. For ease of representation, expectation values (e-values) are converted as -log(e-value), where a number greater than three is considered significant.

Supplemental Figure S2. High Estimate of the Overall Prevalence of PsrP. (A) Percentage of PsrP-positive clinical isolates among all database isolates. PsrP-positive clinical isolates were also categorized by biological sex (B), patient age (C), geographical location (D), and time of infection (E).

Supplemental Figure S3. High Estimate of PsrP in Vaccine and Non-Vaccine Serotypes. (A) PsrP prevalence as the number of clinical isolates and the percentage for each serotype among the multiple conjugate vaccines on the market, and (B) in serotypes of decreasing incidence that are not included in the current conjugate vaccine formulations.

Supplemental Figure S4. Sequence Logo of Full Alignment of N-terminal PsrP. Contig assemblies (n = 6729) containing full-length N-terminal PsrP were extracted, and an alignment was created using MAFFT. The alignment was visualized as a sequence logo using WebLogo 3.0.

Figure S1

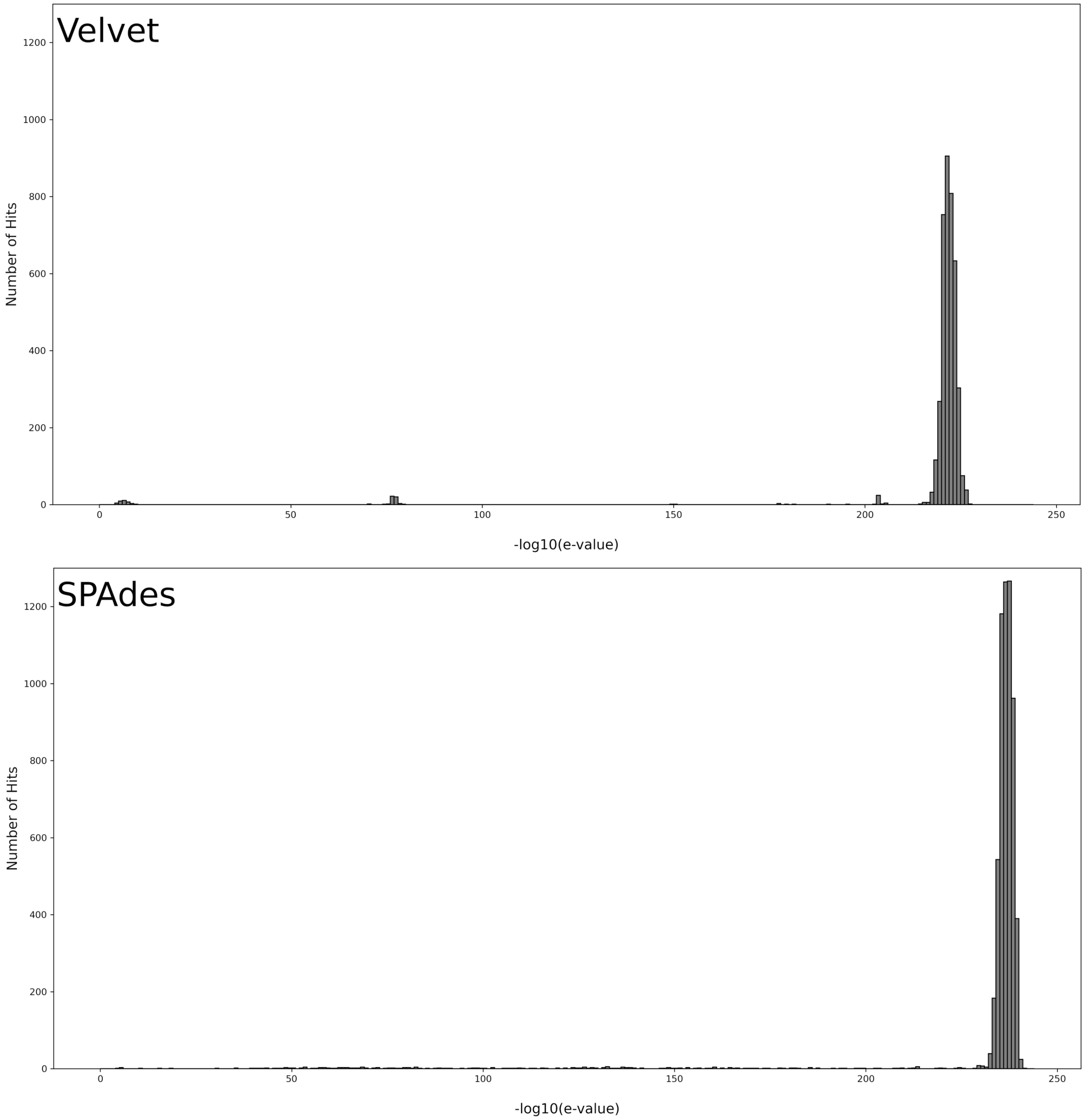
Velvet
Number of Hits
1200
1000
800
600
400
200
0
0
50
100
150
200
250
-log10(e-value)
SPAdes
Number of Hits
1200
1000
800
600
400
200
0
0
50
100
150
200
250
-log10(e-value)

Figure S2

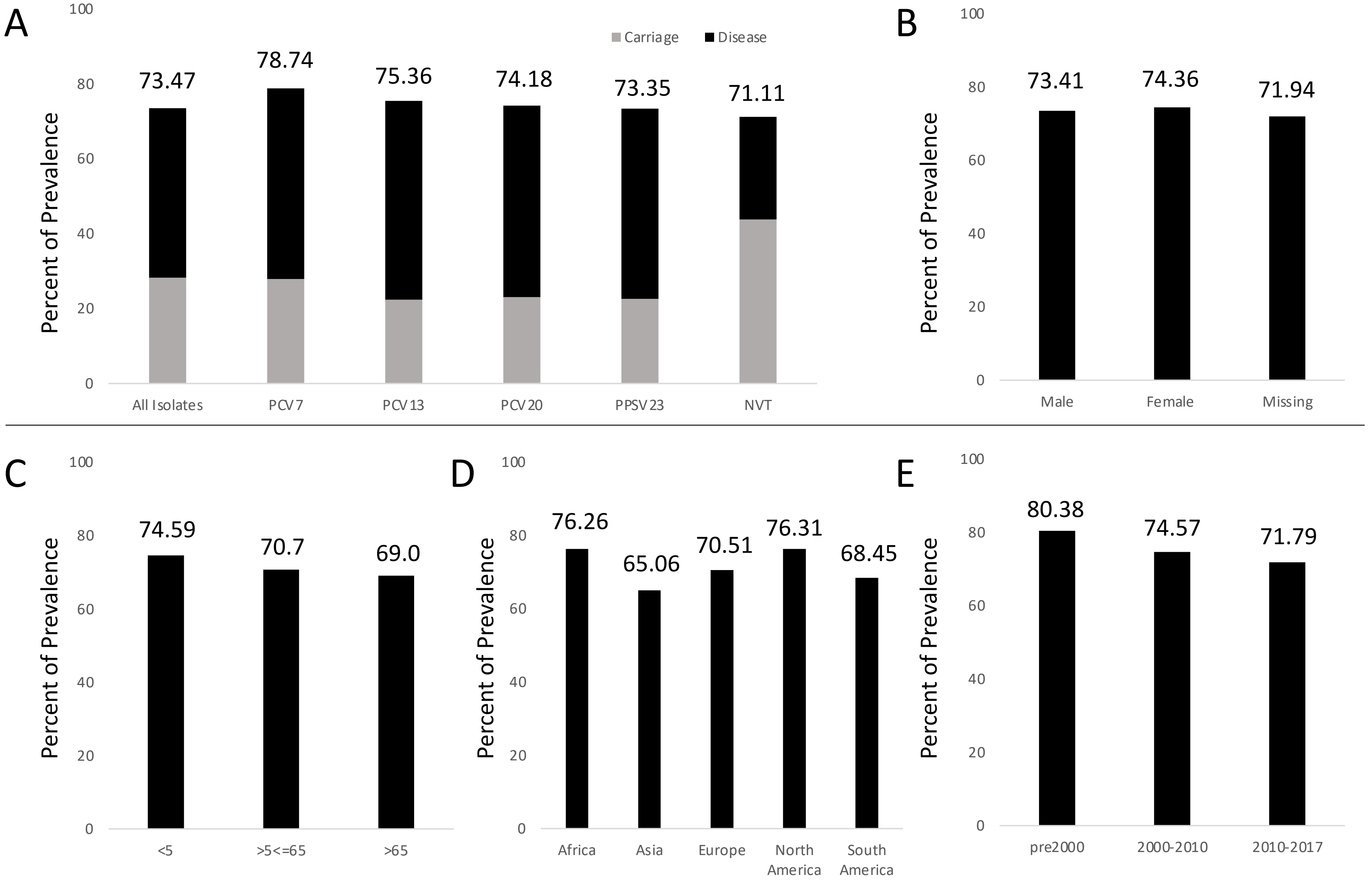

A
Carriage
Disease
Percent of Prevalence
100
80
60
40
20
0
73.47
78.74
75.36
74.18
73.35
71.11
All Isolates
PCV7
PCV13
PCV20
PPSV23
NVT
B
Percent of Prevalence
73.41
74.36
71.94
Male
Female
Missing
C
Percent of Prevalence
74.59
70.7
69.0
<5
>5<=65
>65
D
Percent of Prevalence
76.26
65.06
70.51
76.31
68.45
Africa
Asia
Europe
North America
South America
E
Percent of Prevalence
80.38
74.57
71.79
pre2000
2000-2010
2010-2017

Figure S3

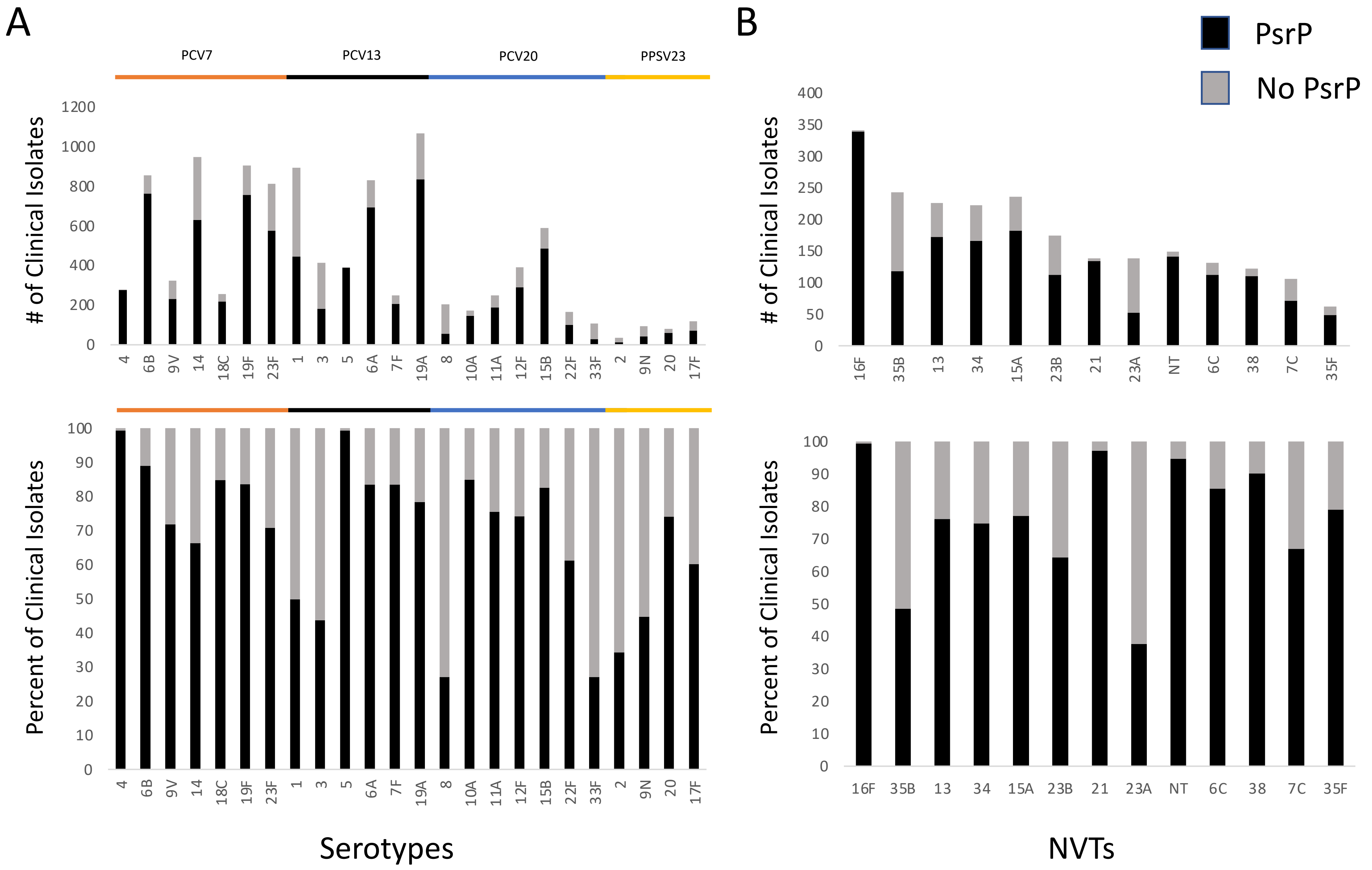

A
B
PCV7
PCV13
PCV20
PPSV23
PsrP
No PsrP
# of Clinical Isolates
Percent of Clinical Isolates
Serotypes
NVTs
4 6B 9V 14 18C 19F 23F 1 3 5 6A 7F 19A 8 10A 11A 12F 15B 22F 33F 2 9N 20 17F
16F 35B 13 34 15A 23B 21 23A NT 6C 38 7C 35F

Figure S4

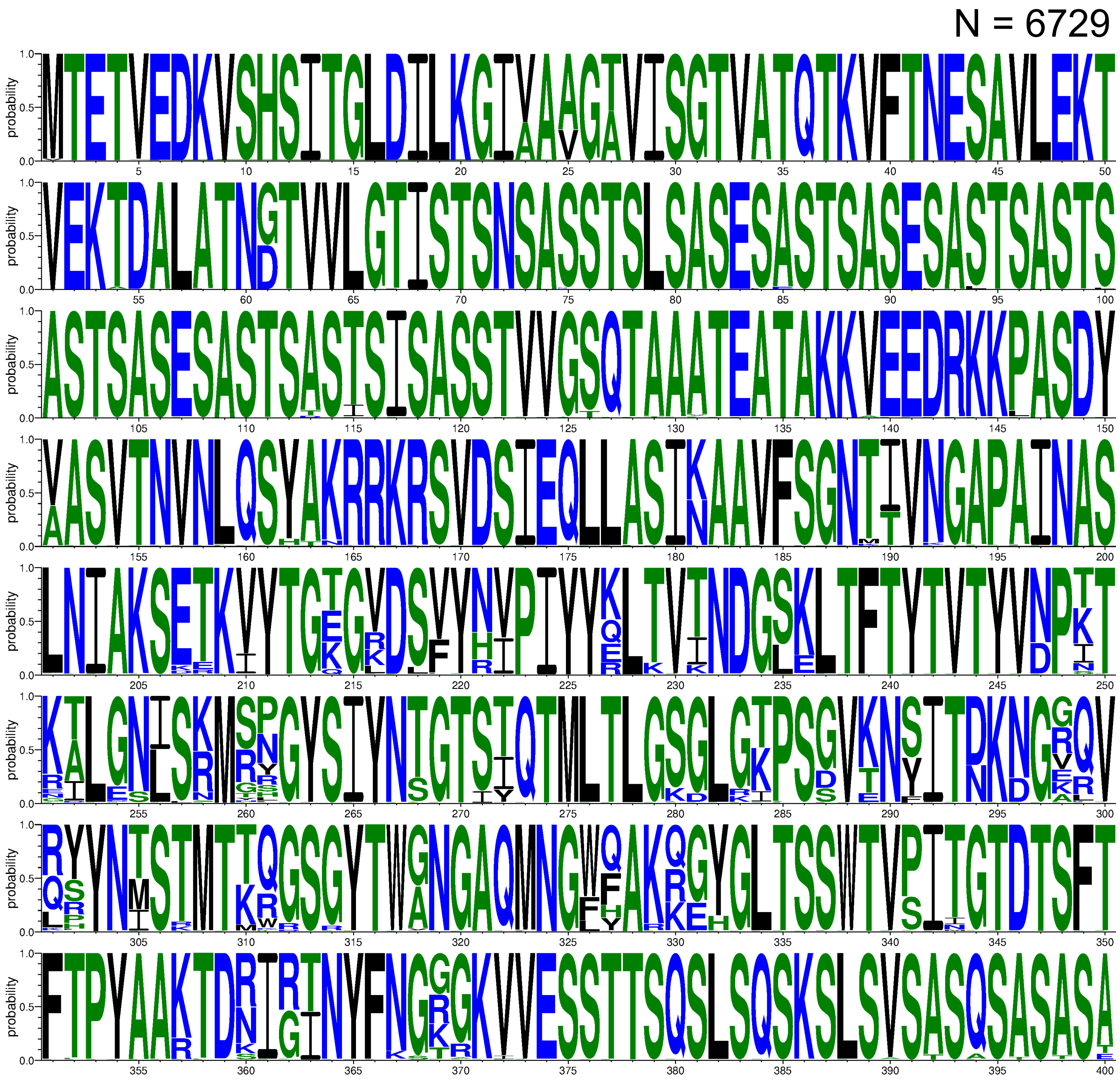

Supplemental Table 1. Complete list of peptides used and matches across all isolates for both assemblies.

SPAdes Assembly			Velvet Assembly		
NameOf10mer	Region	Hits	Name	Region	Hits
SASASASTSA	SRR2	9623	ASESASTSAS	SRR1	6786
ASASASTSAS	SRR2	9623	ASTSASASAS	SRR2	6786
SASTSASASA	SRR2	9622	SASASASTSA	SRR2	6786
ASTSASASAS	SRR2	9619	ASASASTSAS	SRR2	6786
STSASASAST	SRR2	9613	SASESASTSA	SRR1	6786
SASASTSASA	SRR2	9532	ASESASTSAS	SRR1	6786
ASASTSASAS	SRR2	9529	SASESASTSA	SRR1	6786
ASESASTSAS	SRR1	9308	SASTSASASA	SRR2	6786
ASESASTSAS	SRR1	9308	STSASASAST	SRR2	6785
SASESASTSA	SRR1	9306	TSASASASTS	SRR2	6785
SASESASTSA	SRR1	9306	ASTSASESAS	SRR1	6784
ASTSASESAS	SRR1	9243	STSASESAST	SRR1	6784
SASTSASESA	SRR1	9237	TSASESASTS	SRR1	6784
STSASESAST	SRR1	9231	SASTSASESA	SRR1	6783
TSASESASTS	SRR1	9225	SASASTSASA	SRR2	6778
ASTSASESAS	SRR1	9198	ASASTSASAS	SRR2	6778
SASTSASESA	SRR1	9195	ASTSASESAS	SRR1	6768
ASESASTSAS	SRR1	9166	SASTSASESA	SRR1	6767
SASESASTSA	SRR1	9165	STSASESAST	SRR1	6748
STSASESAST	SRR1	9086	TSASESASTS	SRR1	6748
TSASESASTS	SRR1	9085	SASESASTSA	SRR1	6745
ASTSASTSAS	SRR1	8262	ASESASTSAS	SRR1	6745
SASTSASTSA	SRR1	8258	TQTKVFTNES	S	6730
ASTSASTSAS	SRR1	8081	QTKVFTNESA	S	6730
SASTSASTSA	SRR1	8080	TKVFTNESAV	S	6730
STSASTSAST	SRR1	7992	KVFTNESAVL	S	6730
TSASTSASTS	SRR1	7985	VFTNESAVLE	S	6730
ESASTSASES	SRR1	7371	FTNESAVLEK	S	6730
SESASTSASE	SRR1	7365	TNESAVLEKT	S	6729
SESASTSAST	SRR1	7060	NESAVLEKTV	S	6728
TQTKVFTNES	S	7036	ESAVLEKTVE	S	6728
QTKVFTNESA	S	7036	SAVLEKTVEK	S	6728
TKVFTNESAV	S	7036	ASTSASTSAS	SRR1	6713
KVFTNESAVL	S	7036	DKVSHSITGL	S	6710
VFTNESAVLE	S	7036	ASTSASTSAS	SRR1	6705

FTNESAVLEK	S	7035	SASTSASTSA	SRR1	6704
TNESAVLEKT	S	7032	SASTSASTSA	SRR1	6690
NESAVLEKTV	S	7031	ESASTSASES	SRR1	6687
ESAVLEKTVE	S	7029	SESASTSASE	SRR1	6686
SAVLEKTVEK	S	7028	HSITGLDILK	S	6669
DKVSHSITGL	S	7020	SITGLDILKG	S	6669
ESASTSASTS	SRR1	7015	ITGLDILKGI	S	6669
ITGLDILKGI	S	6983	STSASTSAST	SRR1	6669
HSITGLDILK	S	6980	TSASTSASTS	SRR1	6669
SITGLDILKG	S	6979	VATQTKVFTN	S	6666
VLGTISTSNS	SRR1	6973	ATQTKVFTNE	S	6666
LGTISTSNSA	SRR1	6971	TVATQTKVFT	S	6665
VATQTKVFTN	S	6970	LTFTYTVTYV	BR	6661
SHSITGLDIL	S	6970	EDKVSHSITG	S	6659
VSHSITGLDI	S	6969	VSHSITGLDI	S	6659
TVATQTKVFT	S	6968	SHSITGLDIL	S	6659
VVLGTISTSN	SRR1	6968	VLGTISTSNS	SRR1	6658
ATQTKVFTNE	S	6968	LGTISTSNSA	SRR1	6658
RRKRSVDSIE	BR	6968	KVSHSITGLD	S	6658
EDKVSHSITG	S	6968	VVLGTISTSN	SRR1	6657
KVSHSITGLD	S	6968	VESSTTSQSL	BR	6653
RKRSVDSIEQ	BR	6965	ESSTTSQSLS	BR	6653
KRSVDSIEQL	BR	6962	SSTTSQSLSQ	BR	6653
RSVDSIEQLL	BR	6962	TVVLGTISTS	SRR1	6652
TVVLGTISTS	SRR1	6959	STTSQSLSQS	BR	6652
DSIEQLLASI	BR	6955	TTSQSLSQSK	BR	6649
VDSIEQLLAS	BR	6955	RRKRSVDSIE	BR	6647
SVDSIEQLLA	BR	6949	RKRSVDSIEQ	BR	6647
VISGTVATQT	S	6947	KRSVDSIEQL	BR	6647
SNSASSTSLS	SRR1	6945	RSVDSIEQLL	BR	6647
ISGTVATQTK	S	6945	GTVATQTKVF	S	6644
SGTVATQTKV	S	6945	SGTVATQTKV	S	6644
GTVATQTKVF	S	6944	VISGTVATQT	S	6643
STSNSASSTS	SRR1	6943	ISGTVATQTK	S	6643
ISTSNSASST	SRR1	6942	DSIEQLLASI	BR	6641
TSNSASSTSL	SRR1	6941	VDSIEQLLAS	BR	6641
NSASSTSLSA	SRR1	6939	SVDSIEQLLA	BR	6633
LTFTYTVTYV	BR	6939	SESASTSAST	SRR1	6629
ASVTNVNLQS	BR	6913	ISTSNSASST	SRR1	6626
INASLNIAKS	BR	6913	STSNSASSTS	SRR1	6626

SASSTSLSAS	SRR1	6904	TSNSASSTSL	SRR1	6625
ASSTSLSASE	SRR1	6899	SNSASSTSLS	SRR1	6624
ESSTTSQSLS	BR	6895	TSQSLSQSKS	BR	6621
VESSTTSQSL	BR	6893	SQSLSQSKSL	BR	6621
SSTTSQSLSQ	BR	6893	QSLSQSKSLS	BR	6619
STTSQSLSQS	BR	6892	NSASSTSLSA	SRR1	6619
TTSQSLSQSK	BR	6891	ASVTNVNLQS	BR	6604
TEATAKKVEE	BR	6886	INASLNIAKS	BR	6603
EATAKKVEED	BR	6886	DTSFTFTPYA	BR	6591
ATAKKVEEDR	BR	6886	TSFTFTPYAA	BR	6591
TAKKVEEDRK	BR	6879	TDTSFTFTPY	BR	6589
AKKVEEDRKK	BR	6879	SASSTSLSAS	SRR1	6583
ETVEDKVSHS	S	6878	ASSTSLSASE	SRR1	6581
VEDKVSHSIT	S	6878	ESASTSASTS	SRR1	6580
TETVEDKVSH	S	6875	TEATAKKVEE	BR	6578
TVEDKVSHSI	S	6875	EATAKKVEED	BR	6578
TISTSNSASS	SRR1	6867	ATAKKVEEDR	BR	6578
GTISTSNSAS	SRR1	6866	TAKKVEEDRK	BR	6574
PAINASLNIA	BR	6863	AKKVEEDRKK	BR	6574
AINASLNIAK	BR	6861	TETVEDKVSH	S	6567
TSQSLSQSKS	BR	6860	ETVEDKVSHS	S	6567
AVLEKTVEKT	S	6859	TVEDKVSHSI	S	6567
SISASSTVVG	SRR1	6859	VEDKVSHSIT	S	6567
SQSLSQSKSL	BR	6858	AVLEKTVEKT	S	6558
QSLSQSKSLS	BR	6857	GTDTSFTFTP	BR	6556
VLEKTVEKTD	S	6856	GTISTSNSAS	SRR1	6556
TDTSFTFTPY	BR	6852	TISTSNSASS	SRR1	6556
DTSFTFTPYA	BR	6852	VLEKTVEKTD	S	6555
TSFTFTPYAA	BR	6849	PAINASLNIA	BR	6554
EKTVEKTDAL	S	6844	AINASLNIAK	BR	6551
LEKTVEKTDA	S	6843	LEKTVEKTDA	S	6540
SSTSLSASES	SRR1	6822	EKTVEKTDAL	S	6540
GTDTSFTFTP	BR	6819	SLSQSKSLSV	BR	6516
GAPAINASLN	BR	6812	LSQSKSLSVS	BR	6514
APAINASLNI	BR	6812	SSTSLSASES	SRR1	6504
AAATEATAKK	BR	6798	GAPAINASLN	BR	6502
VEKTDALATN	S	6797	APAINASLNI	BR	6501
TAAATEATAK	BR	6797	KTVEKTDALA	S	6492
KTVEKTDALA	S	6797	VEKTDALATN	S	6491
QTAAATEATA	BR	6795	QTAAATEATA	BR	6491

TVEKTDALAT	S	6795	TAAATEATAK	BR	6491
SLSQSKSLSV	BR	6762	AAATEATAKK	BR	6491
LSQSKSLSVS	SRR2	6760	TVEKTDALAT	S	6491
SLSASESAST	SRR1	6756	STSLSASESA	SRR1	6436
TSLSASESAS	SRR1	6755	TSLSASESAS	SRR1	6436
STSLSASESA	SRR1	6754	SLSASESAST	SRR1	6435
STSASTSASE	SRR1	6754	LSASESASTS	SRR1	6434
LSASESASTS	SRR1	6754	MTETVEDKVS	S	6431
TSASTSASES	SRR1	6754	VVESSTTSQS	BR	6426
MTETVEDKVS	S	6738	ATEATAKKVE	BR	6407
ATEATAKKVE	BR	6710	KVVESSTTSQ	BR	6405
AATEATAKKV	BR	6705	AATEATAKKV	BR	6402
VNGAPAINAS	BR	6685	VNGAPAINAS	BR	6373
VVESSTTSQS	BR	6657	STSASTSASE	SRR1	6358
NGAPAINASL	BR	6646	TSASTSASES	SRR1	6349
KVVESSTTSQ	BR	6638	SISASSTVVG	SRR1	6343
KRRKRSVDSI	BR	6635	NGAPAINASL	BR	6333
EDRKKPASDY	BR	6584	KRRKRSVDSI	BR	6322
EEDRKKPASD	BR	6582	EDRKKPASDY	BR	6281
SQSKSLSVSA	SRR2	6506	EEDRKKPASD	BR	6281
QSKSLSVSAS	SRR2	6500	SQSKSLSVSA	SRR2	6270
ASSTVVGSQT	SRR1	6496	QSKSLSVSAS	SRR2	6264
SASSTVVGSQ	SRR1	6495	SKSLSVSASQ	SRR2	6259
SVTNVNLQSY	BR	6494	KSLSVSASQS	SRR2	6259
SKSLSVSASQ	SRR2	6494	SASSTVVGSQ	SRR1	6200
KSLSVSASQS	SRR2	6492	ASSTVVGSQT	SRR1	6200
KKVEEDRKKP	BR	6489	KKVEEDRKKP	BR	6194
KVEEDRKKPA	BR	6488	SVTNVNLQSY	BR	6193
VEEDRKKPAS	BR	6487	KVEEDRKKPA	BR	6193
ISASSTVVGS	SRR1	6394	VEEDRKKPAS	BR	6192
SSTVVGSQTA	SRR1	6393	SLSVSASQSA	SRR2	6109
STVVGSQTAA	BR	6388	LSVSASQSAS	SRR2	6109
NASLNIAKSE	BR	6374	SSTVVGSQTA	SRR1	6099
SLSVSASQSA	SRR2	6333	STVVGSQTAA	BR	6094
GSQTAAATEA	BR	6329	NASLNIAKSE	BR	6069
LSVSASQSAS	SRR2	6329	GSQTAAATEA	BR	6033
SQTAAATEAT	BR	6328	SQTAAATEAT	BR	6033
VVGSQTAAAT	BR	6327	VVGSQTAAAT	BR	6031
VGSQTAAATE	BR	6326	VGSQTAAATE	BR	6031
TVVGSQTAAA	BR	6320	TVVGSQTAAA	BR	6027

AKRRKRSVDS	BR	6313	AKRRKRSVDS	BR	6002
VTNVNLQSYA	BR	6174	ISASSTVVGS	SRR1	5885
VSASQSASAS	SRR2	6074	VTNVNLQSYA	BR	5873
SVSASQSASA	SRR2	6074	SVSASQSASA	SRR2	5850
ITGTDTSFTF	BR	6010	VSASQSASAS	SRR2	5849
SESASTSAST	SRR1	5963	ITGTDTSFTF	BR	5775
ESASTSASTS	SRR1	5943	TGTDTSFTFT	BR	5705
TGTDTSFTFT	BR	5938	SESASTSAST	SRR1	5629
SYAKRRKRSV	BR	5889	ESASTSASTS	SRR1	5603
YAKRRKRSVD	BR	5889	SYAKRRKRSV	BR	5591
QSYAKRRKRS	BR	5889	YAKRRKRSVD	BR	5591
NLQSYAKRRK	BR	5845	QSYAKRRKRS	BR	5591
NVNLQSYAKR	BR	5842	VNLQSYAKRR	BR	5550
VNLQSYAKRR	BR	5842	NLQSYAKRRK	BR	5550
TNVNLQSYAK	BR	5840	NVNLQSYAKR	BR	5549
LQSYAKRRKR	BR	5833	TNVNLQSYAK	BR	5548
KLTFTYTVTY	BR	5767	ASQSASASAS	SRR2	5545
SASQSASASA	SRR2	5753	SASQSASASA	SRR2	5543
ASQSASASAS	SRR2	5753	LQSYAKRRKR	BR	5539
QSASASASTS	SRR2	5724	QSASASASTS	SRR2	5524
SQSASASAST	SRR2	5719	SQSASASAST	SRR2	5517
ASLNIAKSET	BR	5662	KLTFTYTVTY	BR	5503
SLNIAKSETK	BR	5658	GKVVESSTTS	BR	5418
GKVVESSTTS	BR	5642	SLNIAKSETK	BR	5372
ASTSASTSIS	SRR1	5629	ASLNIAKSET	BR	5372
SASTSASTSI	SRR1	5612	ASTSASTSIS	SRR1	5265
TSISASSTVV	SRR1	5591	SASTSASTSI	SRR1	5248
STSISASSTV	SRR1	5507	TSISASSTVV	SRR1	5087
ASTSISASST	SRR1	5346	STSISASSTV	SRR1	5013
SASTSISASS	SRR1	5301	ASTSISASST	SRR1	4909
TSASTSISAS	SRR1	5204	SASTSISASS	SRR1	4893
STSASTSISA	SRR1	5183	TSASTSISAS	SRR1	4809
TFTYTVTYVN	BR	4862	STSASTSISA	SRR1	4808
FTYTVTYVNP	BR	4861	TFTYTVTYVN	BR	4714
IVNGAPAINA	BR	4784	FTYTVTYVNP	BR	4714
GLTSSWTVPI	BR	4692	IVNGAPAINA	BR	4509
DILKGIVAAG	S	4585	GLTSSWTVPI	BR	4461
LDILKGIVAA	S	4584	LDILKGIVAA	S	4322
GLDILKGIVA	S	4576	DILKGIVAAG	S	4322
TGLDILKGIV	S	4575	TGLDILKGIV	S	4316

AAVFSGNTIV	BR	4499	GLDILKGIVA	S	4316
FSGNTIVNGA	BR	4492	GYGLTSSWTV	BR	4264
SGNTIVNGAP	BR	4490	AAVFSGNTIV	BR	4229
AVFSGNTIVN	BR	4487	FSGNTIVNGA	BR	4225
VFSGNTIVNG	BR	4487	SGNTIVNGAP	BR	4225
GYGLTSSWTV	BR	4446	AVFSGNTIVN	BR	4220
GNTIVNGAPA	BR	4435	VFSGNTIVNG	BR	4220
NTIVNGAPAI	BR	4434	GNTIVNGAPA	BR	4173
TIVNGAPAIN	BR	4433	NTIVNGAPAI	BR	4173
ASDYVASVTN	BR	4380	TIVNGAPAIN	BR	4173
SDYVASVTNV	BR	4380	SDYVASVTNV	BR	4161
DYVASVTNVN	BR	4380	DYVASVTNVN	BR	4161
KPASDYVASV	BR	4377	ASDYVASVTN	BR	4160
PASDYVASVT	BR	4376	KPASDYVASV	BR	4156
DRKKPASDYV	BR	4372	PASDYVASVT	BR	4155
RKKPASDYVA	BR	4372	DRKKPASDYV	BR	4152
KKPASDYVAS	BR	4372	RKKPASDYVA	BR	4152
AKSETKVYTG	BR	4372	KKPASDYVAS	BR	4152
LNIAKSETKV	BR	4371	NIAKSETKVY	BR	4143
NIAKSETKVY	BR	4370	IAKSETKVYT	BR	4143
IAKSETKVYT	BR	4370	AKSETKVYTG	BR	4143
VASVTNVNLQ	BR	4319	LNIAKSETKV	BR	4142
YVASVTNVNL	BR	4319	VASVTNVNLQ	BR	4101
SIEQLLASIK	BR	4214	YVASVTNVNL	BR	4101
YTWGNGAQMN	BR	4080	SIEQLLASIK	BR	3985
TWGNGAQMNG	BR	4063	YTWGNGAQMN	BR	3859
WTVPITGTDT	BR	3941	TWGNGAQMNG	BR	3843
TVPITGTDTS	BR	3941	TSTQTMLTLG	BR	3810
VPITGTDTSF	BR	3938	GTSTQTMLTL	BR	3809
TSTQTMLTLG	BR	3933	VPITGTDTSF	BR	3738
PITGTDTSFT	BR	3933	WTVPITGTDT	BR	3738
GTSTQTMLTL	BR	3932	TVPITGTDTS	BR	3738
LTSSWTVPIT	BR	3921	PITGTDTSFT	BR	3736
TSSWTVPITG	BR	3885	LTSSWTVPIT	BR	3714
SWTVPITGTD	BR	3882	TSSWTVPITG	BR	3680
SSWTVPITGT	BR	3881	SSWTVPITGT	BR	3677
YGLTSSWTVP	BR	3857	SWTVPITGTD	BR	3677
SKLTFTYTVT	BR	3812	STQTMLTLGS	BR	3665

GSKLTFTYTV	BR	3809	YGLTSSWTVP	BR	3642
DGSKLTFTYT	BR	3804	GSKLTFTYTV	BR	3620
NDGSKLTFTY	BR	3789	SKLTFTYTVT	BR	3620
STQTMLTLGS	BR	3782	DGSKLTFTYT	BR	3615
SGYTWGNGAQ	BR	3699	NDGSKLTFTY	BR	3599
GYTWGNGAQM	BR	3696	SGYTWGNGAQ	BR	3531
GSGYTWGNGA	BR	3556	GYTWGNGAQM	BR	3527
MTTQGSGYTW	BR	3427	GSGYTWGNGA	BR	3393
STMTTQGSGY	BR	3127	MTTQGSGYTW	BR	3297
TMTTQGSGYT	BR	3127	GAVISGTVAT	S	3035
GAVISGTVAT	S	3119	AVISGTVATQ	S	3035
AVISGTVATQ	S	3117	STMTTQGSGY	BR	3003
DALATNDTVV	S	2882	TMTTQGSGYT	BR	3003
LATNDTVVLG	S	2881	DALATNDTVV	S	2754
ALATNDTVVL	S	2880	ALATNDTVVL	S	2754
DTVVLGTIST	SRR1	2823	LATNDTVVLG	S	2754
NDTVVLGTIS	S	2822	DTVVLGTIST	SRR1	2695
TNDTVVLGTI	S	2821	TNDTVVLGTI	S	2695
ATNDTVVLGT	S	2808	NDTVVLGTIS	S	2695
EKTDALATND	S	2784	ATNDTVVLGT	S	2684
KTDALATNDT	S	2784	EKTDALATND	S	2655
TDALATNDTV	S	2783	KTDALATNDT	S	2655
QGSGYTWGNG	BR	2638	TDALATNDTV	S	2655
TTQGSGYTWG	BR	2570	QGSGYTWGNG	BR	2516
TQGSGYTWGN	BR	2569	GGGKVVESST	BR	2466
GGGKVVESST	BR	2551	GGKVVESSTT	BR	2465
GGKVVESSTT	BR	2549	NYFNGGGKVV	BR	2460
NYFNGGGKVV	BR	2545	YFNGGGKVVE	BR	2460
NGGGKVVESS	BR	2543	FNGGGKVVES	BR	2460
YFNGGGKVVE	BR	2542	NGGGKVVESS	BR	2460
FNGGGKVVES	BR	2542	TQGSGYTWGN	BR	2449
VTNDGSKLTF	BR	2253	TTQGSGYTWG	BR	2449
TNDGSKLTFT	BR	2253	TNDGSKLTFT	BR	2110
KVYTGEGVDS	BR	1871	VTNDGSKLTF	BR	2109
TKVYTGEGVD	BR	1869	TKVYTGEGVD	BR	1800
NAAVFSGNTI	BR	1859	KVYTGEGVDS	BR	1798
YTGEGVDSVY	BR	1836	NAAVFSGNTI	BR	1767
VYTGEGVDSV	BR	1831	YTGEGVDSVY	BR	1764

YTVTYVNPKT	BR	1797	VYTGEGVDSV	BR	1759
TYTVTYVNPK	BR	1794	YTVTYVNPKT	BR	1734
GYSIYNSGTS	BR	1588	TYTVTYVNPK	BR	1731
KSETKVYTGE	BR	1564	GYSIYNSGTS	BR	1522
SETKVYTGEG	BR	1564	KSETKVYTGE	BR	1504
ETKVYTGEGV	BR	1560	SETKVYTGEG	BR	1504
GAQMNGFFAK	BR	1524	ETKVYTGEGV	BR	1498
NGAQMNGFFA	BR	1523	TDRIGINYFN	BR	1474
TDRIGINYFN	BR	1510	DRIGINYFNG	BR	1474
DRIGINYFNG	BR	1509	GAQMNGFFAK	BR	1436
INYFNGGGKV	BR	1404	NGAQMNGFFA	BR	1436
SFTFTPYAAR	BR	1362	INYFNGGGKV	BR	1361
FTFTPYAART	BR	1360	SFTFTPYAAR	BR	1321
TFTPYAARTD	BR	1344	FTFTPYAART	BR	1321
FTPYAARTDR	BR	1274	TFTPYAARTD	BR	1306
TPYAARTDRI	BR	1274	FTPYAARTDR	BR	1237
TSTMTTQGSG	BR	1252	TPYAARTDRI	BR	1237
NTSTMTTQGS	BR	1250	NSGTSTQTML	BR	1193
YNTSTMTTQG	BR	1247	SGTSTQTMLT	BR	1193
SGTSTQTMLT	BR	1244	IYNSGTSTQT	BR	1190
NSGTSTQTML	BR	1243	YNSGTSTQTM	BR	1190
YNSGTSTQTM	BR	1240	YSIYNSGTST	BR	1189
IYNSGTSTQT	BR	1239	SIYNSGTSTQ	BR	1189
YSIYNSGTST	BR	1237	TSTMTTQGSG	BR	1189
SIYNSGTSTQ	BR	1237	NTSTMTTQGS	BR	1187
AAGAVISGTV	S	1233	YNTSTMTTQG	BR	1184
AGAVISGTVA	S	1166	AAGAVISGTV	S	1179
SYNTSTMTTQ	BR	1055	AGAVISGTVA	S	1117
RIGINYFNGG	BR	1052	RIGINYFNGG	BR	1023
IGINYFNGGG	BR	1051	IGINYFNGGG	BR	1023
KGYGLTSSWT	BR	1050	GINYFNGGGK	BR	1022
GINYFNGGGK	BR	1050	SYNTSTMTTQ	BR	1001
AKKGYGLTSS	BR	1047	KGYGLTSSWT	BR	987
KKGYGLTSSW	BR	1047	AKKGYGLTSS	BR	984
PYAARTDRIG	BR	1000	KKGYGLTSSW	BR	984
YAARTDRIGI	BR	998	PYAARTDRIG	BR	969
AARTDRIGIN	BR	998	YAARTDRIGI	BR	967
ARTDRIGINY	BR	998	AARTDRIGIN	BR	967
RTDRIGINYF	BR	998	ARTDRIGINY	BR	967
VKNYITDKNG	BR	961	RTDRIGINYF	BR	967

WGNGAQMNGF	BR	950	VKNYITDKNG	BR	897
GNGAQMNGFF	BR	950	WGNGAQMNGF	BR	880
VLSYNTSTMT	BR	790	GNGAQMNGFF	BR	880
LSYNTSTMTT	BR	761	VLSYNTSTMT	BR	736
LKGIVAAGAV	S	761	GIVAAGAVIS	S	717
IVAAGAVISG	S	753	IVAAGAVISG	S	717
VAAGAVISGT	S	753	VAAGAVISGT	S	717
GIVAAGAVIS	S	752	ILKGIVAAGA	S	717
ILKGIVAAGA	S	752	LKGIVAAGAV	S	717
KGIVAAGAVI	S	752	KGIVAAGAVI	S	717
FFAKKGYGLT	BR	726	LSYNTSTMTT	BR	711
FAKKGYGLTS	BR	726	FFAKKGYGLT	BR	679
NGFFAKKGYG	BR	725	FAKKGYGLTS	BR	679
GFFAKKGYGL	BR	725	NGFFAKKGYG	BR	678
NYITDKNGRQ	BR	686	GFFAKKGYGL	BR	678
YITDKNGRQV	BR	686	NYITDKNGRQ	BR	642
MNGFFAKKGY	BR	681	YITDKNGRQV	BR	642
AQMNGFFAKK	BR	678	MNGFFAKKGY	BR	636
QMNGFFAKKG	BR	678	AQMNGFFAKK	BR	634
QVLSYNTSTM	BR	657	QMNGFFAKKG	BR	634
VYRVPIYYKL	BR	647	VYRVPIYYKL	BR	632
QTMLTLGSDL	BR	619	QVLSYNTSTM	BR	608
TMLTLGSDLG	BR	619	PGYSIYNSGT	BR	578
PGYSIYNSGT	BR	612	LGKPSGVKNY	BR	571
LGKPSGVKNY	BR	604	GKPSGVKNYI	BR	571
GKPSGVKNYI	BR	604	KPSGVKNYIT	BR	571
RPGYSIYNSG	BR	603	RPGYSIYNSG	BR	569
KPSGVKNYIT	BR	603	MRPGYSIYNS	BR	569
MRPGYSIYNS	BR	603	QTMLTLGSDL	BR	558
GVKNYITDKN	BR	581	TMLTLGSDLG	BR	558
PSGVKNYITD	BR	574	GVKNYITDKN	BR	543
SGVKNYITDK	BR	574	PSGVKNYITD	BR	538
DSVYRVPIYY	BR	440	SGVKNYITDK	BR	538
TGEGVDSVYR	BR	438	DSVYRVPIYY	BR	420
GVDSVYRVPI	BR	435	TGEGVDSVYR	BR	418
VDSVYRVPIY	BR	434	GVDSVYRVPI	BR	414
KNAAVFSGNT	BR	411	VDSVYRVPIY	BR	414
IEQLLASIKN	BR	411	KNAAVFSGNT	BR	384

EQLLASIKNA	BR	411	IEQLLASIKN	BR	384
IKNAAVFSGN	BR	411	EQLLASIKNA	BR	384
QLLASIKNAA	BR	410	QLLASIKNAA	BR	384
LLASIKNAAV	BR	410	LLASIKNAAV	BR	384
LASIKNAAVF	BR	410	LASIKNAAVF	BR	384
ASIKNAAVFS	BR	410	ASIKNAAVFS	BR	384
SIKNAAVFSG	BR	410	SIKNAAVFSG	BR	384
KNYITDKNGR	BR	409	IKNAAVFSGN	BR	384
GEGVDSVYRV	BR	337	KNYITDKNGR	BR	371
EGVDSVYRVP	BR	336	GEGVDSVYRV	BR	318
ITDKNGRQVL	BR	314	EGVDSVYRVP	BR	318
SVYRVPIYYK	BR	314	SVYRVPIYYK	BR	299
LTLGSDLGKP	BR	307	ITDKNGRQVL	BR	285
TLGSDLGKPS	BR	307	LKVTNDGSKL	BR	279
LGSDLGKPSG	BR	307	KVTNDGSKLT	BR	279
GSDLGKPSGV	BR	307	SDLGKPSGVK	BR	278
SDLGKPSGVK	BR	306	DLGKPSGVKN	BR	278
DLGKPSGVKN	BR	306	MLTLGSDLGK	BR	278
MLTLGSDLGK	BR	306	LTLGSDLGKP	BR	278
LKVTNDGSKL	BR	302	TLGSDLGKPS	BR	278
KVTNDGSKLT	BR	301	LGSDLGKPSG	BR	278
TDKNGRQVLS	BR	298	GSDLGKPSGV	BR	278
DKNGRQVLSY	BR	298	TVTYVNPKTN	BR	269
KNGRQVLSYN	BR	294	TDKNGRQVLS	BR	269
NGRQVLSYNT	BR	293	DKNGRQVLSY	BR	269
GRQVLSYNTS	BR	293	KNGRQVLSYN	BR	265
RQVLSYNTST	BR	293	NGRQVLSYNT	BR	265
TVTYVNPKTN	BR	289	GRQVLSYNTS	BR	265
TQTMLTLGSD	BR	238	RQVLSYNTST	BR	265
VPIYYKLKVT	BR	237	VPIYYKLKVT	BR	218
PIYYKLKVTN	BR	236	PIYYKLKVTN	BR	218
IYYKLKVTND	BR	236	IYYKLKVTND	BR	218
YYKLKVTNDG	BR	236	YYKLKVTNDG	BR	218
YKLKVTNDGS	BR	236	YKLKVTNDGS	BR	218
KLKVTNDGSK	BR	236	KLKVTNDGSK	BR	218
YRVPIYYKLK	BR	210	TQTMLTLGSD	BR	213
RVPIYYKLKV	BR	210	YRVPIYYKLK	BR	195
TNDLGNISSM	BR	139	RVPIYYKLKV	BR	195
NDLGNISSMR	BR	139	TNDLGNISSM	BR	119
DLGNISSMRP	BR	139	NDLGNISSMR	BR	119

LGNISSMRPG	BR	139	DLGNISSMRP	BR	119
GNISSMRPGY	BR	139	VTYVNPKTND	BR	119
NISSMRPGYS	BR	139	LGNISSMRPG	BR	119
KTNDLGNISS	BR	139	TYVNPKTNDL	BR	119
VNPKTNDLGN	BR	138	GNISSMRPGY	BR	119
ISSMRPGYSI	BR	138	YVNPKTNDLG	BR	119
NPKTNDLGNI	BR	138	NISSMRPGYS	BR	119
SSMRPGYSIY	BR	138	VNPKTNDLGN	BR	119
PKTNDLGNIS	BR	138	ISSMRPGYSI	BR	119
SMRPGYSIYN	BR	138	NPKTNDLGNI	BR	119
VTYVNPKTND	BR	137	SSMRPGYSIY	BR	119
TYVNPKTNDL	BR	137	PKTNDLGNIS	BR	119
YVNPKTNDLG	BR	137	SMRPGYSIYN	BR	119
