# Supplementary material for: Prevalence and Homology of the Pneumococcal Serine-Rich Repeat Protein at the Global Scale
Source: Microbiol Spectr. 2023 Mar 30;11(3):e03252-22. doi: 10.1128/spectrum.03252-22 (PMC10269691; doi:10.1128/spectrum.03252-22)
Supplement: Supplemental file 2 — List S1. Download spectrum.03252-22-s0002.pdf, PDF file, 0.3 MB [file spectrum.03252-22-s0002.pdf]

ERR352000
ERR352002
ERR352003
ERR352008
ERR352010
ERR352015
ERR352019
ERR351993
ERR352020
ERR352022
ERR352023
ERR352024
ERR352026
ERR352028
ERR352029
ERR352030
ERR352031
ERR352036
ERR352037
ERR351995
ERR352042
ERR352043
ERR352044
ERR352045
ERR352046
ERR351996
ERR352050
ERR352051
ERR352052
ERR352053
ERR352054
ERR351997
ERR351998
ERR351999
ERR387649
ERR425364
ERR425365
ERR425367
ERR425368
ERR425369
ERR425370
ERR425371
ERR425372
ERR425373
ERR425375
ERR425376
ERR425377
ERR425378
ERR425379
ERR425380

ERR425382
ERR425383
ERR425358
ERR425385
ERR425387
ERR425388
ERR425389
ERR425391
ERR425393
ERR425394
ERR425395
ERR425396
ERR425397
ERR425398
ERR425401
ERR425360
ERR425402
ERR425403
ERR425404
ERR425405
ERR425406
ERR425407
ERR425408
ERR425409
ERR425411
ERR425361
ERR425412
ERR425413
ERR425414
ERR425415
ERR425416
ERR425417
ERR425418
ERR425419
ERR425421
ERR425422
ERR425423
ERR425424
ERR425425
ERR425426
ERR425427
ERR425428
ERR425429
ERR425430
ERR425431
ERR425432
ERR425433
ERR425434
ERR425435
ERR425437

ERR425363
ERR425448
ERR425452
ERR425453
ERR425454
ERR425459
ERR425460
ERR425461
ERR425462
ERR425463
ERR425464
ERR425465
ERR425467
ERR425468
ERR425469
ERR425470
ERR425471
ERR425473
ERR425474
ERR425475
ERR425476
ERR425443
ERR425477
ERR425478
ERR425480
ERR425482
ERR425483
ERR425484
ERR425444
ERR425489
ERR425490
ERR425491
ERR425492
ERR425493
ERR425494
ERR425495
ERR425497
ERR425498
ERR425500
ERR425501
ERR425502
ERR425503
ERR425504
ERR425505
ERR425506
ERR425507
ERR425509
ERR425514
ERR425515
ERR425516

ERR425517
ERR425519
ERR425520
ERR425523
ERR425447
ERR425525
ERR425526
ERR425527
ERR425528
ERR425529
ERR433501
ERR433502
ERR433503
ERR433504
ERR433505
ERR433506
ERR433507
ERR433508
ERR433509
ERR433510
ERR433511
ERR433512
ERR433513
ERR433514
ERR433515
ERR433516
ERR433519
ERR433520
ERR433521
ERR433522
ERR433530
ERR433532
ERR433536
ERR433538
ERR433539
ERR433523
ERR433540
ERR433541
ERR433542
ERR433544
ERR433545
ERR433546
ERR433547
ERR433548
ERR433549
ERR433551
ERR433555
ERR433525
ERR433559
ERR433561

ERR433562
ERR433563
ERR433526
ERR433564
ERR433566
ERR433567
ERR433569
ERR433570
ERR433571
ERR433572
ERR433573
ERR433586
ERR433587
ERR433590
ERR433592
ERR433594
ERR433597
ERR433598
ERR433600
ERR433601
ERR433529
ERR433611
ERR433614
ERR433603
ERR433623
ERR433624
ERR433627
ERR433630
ERR433635
ERR433636
ERR433637
ERR433605
ERR433639
ERR433640
ERR433641
ERR433647
ERR433606
ERR433648
ERR433649
ERR433650
ERR433651
ERR433653
ERR433654
ERR433655
ERR433657
ERR433664
ERR433665
ERR433666
ERR433667
ERR433670

ERR433671
ERR433672
ERR433673
ERR433674
ERR433675
ERR433676
ERR433679
ERR433680
ERR433681
ERR433682
ERR433683
ERR433684
ERR433685
ERR433686
ERR433687
ERR433609
ERR433688
ERR433691
ERR433692
ERR433693
ERR433694
ERR433695
ERR433705
ERR433706
ERR433707
ERR433708
ERR433710
ERR433696
ERR433713
ERR433714
ERR433716
ERR433718
ERR433720
ERR433721
ERR433722
ERR433697
ERR433723
ERR433724
ERR433725
ERR433726
ERR433727
ERR433728
ERR433729
ERR433730
ERR433731
ERR433698
ERR433732
ERR433733
ERR433734
ERR433737

ERR433739
ERR433699
ERR433741
ERR433742
ERR433743
ERR433744
ERR433745
ERR433746
ERR433747
ERR433748
ERR433749
ERR433702
ERR433779
ERR433780
ERR433781
ERR433782
ERR433784
ERR433785
ERR433786
ERR433787
ERR433772
ERR433788
ERR433789
ERR433792
ERR433793
ERR433773
ERR433798
ERR433799
ERR433801
ERR433805
ERR433814
ERR433775
ERR433816
ERR433822
ERR433825
ERR433776
ERR433828
ERR433829
ERR433835
ERR433836
ERR433839
ERR433842
ERR433843
ERR433844
ERR433845
ERR433847
ERR433848
ERR433850
ERR433851
ERR433852

ERR433854
ERR433778
ERR433855
ERR433856
ERR433857
ERR433858
ERR433866
ERR433867
ERR433868
ERR433869
ERR433870
ERR433871
ERR433872
ERR433874
ERR433875
ERR433877
ERR433878
ERR433879
ERR433880
ERR433882
ERR433883
ERR433884
ERR433860
ERR433887
ERR433891
ERR433892
ERR433893
ERR433861
ERR433894
ERR433895
ERR433896
ERR433897
ERR433898
ERR433901
ERR433862
ERR433902
ERR433903
ERR433905
ERR433907
ERR433911
ERR433912
ERR433913
ERR433914
ERR433915
ERR433916
ERR433864
ERR433917
ERR433918
ERR433919
ERR433920

ERR433921
ERR433922
ERR433925
ERR433927
ERR433929
ERR433930
ERR433931
ERR433933
ERR433934
ERR433865
ERR433937
ERR433938
ERR433939
ERR433940
ERR433941
ERR433942
ERR433951
ERR433952
ERR433953
ERR433954
ERR433943
ERR433964
ERR433944
ERR433945
ERR433948
ERR433949
ERR433950
ERR433965
ERR433974
ERR433976
ERR433977
ERR433978
ERR433979
ERR433980
ERR433983
ERR433984
ERR433985
ERR433986
ERR433987
ERR433991
ERR433992
ERR433993
ERR433967
ERR433997
ERR434000
ERR434001
ERR434002
ERR434003
ERR434004
ERR434007

ERR434009
ERR433969
ERR434015
ERR434016
ERR434017
ERR434018
ERR434021
ERR434022
ERR434023
ERR434024
ERR434025
ERR434026
ERR434027
ERR434028
ERR434033
ERR433971
ERR434034
ERR434036
ERR434037
ERR434038
ERR434039
ERR434043
ERR434046
ERR434047
ERR434048
ERR434049
ERR434051
ERR434052
ERR434053
ERR434054
ERR434056
ERR434057
ERR434058
ERR434059
ERR434060
ERR438956
ERR438957
ERR438958
ERR438962
ERR438964
ERR438966
ERR438967
ERR438968
ERR438970
ERR438971
ERR438972
ERR438975
ERR438977
ERR438981
ERR438982

ERR438950
ERR438985
ERR438986
ERR438987
ERR438988
ERR438989
ERR438991
ERR438992
ERR438951
ERR438994
ERR438995
ERR438998
ERR438999
ERR439000
ERR439002
ERR438952
ERR439004
ERR438953
ERR449102
ERR449103
ERR449104
ERR449105
ERR449106
ERR449112
ERR449114
ERR449115
ERR449116
ERR449117
ERR449118
ERR449119
ERR449120
ERR449121
ERR449122
ERR449123
ERR449124
ERR449125
ERR449128
ERR449129
ERR449130
ERR449132
ERR449134
ERR449135
ERR449136
ERR449137
ERR449139
ERR449140
ERR449141
ERR449142
ERR449143
ERR449144

ERR449145
ERR449147
ERR449148
ERR449149
ERR449150
ERR449151
ERR449152
ERR449153
ERR449154
ERR449155
ERR449156
ERR449157
ERR449158
ERR449159
ERR449160
ERR449161
ERR449162
ERR449165
ERR449166
ERR449167
ERR449169
ERR449170
ERR449172
ERR449174
ERR449175
ERR449176
ERR449177
ERR449178
ERR449100
ERR449179
ERR449183
ERR449184
ERR449188
ERR449189
ERR449198
ERR449201
ERR449202
ERR449205
ERR449206
ERR449207
ERR449209
ERR449211
ERR449212
ERR449213
ERR449214
ERR449218
ERR449221
ERR449224
ERR449225
ERR449226

ERR449227
ERR449228
ERR449229
ERR449230
ERR449232
ERR449234
ERR449235
ERR449236
ERR449237
ERR449238
ERR449241
ERR449243
ERR449244
ERR449246
ERR449247
ERR449248
ERR449250
ERR449251
ERR449254
ERR449255
ERR449196
ERR449256
ERR449257
ERR449258
ERR449259
ERR449260
ERR449261
ERR449262
ERR449263
ERR449264
ERR449197
ERR460189
ERR460196
ERR460197
ERR460198
ERR460199
ERR460200
ERR460201
ERR460190
ERR460205
ERR460206
ERR460191
ERR460207
ERR460192
ERR460193
ERR460195
ERR467043
ERR467045
ERR467039
ERR490705

ERR490714
ERR490715
ERR490716
ERR490717
ERR490718
ERR490719
ERR490720
ERR490721
ERR490722
ERR490723
ERR490706
ERR490724
ERR490725
ERR490726
ERR490727
ERR490728
ERR490729
ERR490730
ERR490731
ERR490732
ERR490733
ERR490707
ERR490734
ERR490735
ERR490736
ERR490737
ERR490738
ERR490739
ERR490740
ERR490741
ERR490742
ERR490743
ERR490708
ERR490744
ERR490745
ERR490746
ERR490747
ERR490748
ERR490749
ERR490750
ERR490751
ERR490752
ERR490753
ERR490709
ERR490754
ERR490755
ERR490756
ERR490757
ERR490758
ERR490759

ERR490760
ERR490761
ERR490762
ERR490763
ERR490710
ERR490764
ERR490765
ERR490766
ERR490767
ERR490768
ERR490769
ERR490770
ERR490771
ERR490772
ERR490773
ERR490774
ERR490775
ERR490776
ERR490778
ERR490779
ERR490780
ERR490781
ERR490782
ERR490783
ERR490712
ERR490784
ERR490785
ERR490786
ERR490787
ERR490788
ERR490789
ERR490790
ERR490791
ERR490792
ERR490793
ERR490713
ERR490794
ERR490795
ERR490796
ERR490797
ERR490798
ERR490799
ERR501460
ERR501472
ERR501475
ERR501476
ERR501461
ERR501483
ERR501485
ERR501462

ERR501491
ERR501493
ERR501496
ERR501499
ERR501500
ERR501503
ERR501506
ERR501507
ERR501508
ERR501464
ERR501512
ERR501465
ERR501522
ERR501523
ERR501524
ERR501525
ERR501526
ERR501527
ERR501528
ERR501466
ERR501529
ERR501531
ERR501532
ERR501467
ERR501468
ERR501541
ERR501554
ERR501542
ERR501561
ERR501562
ERR501564
ERR501566
ERR501567
ERR501568
ERR501569
ERR501543
ERR501572
ERR501574
ERR501575
ERR501576
ERR501577
ERR501578
ERR501580
ERR501582
ERR501584
ERR501585
ERR501586
ERR501588
ERR501589
ERR501590

ERR501592
ERR501593
ERR501594
ERR501595
ERR501596
ERR501598
ERR501600
ERR501601
ERR501603
ERR501605
ERR501606
ERR501607
ERR501608
ERR501609
ERR501610
ERR501611
ERR501614
ERR501615
ERR501616
ERR501617
ERR501618
ERR501622
ERR501628
ERR501629
ERR501630
ERR505463
ERR505472
ERR505473
ERR505474
ERR505464
ERR505487
ERR505488
ERR505465
ERR505489
ERR505490
ERR505491
ERR505492
ERR505493
ERR505494
ERR505495
ERR505496
ERR505497
ERR505498
ERR505466
ERR505500
ERR505501
ERR505502
ERR505503
ERR505504
ERR505505

ERR505506
ERR505507
ERR505467
ERR505508
ERR505509
ERR505510
ERR505511
ERR505512
ERR505513
ERR505514
ERR505515
ERR505516
ERR505517
ERR505468
ERR505518
ERR505519
ERR505520
ERR505521
ERR505522
ERR505523
ERR505524
ERR505525
ERR505526
ERR505469
ERR505527
ERR505528
ERR505529
ERR505530
ERR505531
ERR505532
ERR505533
ERR505534
ERR505535
ERR505536
ERR505470
ERR505537
ERR505538
ERR505539
ERR505541
ERR505542
ERR505543
ERR505544
ERR505545
ERR505546
ERR505548
ERR505576
ERR505582
ERR505587
ERR505590
ERR505591

ERR505593
ERR505614
ERR505619
ERR505636
ERR505644
ERR505645
ERR505646
ERR505647
ERR505649
ERR505650
ERR505651
ERR505652
ERR505653
ERR505637
ERR505658
ERR505659
ERR505660
ERR505661
ERR505662
ERR505663
ERR505638
ERR505664
ERR505665
ERR505666
ERR505667
ERR505668
ERR505669
ERR505670
ERR505671
ERR505639
ERR505672
ERR505673
ERR505674
ERR505675
ERR505676
ERR505677
ERR505678
ERR505679
ERR505680
ERR505640
ERR505681
ERR505682
ERR505683
ERR505685
ERR505686
ERR505687
ERR505688
ERR505689
ERR505690
ERR505691

ERR505692
ERR505694
ERR505695
ERR505696
ERR505697
ERR505698
ERR505641
ERR505699
ERR505700
ERR505701
ERR505702
ERR505703
ERR505704
ERR505705
ERR505706
ERR505642
ERR505707
ERR505708
ERR505709
ERR505710
ERR505711
ERR505712
ERR505713
ERR505714
ERR505715
ERR505643
ERR505717
ERR505718
ERR505727
ERR505729
ERR505730
ERR505731
ERR505732
ERR505733
ERR505735
ERR505736
ERR505719
ERR505738
ERR505740
ERR505741
ERR505742
ERR505743
ERR505744
ERR505745
ERR505720
ERR505746
ERR505747
ERR505748
ERR505749
ERR505750

ERR505751
ERR505753
ERR505721
ERR505755
ERR505756
ERR505758
ERR505760
ERR505761
ERR505762
ERR505763
ERR505765
ERR505766
ERR505767
ERR505768
ERR505769
ERR505770
ERR505771
ERR505772
ERR505723
ERR505773
ERR505774
ERR505776
ERR505777
ERR505778
ERR505779
ERR505782
ERR505724
ERR505786
ERR505788
ERR505789
ERR505790
ERR505791
ERR505792
ERR505793
ERR505794
ERR505795
ERR505796
ERR505797
ERR505799
ERR505800
ERR505801
ERR505726
ERR505803
ERR505813
ERR505818
ERR505819
ERR505821
ERR505804
ERR505826
ERR505827

ERR505829
ERR505830
ERR505831
ERR505805
ERR505835
ERR505836
ERR505837
ERR505840
ERR505841
ERR505842
ERR505843
ERR505844
ERR505846
ERR505847
ERR505848
ERR505849
ERR505850
ERR505807
ERR505853
ERR505854
ERR505855
ERR505856
ERR505857
ERR505858
ERR505859
ERR505860
ERR505861
ERR505808
ERR505863
ERR505864
ERR505866
ERR505867
ERR505868
ERR505870
ERR505871
ERR505872
ERR505873
ERR505874
ERR505875
ERR505876
ERR505881
ERR505882
ERR505883
ERR505884
ERR505885
ERR505886
ERR505887
ERR505888
ERR505889
ERR505890

ERR505811
ERR505892
ERR505901
ERR505902
ERR505903
ERR505904
ERR505905
ERR505907
ERR505908
ERR505909
ERR505910
ERR505893
ERR505911
ERR505912
ERR505913
ERR505914
ERR505915
ERR505916
ERR505917
ERR505918
ERR505919
ERR505920
ERR505894
ERR505921
ERR505922
ERR505923
ERR505924
ERR505925
ERR505926
ERR505927
ERR505928
ERR505929
ERR505930
ERR505895
ERR505931
ERR505932
ERR505933
ERR505934
ERR505935
ERR505937
ERR505938
ERR505939
ERR505940
ERR505896
ERR505941
ERR505942
ERR505943
ERR505944
ERR505945
ERR505946

ERR505947
ERR505948
ERR505949
ERR505950
ERR505897
ERR505951
ERR505952
ERR505953
ERR505954
ERR505955
ERR505956
ERR505957
ERR505958
ERR505959
ERR505960
ERR505898
ERR505961
ERR505962
ERR505963
ERR505964
ERR505965
ERR505966
ERR505967
ERR505968
ERR505969
ERR505970
ERR505899
ERR505971
ERR505972
ERR505973
ERR505974
ERR505975
ERR505976
ERR505977
ERR505979
ERR505980
ERR505900
ERR505981
ERR505982
ERR505983
ERR505984
ERR505985
ERR505986
ERR505987
ERR505996
ERR505997
ERR505998
ERR505999
ERR506000
ERR506002

ERR506003
ERR505988
ERR506006
ERR506008
ERR506011
ERR505989
ERR506013
ERR506018
ERR506019
ERR506020
ERR505990
ERR506021
ERR506022
ERR506023
ERR506024
ERR506025
ERR506026
ERR506027
ERR506028
ERR506030
ERR506031
ERR506032
ERR506034
ERR506035
ERR506036
ERR505992
ERR506038
ERR506039
ERR506041
ERR506043
ERR506046
ERR506047
ERR505993
ERR506048
ERR506049
ERR506050
ERR506051
ERR506052
ERR506055
ERR505994
ERR506057
ERR506058
ERR506061
ERR506062
ERR506063
ERR506064
ERR506065
ERR506066
ERR506067
ERR516618

ERR516631
ERR516632
ERR516633
ERR516634
ERR516636
ERR516639
ERR516621
ERR516647
ERR516650
ERR516655
ERR516656
ERR516657
ERR516658
ERR516659
ERR516660
ERR516662
ERR516665
ERR516623
ERR516668
ERR516669
ERR516670
ERR516672
ERR516673
ERR516675
ERR516680
ERR516681
ERR516682
ERR516683
ERR516684
ERR516689
ERR516691
ERR516626
ERR516692
ERR568435
ERR568442
ERR568443
ERR568444
ERR568445
ERR568446
ERR568447
ERR568448
ERR568449
ERR568450
ERR568451
ERR568452
ERR568453
ERR568454
ERR568455
ERR568456
ERR568457

ERR568458
ERR568459
ERR568460
ERR568436
ERR568461
ERR568462
ERR568463
ERR568464
ERR568465
ERR568466
ERR568467
ERR568468
ERR568469
ERR568470
ERR568437
ERR568471
ERR568472
ERR568473
ERR568474
ERR568475
ERR568476
ERR568477
ERR568478
ERR568479
ERR568481
ERR568482
ERR568483
ERR568484
ERR568485
ERR568486
ERR568487
ERR568488
ERR568489
ERR568438
ERR568490
ERR568491
ERR568492
ERR568493
ERR568494
ERR568495
ERR568496
ERR568497
ERR568498
ERR568499
ERR568439
ERR568500
ERR568501
ERR568502
ERR568504
ERR568505

ERR568506
ERR568507
ERR568508
ERR568440
ERR568510
ERR568512
ERR568513
ERR568514
ERR568515
ERR568516
ERR568517
ERR568441
ERR568518
ERR568527
ERR568528
ERR568529
ERR568530
ERR568531
ERR568532
ERR568533
ERR568535
ERR568536
ERR568537
ERR568538
ERR568539
ERR568540
ERR568541
ERR568543
ERR568544
ERR568545
ERR568546
ERR568520
ERR568547
ERR568548
ERR568549
ERR568550
ERR568551
ERR568552
ERR568553
ERR568554
ERR568555
ERR568556
ERR568521
ERR568557
ERR568558
ERR568559
ERR568560
ERR568562
ERR568563
ERR568564

ERR568565
ERR568566
ERR568567
ERR568568
ERR568570
ERR568571
ERR568572
ERR568573
ERR568574
ERR568575
ERR568576
ERR568523
ERR568577
ERR568578
ERR568579
ERR568580
ERR568582
ERR568583
ERR568584
ERR568585
ERR568586
ERR568524
ERR568587
ERR568589
ERR568590
ERR568591
ERR568592
ERR568593
ERR568594
ERR568595
ERR568596
ERR568525
ERR568598
ERR568599
ERR568600
ERR568601
ERR568603
ERR568604
ERR568605
ERR568526
ERR568606
ERR568616
ERR568618
ERR568619
ERR568620
ERR568621
ERR568622
ERR568623
ERR568624
ERR568607

ERR568625
ERR568626
ERR568627
ERR568628
ERR568629
ERR568630
ERR568631
ERR568632
ERR568633
ERR568608
ERR568634
ERR568635
ERR568636
ERR568637
ERR568638
ERR568639
ERR568640
ERR568641
ERR568643
ERR568644
ERR568645
ERR568646
ERR568647
ERR568648
ERR568649
ERR568650
ERR568651
ERR568610
ERR568652
ERR568654
ERR568656
ERR568657
ERR568658
ERR568659
ERR568660
ERR568661
ERR568611
ERR568662
ERR568663
ERR568664
ERR568665
ERR568666
ERR568667
ERR568668
ERR568669
ERR568670
ERR568671
ERR568672
ERR568673
ERR568674

ERR568675
ERR568676
ERR568677
ERR568678
ERR568679
ERR568680
ERR568681
ERR568682
ERR568683
ERR568684
ERR568685
ERR568686
ERR568687
ERR568688
ERR568689
ERR568690
ERR568696
ERR568697
ERR568698
ERR568700
ERR568701
ERR568702
ERR568703
ERR568704
ERR568705
ERR568691
ERR568706
ERR568707
ERR568708
ERR568709
ERR568710
ERR568711
ERR568712
ERR568713
ERR568714
ERR568715
ERR568692
ERR568716
ERR568717
ERR568718
ERR568719
ERR568720
ERR568721
ERR568722
ERR568723
ERR568724
ERR568725
ERR568693
ERR568726
ERR568727

ERR568728
ERR568729
ERR568730
ERR568731
ERR568732
ERR568733
ERR568735
ERR568694
ERR568736
ERR568737
ERR568738
ERR568739
ERR568740
ERR568741
ERR568742
ERR568743
ERR568744
ERR568745
ERR568695
ERR568746
ERR568747
ERR568748
ERR568749
ERR570377
ERR570378
ERR570379
ERR570374
ERR570383
ERR570384
ERR570385
ERR570386
ERR570387
ERR570389
ERR570390
ERR570391
ERR570392
ERR570375
ERR570393
ERR570394
ERR570395
ERR570397
ERR570398
ERR570399
ERR570400
ERR570401
ERR570376
ERR570402
ERR570411
ERR570412
ERR570413

ERR570414
ERR570415
ERR570416
ERR570417
ERR570418
ERR570419
ERR570420
ERR570403
ERR570421
ERR570422
ERR570423
ERR570424
ERR570425
ERR570426
ERR570427
ERR570428
ERR570429
ERR570430
ERR570431
ERR570432
ERR570433
ERR570434
ERR570435
ERR570436
ERR570437
ERR570438
ERR570439
ERR570405
ERR570442
ERR570443
ERR570444
ERR570445
ERR570446
ERR570447
ERR570448
ERR570406
ERR570449
ERR570450
ERR570451
ERR570452
ERR570453
ERR570454
ERR570455
ERR570456
ERR570457
ERR570458
ERR570407
ERR570459
ERR570460
ERR570461

ERR570462
ERR570463
ERR570464
ERR570466
ERR570467
ERR570468
ERR570408
ERR570469
ERR570470
ERR570471
ERR570472
ERR570473
ERR570474
ERR570476
ERR570477
ERR570478
ERR570409
ERR570479
ERR570480
ERR570410
ERR568750
ERR568751
ERR568752
ERR568755
ERR568756
ERR568758
ERR568759
ERR568760
ERR568762
ERR568764
ERR568765
ERR568766
ERR568767
ERR568768
ERR568770
ERR568771
ERR568773
ERR568776
ERR568777
ERR577863
ERR577856
ERR577890
ERR577900
ERR577859
ERR577918
ERR577924
ERR577927
ERR577929
ERR577930
ERR577861

ERR577944
ERR577955
ERR577956
ERR577957
ERR577961
ERR577945
ERR577963
ERR577964
ERR577969
ERR577971
ERR577977
ERR577979
ERR577981
ERR577984
ERR577986
ERR577991
ERR577992
ERR577995
ERR577999
ERR578002
ERR578005
ERR578007
ERR578009
ERR578010
ERR577950
ERR578014
ERR578017
ERR578022
ERR578023
ERR577952
ERR578031
ERR586313
ERR586316
ERR586317
ERR586326
ERR586327
ERR586328
ERR586331
ERR586334
ERR586335
ERR586339
ERR586340
ERR586342
ERR586343
ERR586344
ERR586345
ERR586347
ERR586348
ERR586349
ERR586350

ERR586351
ERR586352
ERR586353
ERR586355
ERR586357
ERR586358
ERR586359
ERR586360
ERR586363
ERR586364
ERR586367
ERR586368
ERR586375
ERR586376
ERR586377
ERR586378
ERR586379
ERR586380
ERR586381
ERR586382
ERR586394
ERR586395
ERR586396
ERR586399
ERR586402
ERR586406
ERR586411
ERR586413
ERR586414
ERR586415
ERR586417
ERR586420
ERR586421
ERR586422
ERR586423
ERR586424
ERR586425
ERR586426
ERR586428
ERR586431
ERR586388
ERR586440
ERR586441
ERR586443
ERR586444
ERR586445
ERR586447
ERR586448
ERR586449
ERR586450

ERR586451
ERR586454
ERR586455
ERR586456
ERR586458
ERR586459
ERR586468
ERR586460
ERR586461
ERR586462
ERR586469
ERR586471
ERR586472
ERR586463
ERR586464
ERR586465
ERR586466
ERR596384
ERR596385
ERR596386
ERR596387
ERR596388
ERR596389
ERR596390
ERR596391
ERR596377
ERR596392
ERR596393
ERR596394
ERR596395
ERR596396
ERR596397
ERR596378
ERR596398
ERR596399
ERR596400
ERR596401
ERR596402
ERR596403
ERR596404
ERR596405
ERR596406
ERR596407
ERR596379
ERR596408
ERR596409
ERR596410
ERR596411
ERR596412
ERR596413

ERR596414
ERR596380
ERR596415
ERR596416
ERR596417
ERR596418
ERR596381
ERR596419
ERR596420
ERR596421
ERR596422
ERR596382
ERR596423
ERR596424
ERR600078
ERR600087
ERR600088
ERR600089
ERR600090
ERR600091
ERR600092
ERR600093
ERR600079
ERR600094
ERR600095
ERR600096
ERR600097
ERR600098
ERR600099
ERR600100
ERR600101
ERR600080
ERR600103
ERR600104
ERR600105
ERR600106
ERR600107
ERR600108
ERR600081
ERR600110
ERR600111
ERR600112
ERR600113
ERR600114
ERR600082
ERR600115
ERR600116
ERR600117
ERR600083
ERR600118

ERR600119
ERR600120
ERR600122
ERR600123
ERR600124
ERR600084
ERR600125
ERR600126
ERR600127
ERR600128
ERR600129
ERR600130
ERR600131
ERR600132
ERR600085
ERR600133
ERR600134
ERR600135
ERR600136
ERR600137
ERR600138
ERR600139
ERR600140
ERR600141
ERR600142
ERR600086
ERR600143
ERR600144
ERR600145
ERR600146
ERR600153
ERR600154
ERR600155
ERR600156
ERR600157
ERR600158
ERR600147
ERR600159
ERR600160
ERR600161
ERR600162
ERR600163
ERR600164
ERR600166
ERR600148
ERR600167
ERR600168
ERR600169
ERR600170
ERR600171

ERR600172
ERR600173
ERR600174
ERR600149
ERR600175
ERR600176
ERR600177
ERR600178
ERR600179
ERR600180
ERR600181
ERR600182
ERR600183
ERR600184
ERR600185
ERR600187
ERR600188
ERR600189
ERR600190
ERR600191
ERR600192
ERR600150
ERR600193
ERR600194
ERR600195
ERR600196
ERR600197
ERR600198
ERR600199
ERR600200
ERR600201
ERR600202
ERR600151
ERR600203
ERR600204
ERR600205
ERR600206
ERR600207
ERR600208
ERR600209
ERR600210
ERR600211
ERR600212
ERR600213
ERR600214
ERR600215
ERR600216
ERR600217
ERR600218
ERR600219

ERR600220
ERR600152
ERR600221
ERR600222
ERR600223
ERR600224
ERR600225
ERR600226
ERR600232
ERR600233
ERR600234
ERR600235
ERR600236
ERR600237
ERR600238
ERR600239
ERR600240
ERR600241
ERR600242
ERR600243
ERR600244
ERR600245
ERR600246
ERR600247
ERR600248
ERR600227
ERR600249
ERR600250
ERR600251
ERR600252
ERR600253
ERR600254
ERR600255
ERR600228
ERR600256
ERR600257
ERR600258
ERR600259
ERR600261
ERR600262
ERR600229
ERR600264
ERR600265
ERR600230
ERR600266
ERR600267
ERR600268
ERR600269
ERR600270
ERR600271

ERR600272
ERR600273
ERR600274
ERR600275
ERR600231
ERR600276
ERR600277
ERR600278
ERR600280
ERR600281
ERR600282
ERR600285
ERR600287
ERR600288
ERR600289
ERR600290
ERR600291
ERR600292
ERR600293
ERR600294
ERR600295
ERR600296
ERR600297
ERR600298
ERR600299
ERR600300
ERR600301
ERR600302
ERR600303
ERR600304
ERR600305
ERR600306
ERR600307
ERR600308
ERR600284
ERR632868
ERR632877
ERR632878
ERR632880
ERR632882
ERR632883
ERR632884
ERR632885
ERR632886
ERR632869
ERR632887
ERR632888
ERR632889
ERR632890
ERR632891

ERR632892
ERR632893
ERR632894
ERR632895
ERR632896
ERR632870
ERR632897
ERR632898
ERR632899
ERR632900
ERR632901
ERR632902
ERR632903
ERR632905
ERR632906
ERR632871
ERR632907
ERR632908
ERR632909
ERR632910
ERR632911
ERR632912
ERR632913
ERR632914
ERR632916
ERR632872
ERR632917
ERR632918
ERR632919
ERR632920
ERR632921
ERR632922
ERR632923
ERR632924
ERR632925
ERR632926
ERR632873
ERR632927
ERR632928
ERR632929
ERR632930
ERR632932
ERR632933
ERR632934
ERR632935
ERR632936
ERR632874
ERR632937
ERR632938
ERR632939

ERR632940
ERR632941
ERR632942
ERR632943
ERR632944
ERR632945
ERR632946
ERR632875
ERR632947
ERR632948
ERR632949
ERR632950
ERR632951
ERR632952
ERR632953
ERR632954
ERR632955
ERR632956
ERR632876
ERR632957
ERR632958
ERR632959
ERR632960
ERR632961
ERR632962
ERR600312
ERR600320
ERR600321
ERR600322
ERR600323
ERR600324
ERR600325
ERR600326
ERR600327
ERR600329
ERR600313
ERR600330
ERR600331
ERR600332
ERR600333
ERR600334
ERR600335
ERR600336
ERR600337
ERR600338
ERR600339
ERR600314
ERR600340
ERR600341
ERR600342

ERR600343
ERR600344
ERR600345
ERR600346
ERR600348
ERR600349
ERR600315
ERR600350
ERR600351
ERR600352
ERR600353
ERR600354
ERR600355
ERR600356
ERR600357
ERR600359
ERR600316
ERR600360
ERR600361
ERR600362
ERR600364
ERR600365
ERR600366
ERR600367
ERR600368
ERR600317
ERR600370
ERR600371
ERR600372
ERR600374
ERR600375
ERR600376
ERR600377
ERR600378
ERR600379
ERR600318
ERR600380
ERR600381
ERR600382
ERR600383
ERR600384
ERR600385
ERR600386
ERR600387
ERR600388
ERR600319
ERR600389
ERR600390
ERR600391
ERR600392

ERR600393
ERR600394
ERR600395
ERR600396
ERR600397
ERR600398
ERR600399
ERR600400
ERR600401
ERR600402
ERR600403
ERR600404
ERR600405
ERR600406
ERR600414
ERR600415
ERR600416
ERR600417
ERR600418
ERR600419
ERR600420
ERR600421
ERR600422
ERR600423
ERR600407
ERR600424
ERR600425
ERR600426
ERR600427
ERR600428
ERR600429
ERR600430
ERR600431
ERR600432
ERR600433
ERR600408
ERR600434
ERR600435
ERR600437
ERR600438
ERR600439
ERR600440
ERR600441
ERR600442
ERR600443
ERR600409
ERR600445
ERR600446
ERR600447
ERR600448

ERR600449
ERR600450
ERR600451
ERR600452
ERR600453
ERR600410
ERR600455
ERR600456
ERR600457
ERR600458
ERR600459
ERR600460
ERR600461
ERR600462
ERR600463
ERR600411
ERR600464
ERR600465
ERR600466
ERR600467
ERR600468
ERR600469
ERR600470
ERR600471
ERR600472
ERR600473
ERR600412
ERR600474
ERR600475
ERR600476
ERR600477
ERR600478
ERR600479
ERR600480
ERR600481
ERR600482
ERR600483
ERR600413
ERR600484
ERR600485
ERR600486
ERR600487
ERR600488
ERR600489
ERR600490
ERR600491
ERR600492
ERR600493
ERR600494
ERR600495

ERR600496
ERR600497
ERR600498
ERR600499
ERR600500
ERR600501
ERR600510
ERR600511
ERR600512
ERR600513
ERR600514
ERR600515
ERR600516
ERR600517
ERR600518
ERR600519
ERR600502
ERR600520
ERR600521
ERR600522
ERR600523
ERR600524
ERR600525
ERR600526
ERR600527
ERR600528
ERR600529
ERR600503
ERR600530
ERR600531
ERR600532
ERR600533
ERR600534
ERR600535
ERR600536
ERR600537
ERR600538
ERR600539
ERR600504
ERR600540
ERR600541
ERR600542
ERR600543
ERR600544
ERR600545
ERR600546
ERR600547
ERR600548
ERR600549
ERR600505

ERR600550
ERR600551
ERR600552
ERR600553
ERR600554
ERR600555
ERR600556
ERR600557
ERR600558
ERR600559
ERR600560
ERR600561
ERR600562
ERR600563
ERR600564
ERR600565
ERR600566
ERR600567
ERR600568
ERR600569
ERR600570
ERR600571
ERR600573
ERR600574
ERR600575
ERR600576
ERR600577
ERR600508
ERR600579
ERR600580
ERR600581
ERR600582
ERR600583
ERR600584
ERR600585
ERR600586
ERR600587
ERR600588
ERR600509
ERR600589
ERR600590
ERR600591
ERR600592
ERR600593
ERR600594
ERR600595
ERR600596
ERR600599
ERR600600
ERR600601

ERR600603
ERR600604
ERR600605
ERR600606
ERR600607
ERR600608
ERR600597
ERR600609
ERR600612
ERR600613
ERR600614
ERR600615
ERR600616
ERR600617
ERR600618
ERR600598
ERR600619
ERR600620
ERR600622
ERR600623
ERR600624
ERR600625
ERR600627
ERR600628
ERR632963
ERR632969
ERR632970
ERR632971
ERR632972
ERR632973
ERR632974
ERR632975
ERR632976
ERR632977
ERR632978
ERR632964
ERR632979
ERR632980
ERR632981
ERR632982
ERR632983
ERR632984
ERR632985
ERR632986
ERR632987
ERR632988
ERR632965
ERR632989
ERR632990
ERR632991

ERR632992
ERR632993
ERR632994
ERR632995
ERR632996
ERR632997
ERR632998
ERR632966
ERR632999
ERR633000
ERR633001
ERR633002
ERR633003
ERR633004
ERR633005
ERR633006
ERR633007
ERR633008
ERR633009
ERR633010
ERR633011
ERR633012
ERR633013
ERR633014
ERR633015
ERR633016
ERR633017
ERR633018
ERR632968
ERR633019
ERR633020
ERR633021
ERR633022
ERR633023
ERR633024
ERR633025
ERR633026
ERR633033
ERR633034
ERR633035
ERR633036
ERR633037
ERR633038
ERR633039
ERR633040
ERR633041
ERR633042
ERR633027
ERR633043
ERR633044

ERR633045
ERR633046
ERR633047
ERR633048
ERR633049
ERR633050
ERR633051
ERR633052
ERR633028
ERR633053
ERR633054
ERR633056
ERR633057
ERR633058
ERR633059
ERR633060
ERR633061
ERR633062
ERR633063
ERR633064
ERR633065
ERR633066
ERR633067
ERR633068
ERR633069
ERR633070
ERR633071
ERR633072
ERR633029
ERR633073
ERR633074
ERR633075
ERR633076
ERR633077
ERR633078
ERR633079
ERR633080
ERR633081
ERR633030
ERR633082
ERR633083
ERR633084
ERR633085
ERR633086
ERR633087
ERR633088
ERR633089
ERR633090
ERR633091
ERR633031

ERR633092
ERR633093
ERR633095
ERR633096
ERR633097
ERR633099
ERR633100
ERR633101
ERR633032
ERR633102
ERR633103
ERR633104
ERR633105
ERR633106
ERR633107
ERR633108
ERR633109
ERR633110
ERR633111
ERR633112
ERR633113
ERR633114
ERR633115
ERR633116
ERR633117
ERR633118
ERR646531
ERR646539
ERR646540
ERR646541
ERR646542
ERR646543
ERR646544
ERR646545
ERR646546
ERR646548
ERR646532
ERR646549
ERR646550
ERR646551
ERR646552
ERR646553
ERR646554
ERR646555
ERR646556
ERR646557
ERR646558
ERR646533
ERR646559
ERR646560

ERR646561
ERR646563
ERR646564
ERR646565
ERR646566
ERR646567
ERR646568
ERR646534
ERR646569
ERR646570
ERR646572
ERR646573
ERR646574
ERR646575
ERR646576
ERR646577
ERR646578
ERR646535
ERR646579
ERR646580
ERR646581
ERR646582
ERR646583
ERR646584
ERR646585
ERR646586
ERR646587
ERR646588
ERR646536
ERR646589
ERR646590
ERR646591
ERR646592
ERR646593
ERR646594
ERR646595
ERR646596
ERR646597
ERR646598
ERR646599
ERR646600
ERR646601
ERR646602
ERR646603
ERR646604
ERR646605
ERR646606
ERR646607
ERR646608
ERR646537

ERR646609
ERR646610
ERR646611
ERR646612
ERR646613
ERR646614
ERR646615
ERR646616
ERR646617
ERR646618
ERR646538
ERR646619
ERR662294
ERR662295
ERR662296
ERR662300
ERR662305
ERR662310
ERR662311
ERR662317
ERR662326
ERR662327
ERR662328
ERR662329
ERR662330
ERR662331
ERR662332
ERR662333
ERR662334
ERR662335
ERR662318
ERR662336
ERR662337
ERR662338
ERR662339
ERR662340
ERR662341
ERR662342
ERR662343
ERR662344
ERR662345
ERR662319
ERR662346
ERR662347
ERR662348
ERR662349
ERR662350
ERR662351
ERR662352
ERR662354

ERR662355
ERR662356
ERR662357
ERR662358
ERR662359
ERR662360
ERR662361
ERR662362
ERR662363
ERR662364
ERR662365
ERR662321
ERR662367
ERR662368
ERR662369
ERR662370
ERR662372
ERR662373
ERR662374
ERR662375
ERR662322
ERR662376
ERR662377
ERR662378
ERR662379
ERR662380
ERR662382
ERR662383
ERR662384
ERR662385
ERR662323
ERR662386
ERR662387
ERR662388
ERR662324
ERR662325
ERR662389
ERR662398
ERR662399
ERR662400
ERR662401
ERR662402
ERR662403
ERR662404
ERR662405
ERR662406
ERR662407
ERR662390
ERR662408
ERR662409

ERR662410
ERR662412
ERR662413
ERR662414
ERR662415
ERR662416
ERR662417
ERR662391
ERR662418
ERR662419
ERR662420
ERR662421
ERR662422
ERR662423
ERR662424
ERR662425
ERR662426
ERR662427
ERR662392
ERR662428
ERR662429
ERR662430
ERR662431
ERR662432
ERR662433
ERR662434
ERR662435
ERR662436
ERR662437
ERR662393
ERR662438
ERR662439
ERR662440
ERR662441
ERR662442
ERR662443
ERR662444
ERR662445
ERR662446
ERR662447
ERR662394
ERR662448
ERR662449
ERR662450
ERR662451
ERR662452
ERR662453
ERR662454
ERR662455
ERR662456

ERR662457
ERR662395
ERR662458
ERR662459
ERR662460
ERR662396
ERR701778
ERR701787
ERR701788
ERR701792
ERR701793
ERR701794
ERR701795
ERR701796
ERR701779
ERR701797
ERR701798
ERR701799
ERR701800
ERR701801
ERR701802
ERR701803
ERR701804
ERR701805
ERR701806
ERR701780
ERR701807
ERR701808
ERR701809
ERR701810
ERR701811
ERR701812
ERR701815
ERR701816
ERR701781
ERR701817
ERR701818
ERR701819
ERR701820
ERR701821
ERR701822
ERR701823
ERR701824
ERR701825
ERR701826
ERR701782
ERR701827
ERR701828
ERR701829
ERR701830

ERR701831
ERR701832
ERR701833
ERR701834
ERR701835
ERR701836
ERR701783
ERR701837
ERR701838
ERR701839
ERR701841
ERR701842
ERR701843
ERR701844
ERR701845
ERR701846
ERR701784
ERR701847
ERR701848
ERR701849
ERR701850
ERR701851
ERR701852
ERR701853
ERR701855
ERR701856
ERR701785
ERR701857
ERR701858
ERR701859
ERR701860
ERR701861
ERR701862
ERR701863
ERR701864
ERR701865
ERR701866
ERR701786
ERR701867
ERR701868
ERR701869
ERR701870
ERR701871
ERR701872
ERR708297
ERR708298
ERR708299
ERR708300
ERR708301
ERR708302

ERR708303
ERR714103
ERR714112
ERR714113
ERR714114
ERR714118
ERR714119
ERR714120
ERR714121
ERR714104
ERR714122
ERR714123
ERR714124
ERR714125
ERR714126
ERR714127
ERR714131
ERR714105
ERR714132
ERR714133
ERR714135
ERR714136
ERR714141
ERR714142
ERR714148
ERR714149
ERR714153
ERR714156
ERR714157
ERR714158
ERR714161
ERR714108
ERR714170
ERR714171
ERR714109
ERR714172
ERR714173
ERR714174
ERR714175
ERR714176
ERR714177
ERR714178
ERR714179
ERR714180
ERR714181
ERR714110
ERR714182
ERR714183
ERR714184
ERR714185

ERR714186
ERR714187
ERR714111
ERR714188
ERR714197
ERR714198
ERR714199
ERR714201
ERR714203
ERR714204
ERR714205
ERR714206
ERR714189
ERR714207
ERR714208
ERR714209
ERR714210
ERR714211
ERR714212
ERR714213
ERR714214
ERR714215
ERR714216
ERR714190
ERR714217
ERR714218
ERR714220
ERR714221
ERR714222
ERR714225
ERR714226
ERR714191
ERR714227
ERR714228
ERR714229
ERR714230
ERR714231
ERR714232
ERR714234
ERR714235
ERR714236
ERR714192
ERR714237
ERR714240
ERR714245
ERR714246
ERR714193
ERR714247
ERR714250
ERR714252

ERR714253
ERR714194
ERR714258
ERR714266
ERR714195
ERR714273
ERR714282
ERR714283
ERR714284
ERR714285
ERR714286
ERR714287
ERR714288
ERR714289
ERR714290
ERR714274
ERR714293
ERR714294
ERR714295
ERR714296
ERR714297
ERR714298
ERR714299
ERR714300
ERR714301
ERR714275
ERR714302
ERR714305
ERR714306
ERR714307
ERR714308
ERR714309
ERR714310
ERR714311
ERR714276
ERR714312
ERR714313
ERR714314
ERR714317
ERR714318
ERR714319
ERR714320
ERR714277
ERR714323
ERR714324
ERR714330
ERR714331
ERR714340
ERR714341
ERR714279

ERR714342
ERR714344
ERR714346
ERR714350
ERR714351
ERR714280
ERR714353
ERR714355
ERR714356
ERR714281
ERR714358
ERR714367
ERR714368
ERR714369
ERR714371
ERR714372
ERR714373
ERR714374
ERR714376
ERR714359
ERR714377
ERR714378
ERR714379
ERR714380
ERR714381
ERR714383
ERR714384
ERR714385
ERR714387
ERR714388
ERR714389
ERR714390
ERR714391
ERR714392
ERR714395
ERR714398
ERR714401
ERR714403
ERR714404
ERR714406
ERR714362
ERR714408
ERR714409
ERR714411
ERR714412
ERR714413
ERR714414
ERR714363
ERR714417
ERR714418

ERR714419
ERR714421
ERR714422
ERR714423
ERR714425
ERR714364
ERR714428
ERR714429
ERR714430
ERR714431
ERR714433
ERR714434
ERR714435
ERR714436
ERR714365
ERR714437
ERR714438
ERR714440
ERR714441
ERR714366
ERR714443
ERR714452
ERR714453
ERR714454
ERR714455
ERR714456
ERR714457
ERR714458
ERR714459
ERR714460
ERR714461
ERR714444
ERR714462
ERR714463
ERR714464
ERR714465
ERR714466
ERR714467
ERR714468
ERR714469
ERR714470
ERR714471
ERR714445
ERR714472
ERR714473
ERR714474
ERR714475
ERR714477
ERR714478
ERR714479

ERR714480
ERR714481
ERR714446
ERR714482
ERR714483
ERR714484
ERR714485
ERR714486
ERR714487
ERR714488
ERR714489
ERR714490
ERR714491
ERR714447
ERR714492
ERR714493
ERR714494
ERR714495
ERR714497
ERR714498
ERR714499
ERR714501
ERR714448
ERR714502
ERR714503
ERR714504
ERR714505
ERR714506
ERR714507
ERR714508
ERR714509
ERR714510
ERR714511
ERR714449
ERR714512
ERR714513
ERR714514
ERR714515
ERR714516
ERR714517
ERR714518
ERR714519
ERR714520
ERR714521
ERR714522
ERR714523
ERR714524
ERR714525
ERR714526
ERR714527

ERR714528
ERR714529
ERR714530
ERR714531
ERR714451
ERR714532
ERR714533
ERR714534
ERR714535
ERR714536
ERR714538
ERR714547
ERR714548
ERR714549
ERR714550
ERR714551
ERR714552
ERR714553
ERR714554
ERR714555
ERR714556
ERR714539
ERR714558
ERR714559
ERR714560
ERR714561
ERR714562
ERR714563
ERR714564
ERR714565
ERR714566
ERR714540
ERR714567
ERR714568
ERR714569
ERR714570
ERR714571
ERR714572
ERR714574
ERR714575
ERR714576
ERR714541
ERR714577
ERR714578
ERR714579
ERR714580
ERR714581
ERR714582
ERR714583
ERR714585

ERR714586
ERR714542
ERR714587
ERR714588
ERR714589
ERR714590
ERR714591
ERR714593
ERR714594
ERR714595
ERR714596
ERR714543
ERR714597
ERR714598
ERR714599
ERR714600
ERR714602
ERR714603
ERR714604
ERR714605
ERR714606
ERR714544
ERR714607
ERR714608
ERR714609
ERR714610
ERR714611
ERR714612
ERR714613
ERR714614
ERR714615
ERR714545
ERR714617
ERR714618
ERR714620
ERR714621
ERR714622
ERR714623
ERR714624
ERR714625
ERR714626
ERR714546
ERR714627
ERR714628
ERR714629
ERR714630
ERR714631
ERR714632
ERR714633
ERR714642

ERR714643
ERR714644
ERR714645
ERR714646
ERR714647
ERR714648
ERR714649
ERR714650
ERR714651
ERR714634
ERR714652
ERR714653
ERR714654
ERR714655
ERR714656
ERR714657
ERR714658
ERR714659
ERR714660
ERR714661
ERR714635
ERR714662
ERR714663
ERR714664
ERR714665
ERR714666
ERR714667
ERR714668
ERR714669
ERR714670
ERR714671
ERR714636
ERR714672
ERR714673
ERR714674
ERR714675
ERR714676
ERR714677
ERR714678
ERR714679
ERR714680
ERR714681
ERR714637
ERR714682
ERR714683
ERR714684
ERR714685
ERR714686
ERR714687
ERR714688

ERR714689
ERR714690
ERR714691
ERR714638
ERR714692
ERR714693
ERR714694
ERR714695
ERR714696
ERR714697
ERR714698
ERR714699
ERR714700
ERR714639
ERR714702
ERR714703
ERR714704
ERR714705
ERR714706
ERR714707
ERR714709
ERR714710
ERR714711
ERR714640
ERR714712
ERR714713
ERR714714
ERR714715
ERR714716
ERR714717
ERR714718
ERR714719
ERR714720
ERR714721
ERR714641
ERR714722
ERR714723
ERR714724
ERR714725
ERR714726
ERR714727
ERR730141
ERR730150
ERR730151
ERR730152
ERR730153
ERR730154
ERR730155
ERR730156
ERR730157

ERR730158
ERR730159
ERR730142
ERR730160
ERR730161
ERR730162
ERR730163
ERR730164
ERR730165
ERR730166
ERR730167
ERR730168
ERR730169
ERR730143
ERR730170
ERR730171
ERR730172
ERR730173
ERR730174
ERR730175
ERR730176
ERR730177
ERR730178
ERR730179
ERR730144
ERR730180
ERR730181
ERR730182
ERR730183
ERR730184
ERR730185
ERR730186
ERR730187
ERR730188
ERR730189
ERR730145
ERR730190
ERR730191
ERR730192
ERR730193
ERR730194
ERR730195
ERR730196
ERR730197
ERR730198
ERR730199
ERR730146
ERR730200
ERR730201
ERR730202

ERR730203
ERR730204
ERR730205
ERR730206
ERR730207
ERR730208
ERR730209
ERR730147
ERR730210
ERR730211
ERR730212
ERR730213
ERR730214
ERR730215
ERR730216
ERR730217
ERR730218
ERR730219
ERR730148
ERR730221
ERR730222
ERR730223
ERR730224
ERR730225
ERR730226
ERR730149
ERR730228
ERR730229
ERR730230
ERR730231
ERR730232
ERR730233
ERR730234
ERR730243
ERR730244
ERR730245
ERR730246
ERR730247
ERR730248
ERR730249
ERR730250
ERR730251
ERR730252
ERR730235
ERR730253
ERR730254
ERR730255
ERR730256
ERR730257
ERR730258

ERR730259
ERR730260
ERR730261
ERR730262
ERR730236
ERR730263
ERR730264
ERR730265
ERR730266
ERR730267
ERR730268
ERR730269
ERR730270
ERR730271
ERR730272
ERR730237
ERR730273
ERR730274
ERR730275
ERR730276
ERR730277
ERR730278
ERR730279
ERR730280
ERR730281
ERR730282
ERR730238
ERR730284
ERR730285
ERR730286
ERR730287
ERR730288
ERR730289
ERR730291
ERR730292
ERR730239
ERR730293
ERR730294
ERR730295
ERR730296
ERR730297
ERR730298
ERR730299
ERR730300
ERR730301
ERR730302
ERR730240
ERR730303
ERR730304
ERR730305

ERR730306
ERR730307
ERR730308
ERR730309
ERR730310
ERR730311
ERR730312
ERR730241
ERR730314
ERR730315
ERR730316
ERR730317
ERR730318
ERR730319
ERR730320
ERR730321
ERR730322
ERR730242
ERR730323
ERR730324
ERR730325
ERR730326
ERR730327
ERR730328
ERR730338
ERR730340
ERR730341
ERR730342
ERR730343
ERR730344
ERR730345
ERR730346
ERR730347
ERR730330
ERR730348
ERR730349
ERR730350
ERR730351
ERR730352
ERR730353
ERR730354
ERR730355
ERR730356
ERR730357
ERR730331
ERR730358
ERR730359
ERR730360
ERR730361
ERR730362

ERR730363
ERR730364
ERR730365
ERR730366
ERR730367
ERR730332
ERR730368
ERR730369
ERR730370
ERR730371
ERR730372
ERR730373
ERR730376
ERR730377
ERR730333
ERR730379
ERR730380
ERR730381
ERR730382
ERR730383
ERR730334
ERR730388
ERR730389
ERR730391
ERR730392
ERR730394
ERR730397
ERR730335
ERR730399
ERR730401
ERR730404
ERR730405
ERR730406
ERR730336
ERR730409
ERR730417
ERR730337
ERR730421
ERR730433
ERR730434
ERR730435
ERR730436
ERR730438
ERR730439
ERR730440
ERR730441
ERR730442
ERR730443
ERR730444
ERR730445

ERR730446
ERR730447
ERR730448
ERR730449
ERR730450
ERR730451
ERR730452
ERR730426
ERR730453
ERR730454
ERR730455
ERR730456
ERR730457
ERR730458
ERR730459
ERR730460
ERR730461
ERR730462
ERR730427
ERR730463
ERR730464
ERR730466
ERR730467
ERR730468
ERR730469
ERR730470
ERR730471
ERR730472
ERR730428
ERR730473
ERR730474
ERR730475
ERR730476
ERR730477
ERR730479
ERR730480
ERR730481
ERR730482
ERR730483
ERR730484
ERR730485
ERR730486
ERR730487
ERR730488
ERR730489
ERR730490
ERR730491
ERR730492
ERR730430
ERR730493

ERR730495
ERR730496
ERR730497
ERR730498
ERR730499
ERR730500
ERR730501
ERR730502
ERR730503
ERR730504
ERR730508
ERR730509
ERR730512
ERR730513
ERR730514
ERR730515
ERR730517
ERR730519
ERR730528
ERR730529
ERR730530
ERR730531
ERR730532
ERR730533
ERR730534
ERR730535
ERR730536
ERR730537
ERR730520
ERR730538
ERR730539
ERR730540
ERR730541
ERR730542
ERR730543
ERR730544
ERR730545
ERR730546
ERR730547
ERR730521
ERR730548
ERR730549
ERR730550
ERR730551
ERR730552
ERR730553
ERR730555
ERR730556
ERR730557
ERR730522

ERR730558
ERR730559
ERR730560
ERR730561
ERR730562
ERR730563
ERR730564
ERR730565
ERR730566
ERR730567
ERR730523
ERR730568
ERR730569
ERR730570
ERR730571
ERR730572
ERR730573
ERR730574
ERR730575
ERR730576
ERR730577
ERR730524
ERR730579
ERR730580
ERR730581
ERR730582
ERR730583
ERR730584
ERR730585
ERR730586
ERR730587
ERR730525
ERR730588
ERR730589
ERR730590
ERR730591
ERR730592
ERR730593
ERR730594
ERR730595
ERR730596
ERR730597
ERR730526
ERR730598
ERR730599
ERR730600
ERR730601
ERR730602
ERR730603
ERR730604

ERR730605
ERR730606
ERR730607
ERR730527
ERR730608
ERR730609
ERR730610
ERR736900
ERR736901
ERR736902
ERR736903
ERR736907
ERR736908
ERR736909
ERR736910
ERR736911
ERR736912
ERR736913
ERR736914
ERR736915
ERR736916
ERR736904
ERR736917
ERR736918
ERR736919
ERR736920
ERR736921
ERR736922
ERR730616
ERR736923
ERR736924
ERR736905
ERR736925
ERR736926
ERR736927
ERR736928
ERR736930
ERR736931
ERR736932
ERR736933
ERR736934
ERR736906
ERR736935
ERR736936
ERR730618
ERR730619
ERR736937
ERR736938
ERR736939
ERR736940

ERR730620
ERR730611
ERR736941
ERR730621
ERR730622
ERR730623
ERR730624
ERR730625
ERR730626
ERR730627
ERR730628
ERR730629
ERR730612
ERR730630
ERR730631
ERR730632
ERR730634
ERR730635
ERR730636
ERR730637
ERR730638
ERR730639
ERR730613
ERR730640
ERR730641
ERR730642
ERR730643
ERR730644
ERR730645
ERR730646
ERR730647
ERR730648
ERR730649
ERR730614
ERR730650
ERR730651
ERR730652
ERR730653
ERR730654
ERR730655
ERR730656
ERR730657
ERR730658
ERR730659
ERR730660
ERR730661
ERR730662
ERR730663
ERR730664
ERR730665

ERR730666
ERR730675
ERR730676
ERR730677
ERR730678
ERR730679
ERR730680
ERR730681
ERR730682
ERR730683
ERR730684
ERR730667
ERR730685
ERR730687
ERR730688
ERR730689
ERR730690
ERR730691
ERR730692
ERR730693
ERR730694
ERR730668
ERR730695
ERR730696
ERR730697
ERR730698
ERR730699
ERR730700
ERR730701
ERR730702
ERR730704
ERR730705
ERR730706
ERR730707
ERR730708
ERR730709
ERR730710
ERR730711
ERR730712
ERR730713
ERR730714
ERR730670
ERR730715
ERR730716
ERR730717
ERR730718
ERR730719
ERR730720
ERR730721
ERR730722

ERR730723
ERR730724
ERR730671
ERR730726
ERR730727
ERR730728
ERR730729
ERR730730
ERR730731
ERR730732
ERR730733
ERR730734
ERR730672
ERR730735
ERR730736
ERR730737
ERR730738
ERR730739
ERR730740
ERR730741
ERR730742
ERR730743
ERR730744
ERR730745
ERR730746
ERR730747
ERR730748
ERR730749
ERR730750
ERR730751
ERR730752
ERR730753
ERR730754
ERR730674
ERR730755
ERR730756
ERR730757
ERR730758
ERR730759
ERR730760
ERR730761
ERR730770
ERR730771
ERR730772
ERR730773
ERR730774
ERR730775
ERR730776
ERR730777
ERR730778

ERR730779
ERR730762
ERR730780
ERR730781
ERR730782
ERR730783
ERR730784
ERR730785
ERR730786
ERR730787
ERR730788
ERR730789
ERR730763
ERR730790
ERR730791
ERR730792
ERR730793
ERR730794
ERR730795
ERR730796
ERR730797
ERR730798
ERR730799
ERR730764
ERR730800
ERR730801
ERR730802
ERR730803
ERR730804
ERR730805
ERR730806
ERR730807
ERR730808
ERR730809
ERR730765
ERR730810
ERR730811
ERR730812
ERR730813
ERR730814
ERR730815
ERR730816
ERR730817
ERR730818
ERR730819
ERR730766
ERR730820
ERR730821
ERR730822
ERR730823

ERR730824
ERR730825
ERR730826
ERR730827
ERR730828
ERR730829
ERR730767
ERR730830
ERR730831
ERR730832
ERR730833
ERR730834
ERR730835
ERR730836
ERR730837
ERR730838
ERR730839
ERR730768
ERR730840
ERR730841
ERR730842
ERR730843
ERR730844
ERR730845
ERR730846
ERR730847
ERR730848
ERR730849
ERR730769
ERR730850
ERR730852
ERR730853
ERR730854
ERR730855
ERR730856
ERR730865
ERR730866
ERR730867
ERR730868
ERR730869
ERR730870
ERR730871
ERR730872
ERR730873
ERR730874
ERR730857
ERR730875
ERR730876
ERR730877
ERR730878

ERR730879
ERR730880
ERR730881
ERR730882
ERR730883
ERR730884
ERR730858
ERR730885
ERR730886
ERR730887
ERR730888
ERR730889
ERR730890
ERR730891
ERR730892
ERR730893
ERR730894
ERR730859
ERR730895
ERR730896
ERR730897
ERR730898
ERR730899
ERR730900
ERR730901
ERR730902
ERR730903
ERR730904
ERR730860
ERR730905
ERR730906
ERR730907
ERR730908
ERR730909
ERR730910
ERR730911
ERR730912
ERR730913
ERR730914
ERR730861
ERR730915
ERR730916
ERR730917
ERR730918
ERR730919
ERR730920
ERR730921
ERR730922
ERR730923
ERR730924

ERR730862
ERR730925
ERR730926
ERR730927
ERR730928
ERR730929
ERR730930
ERR730931
ERR730932
ERR730934
ERR730863
ERR730935
ERR730936
ERR730937
ERR730938
ERR730939
ERR730940
ERR730941
ERR730942
ERR730943
ERR730944
ERR730864
ERR730945
ERR730946
ERR730947
ERR730948
ERR730949
ERR730950
ERR742334
ERR742343
ERR742344
ERR742345
ERR742346
ERR742347
ERR742348
ERR742349
ERR742350
ERR742351
ERR742335
ERR742352
ERR742353
ERR742354
ERR742355
ERR742356
ERR742357
ERR742358
ERR742336
ERR742359
ERR742360
ERR742361

ERR742362
ERR742363
ERR742364
ERR742365
ERR742366
ERR742337
ERR742367
ERR742368
ERR742369
ERR742370
ERR742371
ERR742372
ERR742373
ERR742374
ERR742338
ERR742375
ERR742376
ERR742377
ERR742378
ERR742379
ERR742380
ERR742381
ERR742382
ERR742383
ERR742384
ERR742339
ERR742385
ERR742386
ERR742387
ERR742388
ERR742389
ERR742390
ERR742391
ERR742392
ERR742393
ERR742394
ERR742340
ERR742395
ERR742396
ERR742399
ERR742400
ERR742401
ERR742402
ERR742403
ERR742404
ERR742341
ERR742405
ERR742406
ERR742407
ERR742408

ERR742409
ERR742410
ERR742411
ERR742412
ERR742413
ERR742414
ERR742342
ERR742415
ERR742417
ERR742418
ERR742419
ERR742420
ERR742421
ERR742430
ERR742431
ERR742434
ERR742435
ERR742436
ERR742437
ERR742438
ERR742422
ERR742439
ERR742440
ERR742441
ERR742442
ERR742443
ERR742444
ERR742445
ERR742446
ERR742447
ERR742423
ERR742448
ERR742449
ERR742450
ERR742451
ERR742452
ERR742453
ERR742454
ERR742455
ERR742424
ERR742457
ERR742458
ERR742459
ERR742460
ERR742461
ERR742462
ERR742463
ERR742464
ERR742425
ERR742465

ERR742466
ERR742467
ERR742468
ERR742469
ERR742470
ERR742471
ERR742472
ERR742473
ERR742426
ERR742474
ERR742475
ERR742476
ERR742477
ERR742478
ERR742479
ERR742480
ERR742481
ERR742482
ERR742427
ERR742483
ERR742484
ERR742485
ERR742486
ERR742487
ERR742488
ERR742489
ERR742490
ERR742428
ERR742491
ERR742492
ERR742493
ERR742494
ERR742495
ERR742496
ERR742497
ERR742498
ERR742499
ERR742429
ERR742500
ERR742501
ERR742502
ERR742503
ERR742504
ERR742513
ERR742514
ERR742515
ERR742517
ERR742518
ERR742520
ERR742521

ERR742522
ERR742505
ERR742523
ERR742524
ERR742525
ERR742526
ERR742527
ERR742528
ERR742529
ERR742530
ERR742531
ERR742506
ERR742532
ERR742533
ERR742534
ERR742535
ERR742536
ERR742537
ERR742538
ERR742539
ERR742540
ERR742541
ERR742507
ERR742542
ERR742543
ERR742544
ERR742545
ERR742546
ERR742547
ERR742548
ERR742549
ERR742550
ERR742508
ERR742551
ERR742552
ERR742553
ERR742554
ERR742555
ERR742556
ERR742557
ERR742558
ERR742559
ERR742560
ERR742509
ERR742561
ERR742562
ERR742563
ERR742564
ERR742565
ERR742566

ERR742567
ERR742569
ERR742570
ERR742510
ERR742571
ERR742572
ERR742573
ERR742574
ERR742575
ERR742576
ERR742577
ERR742578
ERR742579
ERR742580
ERR742511
ERR742581
ERR742582
ERR742583
ERR742584
ERR742585
ERR742586
ERR742587
ERR742588
ERR742589
ERR742512
ERR742591
ERR742592
ERR742593
ERR742594
ERR742595
ERR742596
ERR742597
ERR742606
ERR742607
ERR742608
ERR742609
ERR742610
ERR742611
ERR742612
ERR742613
ERR742614
ERR742615
ERR742598
ERR742616
ERR742617
ERR742618
ERR742619
ERR742620
ERR742621
ERR742622

ERR742623
ERR742624
ERR742625
ERR742599
ERR742626
ERR742627
ERR742628
ERR742629
ERR742630
ERR742631
ERR742632
ERR742633
ERR742634
ERR742600
ERR742636
ERR742637
ERR742638
ERR742639
ERR742640
ERR742641
ERR742642
ERR742643
ERR742644
ERR742645
ERR742601
ERR742646
ERR742647
ERR742648
ERR742649
ERR742650
ERR742651
ERR742652
ERR742653
ERR742654
ERR742655
ERR742602
ERR742656
ERR742657
ERR742658
ERR742659
ERR742660
ERR742662
ERR742663
ERR742664
ERR742665
ERR742603
ERR742666
ERR742667
ERR742668
ERR742669

ERR742671
ERR742672
ERR742673
ERR742674
ERR742604
ERR742675
ERR742676
ERR742677
ERR742678
ERR742679
ERR742680
ERR742681
ERR742605
ERR750790
ERR750791
ERR750792
ERR750793
ERR750794
ERR750795
ERR750796
ERR750797
ERR750798
ERR750799
ERR750800
ERR750801
ERR750802
ERR750803
ERR750804
ERR750805
ERR750806
ERR750807
ERR750808
ERR750809
ERR750810
ERR750811
ERR750812
ERR750813
ERR750814
ERR750815
ERR750816
ERR750817
ERR750818
ERR750819
ERR750820
ERR750821
ERR750822
ERR750823
ERR750824
ERR750833
ERR750834

ERR750835
ERR750836
ERR750837
ERR750838
ERR750840
ERR750841
ERR750842
ERR750825
ERR750843
ERR750844
ERR750845
ERR750846
ERR750847
ERR750848
ERR750849
ERR750850
ERR750851
ERR750852
ERR750826
ERR750853
ERR750854
ERR750860
ERR750861
ERR750862
ERR750827
ERR750863
ERR750864
ERR750865
ERR750866
ERR750828
ERR750870
ERR750873
ERR750874
ERR750875
ERR750876
ERR750877
ERR750829
ERR750879
ERR750880
ERR750881
ERR750882
ERR750883
ERR750830
ERR750831
ERR750832
ERR750962
ERR750966
ERR750970
ERR750971
ERR750972

ERR750973
ERR750974
ERR750975
ERR750976
ERR750977
ERR750978
ERR750979
ERR750980
ERR750981
ERR750982
ERR750984
ERR750985
ERR750986
ERR750987
ERR750988
ERR750989
ERR750990
ERR750991
ERR750992
ERR750993
ERR750994
ERR750995
ERR750996
ERR750997
ERR750998
ERR750999
ERR751000
ERR751001
ERR751002
ERR751011
ERR751012
ERR751013
ERR751014
ERR751015
ERR751016
ERR751017
ERR751018
ERR751019
ERR751020
ERR751021
ERR751022
ERR751024
ERR751025
ERR751026
ERR751027
ERR751028
ERR751029
ERR751030
ERR751031
ERR751032

ERR751033
ERR751034
ERR751035
ERR751036
ERR751037
ERR751038
ERR751039
ERR751040
ERR751041
ERR751042
ERR751043
ERR751044
ERR751045
ERR751046
ERR751047
ERR751048
ERR751049
ERR751050
ERR751051
ERR751052
ERR751053
ERR751054
ERR751055
ERR751056
ERR751057
ERR751058
ERR751059
ERR751060
ERR751061
ERR751062
ERR751063
ERR751064
ERR751065
ERR751066
ERR751067
ERR751068
ERR751069
ERR751070
ERR751071
ERR751072
ERR751073
ERR751074
ERR751075
ERR751076
ERR751077
ERR751078
ERR751079
ERR751080
ERR751009
ERR751081

ERR751082
ERR751083
ERR751084
ERR751086
ERR751087
ERR751088
ERR751089
ERR751010
ERR751090
ERR751091
ERR751092
ERR751093
ERR751094
ERR751095
ERR751096
ERR751097
ERR751098
ERR751099
ERR751100
ERR751101
ERR751102
ERR751103
ERR751112
ERR751113
ERR751114
ERR751115
ERR751116
ERR751118
ERR751119
ERR751120
ERR751104
ERR751122
ERR751123
ERR751124
ERR751125
ERR751126
ERR751128
ERR751129
ERR751105
ERR751132
ERR751133
ERR751134
ERR751135
ERR751136
ERR751137
ERR751138
ERR751139
ERR751140
ERR751141
ERR751106

ERR751142
ERR751143
ERR751144
ERR751145
ERR751146
ERR751147
ERR751148
ERR751149
ERR751150
ERR751151
ERR751107
ERR751152
ERR751153
ERR751154
ERR751155
ERR751156
ERR751157
ERR751158
ERR751159
ERR751160
ERR751161
ERR751108
ERR751163
ERR751164
ERR751165
ERR751166
ERR751167
ERR751168
ERR751169
ERR751170
ERR751109
ERR751171
ERR751172
ERR751173
ERR751175
ERR751176
ERR751177
ERR751178
ERR751179
ERR751180
ERR751110
ERR751182
ERR751183
ERR751184
ERR751185
ERR751186
ERR751187
ERR751189
ERR751111
ERR751190

ERR751191
ERR751192
ERR773811
ERR773812
ERR773813
ERR773814
ERR773815
ERR773824
ERR773825
ERR773826
ERR773827
ERR773828
ERR773829
ERR773830
ERR773831
ERR773832
ERR773816
ERR773834
ERR773835
ERR773836
ERR773837
ERR773838
ERR773839
ERR773840
ERR773841
ERR773842
ERR773843
ERR773817
ERR773844
ERR773845
ERR773847
ERR773848
ERR773850
ERR773851
ERR773852
ERR773853
ERR773818
ERR773854
ERR773856
ERR773857
ERR773858
ERR773859
ERR773860
ERR773861
ERR773862
ERR773863
ERR773819
ERR773864
ERR773865
ERR773866

ERR773867
ERR773868
ERR773869
ERR773870
ERR773871
ERR773873
ERR773820
ERR773874
ERR773875
ERR773876
ERR773877
ERR773878
ERR773879
ERR773880
ERR773881
ERR773882
ERR773883
ERR773821
ERR773884
ERR773885
ERR773886
ERR773887
ERR773888
ERR773889
ERR773890
ERR773892
ERR773893
ERR773822
ERR773894
ERR773895
ERR773896
ERR773897
ERR773898
ERR773899
ERR773900
ERR773901
ERR773902
ERR773903
ERR773823
ERR773904
ERR773905
ERR773906
ERR773907
ERR773908
ERR773909
ERR773910
ERR773919
ERR773920
ERR773921
ERR773922

ERR773923
ERR773924
ERR773925
ERR773926
ERR773927
ERR773928
ERR773911
ERR773929
ERR773930
ERR773932
ERR773933
ERR773934
ERR773935
ERR773936
ERR773937
ERR773938
ERR773912
ERR773939
ERR773940
ERR773941
ERR773942
ERR773943
ERR773944
ERR773945
ERR773946
ERR773947
ERR773948
ERR773913
ERR773949
ERR773950
ERR773951
ERR773952
ERR773953
ERR773954
ERR773955
ERR773956
ERR773957
ERR773958
ERR773914
ERR773959
ERR773960
ERR773962
ERR773963
ERR773964
ERR773965
ERR773966
ERR773967
ERR773968
ERR773915
ERR773969

ERR773970
ERR773971
ERR773972
ERR773973
ERR773974
ERR773975
ERR773976
ERR773977
ERR773916
ERR773978
ERR773979
ERR773980
ERR773981
ERR773982
ERR773983
ERR773984
ERR773985
ERR773986
ERR773987
ERR773917
ERR773988
ERR773989
ERR773990
ERR773991
ERR773992
ERR773993
ERR773994
ERR773995
ERR773996
ERR773997
ERR773918
ERR773998
ERR773999
ERR774000
ERR774001
ERR774002
ERR774003
ERR774004
ERR774012
ERR774013
ERR774014
ERR774015
ERR774016
ERR774017
ERR774018
ERR774019
ERR774020
ERR774021
ERR774005
ERR774022

ERR774023
ERR774024
ERR774025
ERR774026
ERR774027
ERR774028
ERR774029
ERR774030
ERR774031
ERR774006
ERR774032
ERR774033
ERR774034
ERR774035
ERR774036
ERR774037
ERR774038
ERR774039
ERR774040
ERR774041
ERR774042
ERR774043
ERR774044
ERR774045
ERR774046
ERR774047
ERR774048
ERR774049
ERR774050
ERR774051
ERR774008
ERR774052
ERR774053
ERR774055
ERR774056
ERR774057
ERR774058
ERR774059
ERR774060
ERR774061
ERR774062
ERR774063
ERR774064
ERR774065
ERR774066
ERR774067
ERR774068
ERR774069
ERR774070
ERR774071

ERR774009
ERR774072
ERR774073
ERR774074
ERR774075
ERR774076
ERR774077
ERR774078
ERR774079
ERR774080
ERR774081
ERR774010
ERR774082
ERR774083
ERR774084
ERR774085
ERR774088
ERR774089
ERR774090
ERR774091
ERR774011
ERR774092
ERR774093
ERR774095
ERR774096
ERR774097
ERR774098
ERR774107
ERR774108
ERR774109
ERR774110
ERR774111
ERR774112
ERR774113
ERR774114
ERR774115
ERR774116
ERR774099
ERR774117
ERR774118
ERR774119
ERR774120
ERR774121
ERR774122
ERR774123
ERR774124
ERR774125
ERR774126
ERR774100
ERR774127

ERR774128
ERR774129
ERR774130
ERR774131
ERR774132
ERR774133
ERR774134
ERR774135
ERR774136
ERR774101
ERR774137
ERR774138
ERR774139
ERR774140
ERR774141
ERR774142
ERR774143
ERR774144
ERR774145
ERR774146
ERR774102
ERR774147
ERR774148
ERR774149
ERR774150
ERR774151
ERR774152
ERR774153
ERR774154
ERR774155
ERR774156
ERR774103
ERR774157
ERR774158
ERR774159
ERR774160
ERR774161
ERR774162
ERR774163
ERR774164
ERR774104
ERR774166
ERR774167
ERR774168
ERR774169
ERR774170
ERR774171
ERR774172
ERR774173
ERR774105

ERR774174
ERR774175
ERR774176
ERR774177
ERR774178
ERR774179
ERR774180
ERR774181
ERR774182
ERR774183
ERR774106
ERR774184
ERR774185
ERR774186
ERR774187
ERR774188
ERR774189
ERR774190
ERR774203
ERR774191
ERR774214
ERR774192
ERR774219
ERR774227
ERR774193
ERR774235
ERR774194
ERR774245
ERR774246
ERR774247
ERR774248
ERR774195
ERR774249
ERR774250
ERR774251
ERR774253
ERR774254
ERR774255
ERR774256
ERR774257
ERR774258
ERR774262
ERR774263
ERR774264
ERR774265
ERR774266
ERR774267
ERR774269
ERR774270
ERR774271

ERR774272
ERR774273
ERR774274
ERR774275
ERR774278
ERR774279
ERR774280
ERR774281
ERR774282
ERR774283
ERR774284
ERR774285
ERR774294
ERR774295
ERR774296
ERR774297
ERR774298
ERR774299
ERR774300
ERR774301
ERR774302
ERR774303
ERR774286
ERR774304
ERR774305
ERR774306
ERR774307
ERR774308
ERR774309
ERR774310
ERR774311
ERR774312
ERR774313
ERR774287
ERR774314
ERR774315
ERR774316
ERR774317
ERR774318
ERR774319
ERR774320
ERR774321
ERR774323
ERR774288
ERR774324
ERR774325
ERR774326
ERR774327
ERR774328
ERR774329

ERR774330
ERR774331
ERR774332
ERR774333
ERR774289
ERR774334
ERR774335
ERR774336
ERR774337
ERR774338
ERR774339
ERR774340
ERR774341
ERR774342
ERR774343
ERR774290
ERR774344
ERR774345
ERR774346
ERR774347
ERR774348
ERR774349
ERR774350
ERR774351
ERR774352
ERR774353
ERR774291
ERR774354
ERR774355
ERR774356
ERR774357
ERR774358
ERR774359
ERR774360
ERR774361
ERR774362
ERR774363
ERR774292
ERR774364
ERR774365
ERR774366
ERR774367
ERR774368
ERR774369
ERR774370
ERR774371
ERR774372
ERR774373
ERR774293
ERR774374

ERR774375
ERR774376
ERR774377
ERR774378
ERR774379
ERR774428
ERR774474
ERR774483
ERR774484
ERR774485
ERR774487
ERR774489
ERR774490
ERR774491
ERR774492
ERR774475
ERR774494
ERR774495
ERR774496
ERR774497
ERR774498
ERR774499
ERR774500
ERR774501
ERR774502
ERR774503
ERR774504
ERR774505
ERR774506
ERR774507
ERR774508
ERR774509
ERR774510
ERR774511
ERR774512
ERR774477
ERR774513
ERR774514
ERR774515
ERR774516
ERR774517
ERR774518
ERR774519
ERR774520
ERR774521
ERR774522
ERR774478
ERR774523
ERR774524
ERR774525

ERR774526
ERR774527
ERR774528
ERR774529
ERR774530
ERR774531
ERR774532
ERR774479
ERR774533
ERR774534
ERR774535
ERR774536
ERR774537
ERR774538
ERR774540
ERR774541
ERR774542
ERR774480
ERR774543
ERR774544
ERR774545
ERR774546
ERR774547
ERR774548
ERR774549
ERR774550
ERR774551
ERR774552
ERR774481
ERR774553
ERR774554
ERR774555
ERR774556
ERR774557
ERR774558
ERR774559
ERR774560
ERR774561
ERR774562
ERR774482
ERR774563
ERR774564
ERR774565
ERR774566
ERR774567
ERR774568
ERR774569
ERR774576
ERR774577
ERR774578

ERR774579
ERR774580
ERR774584
ERR774585
ERR774586
ERR774587
ERR774588
ERR774589
ERR774590
ERR774591
ERR774593
ERR774594
ERR774595
ERR774597
ERR774598
ERR774600
ERR774601
ERR774602
ERR774570
ERR774604
ERR774605
ERR774606
ERR774608
ERR774610
ERR774611
ERR774612
ERR774613
ERR774571
ERR774614
ERR774615
ERR774616
ERR774618
ERR774619
ERR774620
ERR774621
ERR774622
ERR774623
ERR774572
ERR774624
ERR774625
ERR774626
ERR774627
ERR774628
ERR774629
ERR774630
ERR774631
ERR774632
ERR774633
ERR774634
ERR774635

ERR774636
ERR774637
ERR774642
ERR774643
ERR774574
ERR774644
ERR774645
ERR774646
ERR774647
ERR774648
ERR774649
ERR774575
ERR774654
ERR774655
ERR774683
ERR774684
ERR774686
ERR774687
ERR774688
ERR774689
ERR774690
ERR774692
ERR774693
ERR774694
ERR774695
ERR774696
ERR774697
ERR774698
ERR774699
ERR774700
ERR774701
ERR774702
ERR774703
ERR774704
ERR774705
ERR774707
ERR774708
ERR774709
ERR774710
ERR774711
ERR774712
ERR774713
ERR774714
ERR774715
ERR774716
ERR774717
ERR774718
ERR774719
ERR774720
ERR774721

ERR774722
ERR774723
ERR774724
ERR774725
ERR774726
ERR774727
ERR774728
ERR774729
ERR774730
ERR774732
ERR774733
ERR774734
ERR774735
ERR774736
ERR774737
ERR774738
ERR774746
ERR845743
ERR845745
ERR845746
ERR845747
ERR845748
ERR845749
ERR845750
ERR845751
ERR845752
ERR845753
ERR845754
ERR845755
ERR845756
ERR845757
ERR845737
ERR845776
ERR845777
ERR845778
ERR845779
ERR845780
ERR845738
ERR845788
ERR845789
ERR845790
ERR845739
ERR845740
ERR845741
ERR845742
ERR845794
ERR845795
ERR845797
ERR845798
ERR845799

ERR845800
ERR862259
ERR862268
ERR862269
ERR862271
ERR862272
ERR862273
ERR862275
ERR862276
ERR862277
ERR862260
ERR862279
ERR862280
ERR862281
ERR862282
ERR862284
ERR862285
ERR862286
ERR862287
ERR862261
ERR862288
ERR862289
ERR862290
ERR862291
ERR862293
ERR862294
ERR862296
ERR862297
ERR862298
ERR862299
ERR862300
ERR862301
ERR862302
ERR862303
ERR862304
ERR862305
ERR862307
ERR862308
ERR862310
ERR862311
ERR862312
ERR862313
ERR862314
ERR862315
ERR862316
ERR862317
ERR862264
ERR862318
ERR862319
ERR862320

ERR862321
ERR862322
ERR862323
ERR862324
ERR862325
ERR862326
ERR862327
ERR862329
ERR862330
ERR862332
ERR862334
ERR862335
ERR862336
ERR862266
ERR862340
ERR862341
ERR862342
ERR862345
ERR862346
ERR862347
ERR862267
ERR862348
ERR862350
ERR862351
ERR862352
ERR862353
ERR862354
ERR862363
ERR862364
ERR862365
ERR862366
ERR862367
ERR862368
ERR862370
ERR862371
ERR862372
ERR862373
ERR862374
ERR862376
ERR862377
ERR862378
ERR862379
ERR862380
ERR862381
ERR862382
ERR862383
ERR862384
ERR862385
ERR862386
ERR862387

ERR862388
ERR862389
ERR862390
ERR862391
ERR862392
ERR862357
ERR862393
ERR862395
ERR862396
ERR862397
ERR862399
ERR862400
ERR862401
ERR862402
ERR862358
ERR862403
ERR862404
ERR862405
ERR862406
ERR862407
ERR862408
ERR862409
ERR862410
ERR862411
ERR862359
ERR862413
ERR862414
ERR862415
ERR862418
ERR862419
ERR862420
ERR862360
ERR862423
ERR862425
ERR862426
ERR862427
ERR862428
ERR862429
ERR862430
ERR862431
ERR862433
ERR862434
ERR862435
ERR862436
ERR862438
ERR862439
ERR862440
ERR862441
ERR862442
ERR862444

ERR862445
ERR862446
ERR862447
ERR862448
ERR862449
ERR862458
ERR862459
ERR862460
ERR862461
ERR862462
ERR862463
ERR862464
ERR862465
ERR862466
ERR862467
ERR862450
ERR862468
ERR862469
ERR862471
ERR862472
ERR862473
ERR862474
ERR862475
ERR862477
ERR862451
ERR862478
ERR862480
ERR862481
ERR862482
ERR862483
ERR862485
ERR862486
ERR862487
ERR862452
ERR862490
ERR862491
ERR862497
ERR862453
ERR862498
ERR862501
ERR862502
ERR862503
ERR862504
ERR862454
ERR862508
ERR862509
ERR862511
ERR862514
ERR862515
ERR862516

ERR862517
ERR862455
ERR862518
ERR862519
ERR862522
ERR862525
ERR862526
ERR862527
ERR862456
ERR862530
ERR862531
ERR862533
ERR862534
ERR862535
ERR862457
ERR862540
ERR862543
ERR869823
ERR869825
ERR869826
ERR869827
ERR869828
ERR869832
ERR869833
ERR869834
ERR869835
ERR869836
ERR869838
ERR869840
ERR869841
ERR869842
ERR869816
ERR869843
ERR869845
ERR869846
ERR869847
ERR869849
ERR869850
ERR869852
ERR869817
ERR869853
ERR869854
ERR869855
ERR869856
ERR869857
ERR869858
ERR869859
ERR869860
ERR869861
ERR869818

ERR869863
ERR869864
ERR869865
ERR869867
ERR869869
ERR869871
ERR869873
ERR869874
ERR869876
ERR869878
ERR869879
ERR869880
ERR869882
ERR869884
ERR869885
ERR869886
ERR869888
ERR869891
ERR869821
ERR869894
ERR869896
ERR869900
ERR869903
ERR869906
ERR869908
ERR869909
ERR869920
ERR869944
ERR869912
ERR869949
ERR869961
ERR869914
ERR869915
ERR869916
ERR869987
ERR869990
ERR869991
ERR869992
ERR869993
ERR869994
ERR869995
ERR869996
ERR869997
ERR869998
ERR869999
ERR870000
ERR870001
ERR870002
ERR870003
ERR870012

ERR870013
ERR870014
ERR870015
ERR870016
ERR870017
ERR870018
ERR870019
ERR870020
ERR870021
ERR870004
ERR870022
ERR870023
ERR870024
ERR870025
ERR870026
ERR870027
ERR870028
ERR870030
ERR870031
ERR870005
ERR870032
ERR870033
ERR870034
ERR870035
ERR870036
ERR870037
ERR870041
ERR870006
ERR870042
ERR870044
ERR870045
ERR870046
ERR870047
ERR870048
ERR870050
ERR870051
ERR870007
ERR870052
ERR870053
ERR870054
ERR870056
ERR870057
ERR870058
ERR870059
ERR870008
ERR870062
ERR870064
ERR870065
ERR870066
ERR870067

ERR870068
ERR870069
ERR870070
ERR870071
ERR870009
ERR870072
ERR870073
ERR870074
ERR870075
ERR870076
ERR870077
ERR870078
ERR870079
ERR870080
ERR870081
ERR870010
ERR870082
ERR870083
ERR870084
ERR870085
ERR870086
ERR870088
ERR870089
ERR870090
ERR870091
ERR870011
ERR870092
ERR870093
ERR870094
ERR870095
ERR870098
ERR870130
ERR870131
ERR870132
ERR870136
ERR870101
ERR870138
ERR870139
ERR870141
ERR870142
ERR870143
ERR870144
ERR870145
ERR870146
ERR870102
ERR870147
ERR870148
ERR870149
ERR870151
ERR870152

ERR870153
ERR870154
ERR870155
ERR870156
ERR870157
ERR870158
ERR870159
ERR870160
ERR870161
ERR870162
ERR870163
ERR870164
ERR870165
ERR870166
ERR870104
ERR870167
ERR870168
ERR870169
ERR870170
ERR870171
ERR870172
ERR870173
ERR870174
ERR870175
ERR870176
ERR870105
ERR870177
ERR870178
ERR870179
ERR870180
ERR870181
ERR870182
ERR870183
ERR870184
ERR870185
ERR870186
ERR870187
ERR870188
ERR870189
ERR870190
ERR870191
ERR870192
ERR870193
ERR870202
ERR870203
ERR870204
ERR870205
ERR870206
ERR870207
ERR870208

ERR870209
ERR870210
ERR870211
ERR870194
ERR870212
ERR870213
ERR870214
ERR870215
ERR870216
ERR870217
ERR870218
ERR870219
ERR870220
ERR870221
ERR870195
ERR870222
ERR870223
ERR870224
ERR870225
ERR870226
ERR870227
ERR870228
ERR870229
ERR870230
ERR870231
ERR870196
ERR870232
ERR870233
ERR870234
ERR870235
ERR870236
ERR870237
ERR870238
ERR870239
ERR870240
ERR870241
ERR870197
ERR870242
ERR870243
ERR870244
ERR870245
ERR870246
ERR870247
ERR870248
ERR870249
ERR870250
ERR870251
ERR870198
ERR870252
ERR870253

ERR870254
ERR870255
ERR870256
ERR870257
ERR870258
ERR870259
ERR870260
ERR870261
ERR870199
ERR870262
ERR870263
ERR870264
ERR870265
ERR870266
ERR870267
ERR870268
ERR870269
ERR870270
ERR870271
ERR870200
ERR870272
ERR870273
ERR870274
ERR870275
ERR870276
ERR870277
ERR870279
ERR870280
ERR870201
ERR870281
ERR870282
ERR870283
ERR870284
ERR870285
ERR870286
ERR870287
ERR870296
ERR870297
ERR870298
ERR870299
ERR870300
ERR870301
ERR870302
ERR870303
ERR870304
ERR870306
ERR870307
ERR870308
ERR870309
ERR870310

ERR870311
ERR870312
ERR870313
ERR870314
ERR870315
ERR870289
ERR870316
ERR870317
ERR870323
ERR870325
ERR870290
ERR870326
ERR870327
ERR870328
ERR870291
ERR870292
ERR870293
ERR870294
ERR870295
ERR876459
ERR876460
ERR876461
ERR876462
ERR876463
ERR876464
ERR876465
ERR876466
ERR876467
ERR876468
ERR876469
ERR876470
ERR876471
ERR876472
ERR876473
ERR876474
ERR876475
ERR876476
ERR876477
ERR876478
ERR876479
ERR876480
ERR876481
ERR876482
ERR876483
ERR876485
ERR876486
ERR876487
ERR876488
ERR876489
ERR876490

ERR876491
ERR876492
ERR876493
ERR876494
ERR876495
ERR876496
ERR876497
ERR876498
ERR876499
ERR876500
ERR876501
ERR876502
ERR876503
ERR876504
ERR876505
ERR876506
ERR876507
ERR876508
ERR876509
ERR876510
ERR876511
ERR876512
ERR876513
ERR876514
ERR876515
ERR876516
ERR876517
ERR876518
ERR876519
ERR876520
ERR876521
ERR876522
ERR876523
ERR876524
ERR876525
ERR876526
ERR876535
ERR876536
ERR876537
ERR876538
ERR876539
ERR876540
ERR876541
ERR876542
ERR876543
ERR876544
ERR876527
ERR876545
ERR876546
ERR876547

ERR876548
ERR876549
ERR876550
ERR876551
ERR876552
ERR876553
ERR876554
ERR876528
ERR876555
ERR876557
ERR876558
ERR876560
ERR876561
ERR876562
ERR876563
ERR876529
ERR876565
ERR876566
ERR876567
ERR876568
ERR876569
ERR876570
ERR876571
ERR876572
ERR876573
ERR876574
ERR876530
ERR876575
ERR876576
ERR876577
ERR876578
ERR876579
ERR876580
ERR876581
ERR876582
ERR876583
ERR876584
ERR876531
ERR876585
ERR876586
ERR876587
ERR876588
ERR876589
ERR876590
ERR876591
ERR876592
ERR876593
ERR876594
ERR876532
ERR876595

ERR876596
ERR876597
ERR876598
ERR876599
ERR876600
ERR876601
ERR876602
ERR876603
ERR876604
ERR876533
ERR876605
ERR876606
ERR876607
ERR876608
ERR876609
ERR876610
ERR876611
ERR876612
ERR876613
ERR876614
ERR876534
ERR876615
ERR876616
ERR876617
ERR876619
ERR876628
ERR876629
ERR876630
ERR876631
ERR876632
ERR876633
ERR876634
ERR876635
ERR876636
ERR876637
ERR876638
ERR876639
ERR876640
ERR876641
ERR876642
ERR876643
ERR876644
ERR876645
ERR876646
ERR876647
ERR876621
ERR876648
ERR876649
ERR876650
ERR876651

ERR876653
ERR876654
ERR876656
ERR876657
ERR876622
ERR876658
ERR876659
ERR876660
ERR876661
ERR876662
ERR876663
ERR876664
ERR876665
ERR876666
ERR876667
ERR876623
ERR876668
ERR876669
ERR876670
ERR876671
ERR876675
ERR876677
ERR876624
ERR876678
ERR876679
ERR876680
ERR876681
ERR876682
ERR876683
ERR876684
ERR876685
ERR876686
ERR876687
ERR876625
ERR876688
ERR876689
ERR876690
ERR876691
ERR876692
ERR876694
ERR876695
ERR876696
ERR876697
ERR876626
ERR876627
ERR876767
ERR876768
ERR876769
ERR876770
ERR876771

ERR876772
ERR876773
ERR876774
ERR876775
ERR876776
ERR876777
ERR876780
ERR876781
ERR876782
ERR876783
ERR876784
ERR876785
ERR876786
ERR876787
ERR876788
ERR876789
ERR876790
ERR876791
ERR876792
ERR884299
ERR884310
ERR884312
ERR884313
ERR884314
ERR884315
ERR884316
ERR884317
ERR884300
ERR884318
ERR884319
ERR884321
ERR884322
ERR884323
ERR884324
ERR884326
ERR884327
ERR884301
ERR884328
ERR884329
ERR884330
ERR884331
ERR884332
ERR884333
ERR884334
ERR884335
ERR884336
ERR884337
ERR884302
ERR884338
ERR884339

ERR884340
ERR884341
ERR884342
ERR884343
ERR884344
ERR884345
ERR884346
ERR884347
ERR884303
ERR884348
ERR884349
ERR884350
ERR884351
ERR884352
ERR884353
ERR884354
ERR884355
ERR884356
ERR884357
ERR884304
ERR884358
ERR884359
ERR884360
ERR884361
ERR884362
ERR884363
ERR884364
ERR884365
ERR884366
ERR884367
ERR884305
ERR884368
ERR884369
ERR884370
ERR884371
ERR884372
ERR884373
ERR884374
ERR884375
ERR884376
ERR884377
ERR884306
ERR884378
ERR884379
ERR884380
ERR884381
ERR884382
ERR884383
ERR884384
ERR884385

ERR884386
ERR884387
ERR884307
ERR884388
ERR884389
ERR884390
ERR884391
ERR884392
ERR884393
ERR884394
ERR884404
ERR884405
ERR884406
ERR884407
ERR884408
ERR884409
ERR884410
ERR884411
ERR884412
ERR884395
ERR884413
ERR884414
ERR884415
ERR884416
ERR884417
ERR884418
ERR884419
ERR884420
ERR884421
ERR884422
ERR884396
ERR884423
ERR884424
ERR884425
ERR884426
ERR884427
ERR884428
ERR884429
ERR884430
ERR884431
ERR884432
ERR884397
ERR884433
ERR884434
ERR884435
ERR884436
ERR884437
ERR884438
ERR884439
ERR884440

ERR884441
ERR884442
ERR884398
ERR884445
ERR884446
ERR884447
ERR884448
ERR884449
ERR884450
ERR884451
ERR884452
ERR884399
ERR884453
ERR884454
ERR884455
ERR884456
ERR884457
ERR884458
ERR884459
ERR884460
ERR884461
ERR884462
ERR884400
ERR884463
ERR884464
ERR884465
ERR884467
ERR884468
ERR884469
ERR884470
ERR884471
ERR884472
ERR884401
ERR884402
ERR900646
ERR900655
ERR900656
ERR900657
ERR900658
ERR900659
ERR900661
ERR900662
ERR900663
ERR900664
ERR900647
ERR900665
ERR900666
ERR900667
ERR900668
ERR900669

ERR900670
ERR900671
ERR900672
ERR900673
ERR900674
ERR900648
ERR900675
ERR900676
ERR900677
ERR900678
ERR900680
ERR900681
ERR900682
ERR900683
ERR900684
ERR900649
ERR900685
ERR900686
ERR900687
ERR900688
ERR900689
ERR900690
ERR900691
ERR900692
ERR900693
ERR900694
ERR900650
ERR900695
ERR900696
ERR900697
ERR900698
ERR900699
ERR900700
ERR900701
ERR900702
ERR900703
ERR900704
ERR900651
ERR900705
ERR900706
ERR900707
ERR900708
ERR900709
ERR900710
ERR900711
ERR900712
ERR900713
ERR900714
ERR900652
ERR900715

ERR900716
ERR900717
ERR900718
ERR900719
ERR900721
ERR900722
ERR900723
ERR900653
ERR900725
ERR900726
ERR900727
ERR900728
ERR900729
ERR900730
ERR900731
ERR900732
ERR900733
ERR900734
ERR900735
ERR900736
ERR900737
ERR900738
ERR900739
ERR908850
ERR908859
ERR908860
ERR908861
ERR908863
ERR908865
ERR908866
ERR908867
ERR908868
ERR908851
ERR908869
ERR908873
ERR908874
ERR908875
ERR908876
ERR908852
ERR908879
ERR908880
ERR908882
ERR908883
ERR908884
ERR908885
ERR908887
ERR908888
ERR908853
ERR908889
ERR908890

ERR908891
ERR908892
ERR908893
ERR908894
ERR908896
ERR908897
ERR908898
ERR908899
ERR908900
ERR908901
ERR908902
ERR908905
ERR908907
ERR908908
ERR908855
ERR908909
ERR908910
ERR908911
ERR908912
ERR908913
ERR908914
ERR908915
ERR908916
ERR908917
ERR908918
ERR908919
ERR908920
ERR908921
ERR908922
ERR908923
ERR908924
ERR908925
ERR908926
ERR908927
ERR908928
ERR908857
ERR908929
ERR908930
ERR908931
ERR908932
ERR908933
ERR908934
ERR908935
ERR908937
ERR908938
ERR908858
ERR908940
ERR908941
ERR908942
ERR908943

ERR908944
ERR908954
ERR908955
ERR908956
ERR908957
ERR908958
ERR908959
ERR908961
ERR908962
ERR908963
ERR908946
ERR908964
ERR908965
ERR908966
ERR908967
ERR908968
ERR908970
ERR908971
ERR908972
ERR908947
ERR908974
ERR908975
ERR908976
ERR908977
ERR908979
ERR908980
ERR908981
ERR908982
ERR908983
ERR908948
ERR908984
ERR908986
ERR908987
ERR908988
ERR908989
ERR908990
ERR908991
ERR908992
ERR908993
ERR908949
ERR908994
ERR908995
ERR908996
ERR908997
ERR908998
ERR908999
ERR909000
ERR909001
ERR909002
ERR909003

ERR908950
ERR909004
ERR909005
ERR909008
ERR909009
ERR909010
ERR909011
ERR909012
ERR909013
ERR908951
ERR909014
ERR909015
ERR909017
ERR909019
ERR909020
ERR909021
ERR909022
ERR909023
ERR908952
ERR909024
ERR909025
ERR909026
ERR909027
ERR909028
ERR909029
ERR909030
ERR909031
ERR909032
ERR909033
ERR908953
ERR909034
ERR909035
ERR909036
ERR909037
ERR909038
ERR909039
ERR909049
ERR909050
ERR909051
ERR909052
ERR909053
ERR909054
ERR909055
ERR909057
ERR909041
ERR909059
ERR909060
ERR909061
ERR909062
ERR909063

ERR909065
ERR909066
ERR909067
ERR909068
ERR909042
ERR909069
ERR909070
ERR909071
ERR909072
ERR909073
ERR909074
ERR909075
ERR909077
ERR909078
ERR909080
ERR909081
ERR909082
ERR909083
ERR909084
ERR909085
ERR909086
ERR909087
ERR909088
ERR909044
ERR909089
ERR909090
ERR909091
ERR909092
ERR909093
ERR909094
ERR909095
ERR909096
ERR909098
ERR909045
ERR909099
ERR909100
ERR909102
ERR909103
ERR909104
ERR909105
ERR909106
ERR909107
ERR909108
ERR909046
ERR909109
ERR909110
ERR909111
ERR909112
ERR909113
ERR909114

ERR909115
ERR909116
ERR909117
ERR909118
ERR909047
ERR909119
ERR909120
ERR909122
ERR909123
ERR909124
ERR909126
ERR909127
ERR909128
ERR909048
ERR909129
ERR909130
ERR909131
ERR909132
ERR909133
ERR909134
ERR909135
ERR909144
ERR909145
ERR909147
ERR909148
ERR909149
ERR909150
ERR909151
ERR909152
ERR909153
ERR909136
ERR909154
ERR909155
ERR909156
ERR909157
ERR909158
ERR909159
ERR909160
ERR909161
ERR909162
ERR909163
ERR909137
ERR909164
ERR909165
ERR909166
ERR909167
ERR909169
ERR909171
ERR909172
ERR909138

ERR909174
ERR909175
ERR909176
ERR909177
ERR909179
ERR909180
ERR909181
ERR909182
ERR909183
ERR909139
ERR909184
ERR909185
ERR909186
ERR909187
ERR909188
ERR909189
ERR909190
ERR909191
ERR909192
ERR909193
ERR909140
ERR909194
ERR909195
ERR909196
ERR909197
ERR909198
ERR909199
ERR909200
ERR909201
ERR909202
ERR909203
ERR909141
ERR909204
ERR909205
ERR909206
ERR909208
ERR909209
ERR909212
ERR909213
ERR909215
ERR909216
ERR909218
ERR909220
ERR909221
ERR909222
ERR909223
ERR909224
ERR909226
ERR909227
ERR909228

ERR909229
ERR909230
ERR909239
ERR909241
ERR909242
ERR909243
ERR909244
ERR909245
ERR909246
ERR909247
ERR909248
ERR909231
ERR909249
ERR909250
ERR909251
ERR909252
ERR909253
ERR909255
ERR909256
ERR909257
ERR909258
ERR909259
ERR909260
ERR909261
ERR909262
ERR909263
ERR909265
ERR909266
ERR909267
ERR909268
ERR909233
ERR909269
ERR909271
ERR909274
ERR909275
ERR909278
ERR909234
ERR909279
ERR909280
ERR909281
ERR909282
ERR909283
ERR909284
ERR909285
ERR909286
ERR909287
ERR909235
ERR909290
ERR909291
ERR909293

ERR909294
ERR909295
ERR909297
ERR909236
ERR909299
ERR909300
ERR909302
ERR909303
ERR909304
ERR909305
ERR909306
ERR909308
ERR909237
ERR909309
ERR909310
ERR909311
ERR909312
ERR909313
ERR909316
ERR909317
ERR909318
ERR909238
ERR909319
ERR909320
ERR909321
ERR909323
ERR909324
ERR909325
ERR909328
ERR909333
ERR913059
ERR913068
ERR913069
ERR913070
ERR913071
ERR913072
ERR913073
ERR913075
ERR913076
ERR913077
ERR913060
ERR913078
ERR913079
ERR913080
ERR913081
ERR913083
ERR913084
ERR913085
ERR913086
ERR913087

ERR913061
ERR913088
ERR913089
ERR913090
ERR913091
ERR913092
ERR913093
ERR913094
ERR913096
ERR913062
ERR913097
ERR913099
ERR913100
ERR913101
ERR913103
ERR913104
ERR913105
ERR913063
ERR913107
ERR913108
ERR913109
ERR913110
ERR913111
ERR913112
ERR913115
ERR913116
ERR913064
ERR913117
ERR913118
ERR913119
ERR913120
ERR913121
ERR913122
ERR913123
ERR913124
ERR913125
ERR913126
ERR913065
ERR913128
ERR913129
ERR913130
ERR913131
ERR913132
ERR913133
ERR913135
ERR913136
ERR913066
ERR913137
ERR913138
ERR913139

ERR913140
ERR913141
ERR913142
ERR913144
ERR913145
ERR913146
ERR913147
ERR913148
ERR913149
ERR913150
ERR913151
ERR913152
ERR913153
ERR913162
ERR913163
ERR913164
ERR913165
ERR913166
ERR913167
ERR913168
ERR913169
ERR913170
ERR913171
ERR913154
ERR913172
ERR913173
ERR913174
ERR913175
ERR913176
ERR913177
ERR913178
ERR913179
ERR913180
ERR913181
ERR913182
ERR913183
ERR913184
ERR913185
ERR913186
ERR913187
ERR913188
ERR913189
ERR913190
ERR913191
ERR913156
ERR913193
ERR913194
ERR913195
ERR913196
ERR913197

ERR913198
ERR913199
ERR913200
ERR913201
ERR913157
ERR913202
ERR913203
ERR913204
ERR913205
ERR913206
ERR913207
ERR913158
ERR913214
ERR913159
ERR913228
ERR913229
ERR913230
ERR913231
ERR913160
ERR913232
ERR913233
ERR913234
ERR913161
ERR913235
ERR913244
ERR913245
ERR913248
ERR913249
ERR913251
ERR913252
ERR913253
ERR913236
ERR913254
ERR913255
ERR913256
ERR913259
ERR913260
ERR913261
ERR913262
ERR913263
ERR913237
ERR913265
ERR913267
ERR913268
ERR913269
ERR913270
ERR913273
ERR913238
ERR913274
ERR913275

ERR913276
ERR913277
ERR913278
ERR913279
ERR913280
ERR913281
ERR913282
ERR913283
ERR913284
ERR913285
ERR913287
ERR913290
ERR913291
ERR913292
ERR913293
ERR913240
ERR913294
ERR913295
ERR913297
ERR913298
ERR913299
ERR913300
ERR913301
ERR913302
ERR913241
ERR913304
ERR913305
ERR913306
ERR913309
ERR913310
ERR913313
ERR913242
ERR913315
ERR913316
ERR913317
ERR913318
ERR913319
ERR913320
ERR913321
ERR913376
ERR913377
ERR913378
ERR913379
ERR913380
ERR913381
ERR913382
ERR913383
ERR913384
ERR913385
ERR913386

ERR913387
ERR913389
ERR913390
ERR913391
ERR913392
ERR913393
ERR913394
ERR913395
ERR913396
ERR913406
ERR913408
ERR913409
ERR913410
ERR913411
ERR913412
ERR913415
ERR913416
ERR913418
ERR913420
ERR913422
ERR913423
ERR913426
ERR913429
ERR913431
ERR913400
ERR913401
ERR913402
ERR913464
ERR913465
ERR913403
ERR913466
ERR913467
ERR913468
ERR913469
ERR913470
ERR913471
ERR913473
ERR913474
ERR913475
ERR913404
ERR913476
ERR913477
ERR913478
ERR913479
ERR913480
ERR913481
ERR913482
ERR913405
ERR913483
ERR913493

ERR913494
ERR913496
ERR913498
ERR913499
ERR913500
ERR913484
ERR913502
ERR913503
ERR913504
ERR913505
ERR913506
ERR913507
ERR913508
ERR913509
ERR913485
ERR913510
ERR913511
ERR913512
ERR913514
ERR913519
ERR913520
ERR913521
ERR913522
ERR913523
ERR913524
ERR913525
ERR913526
ERR913527
ERR913528
ERR913487
ERR913529
ERR913530
ERR913533
ERR913534
ERR913535
ERR913536
ERR913537
ERR913539
ERR913540
ERR913541
ERR913542
ERR913544
ERR913489
ERR913546
ERR913547
ERR913548
ERR913551
ERR913552
ERR913554
ERR913555

ERR913490
ERR913556
ERR913557
ERR913558
ERR913560
ERR913561
ERR913563
ERR913564
ERR913491
ERR913565
ERR913566
ERR913568
ERR913569
ERR925027
ERR925036
ERR925126
ERR925127
ERR925128
ERR925129
ERR925130
ERR925131
ERR925132
ERR925133
ERR925037
ERR925134
ERR925135
ERR925136
ERR925137
ERR925138
ERR925139
ERR925140
ERR925141
ERR925142
ERR925143
ERR925038
ERR925144
ERR925145
ERR925147
ERR925148
ERR925149
ERR925150
ERR925152
ERR925153
ERR925154
ERR925155
ERR925156
ERR925157
ERR925159
ERR925160
ERR925162

ERR925163
ERR925040
ERR925164
ERR925165
ERR925166
ERR925167
ERR925168
ERR925170
ERR925172
ERR925173
ERR925174
ERR925175
ERR925176
ERR925177
ERR925178
ERR925179
ERR925181
ERR925182
ERR925183
ERR925186
ERR925187
ERR925188
ERR925189
ERR925190
ERR925191
ERR925043
ERR925192
ERR925193
ERR925194
ERR925197
ERR925198
ERR925199
ERR925200
ERR925044
ERR925201
ERR925202
ERR925204
ERR925205
ERR925207
ERR925208
ERR925209
ERR925045
ERR925211
ERR925028
ERR925046
ERR925048
ERR925049
ERR925050
ERR925051
ERR925052

ERR925053
ERR925054
ERR925055
ERR925029
ERR925056
ERR925057
ERR925058
ERR925059
ERR925061
ERR925062
ERR925063
ERR925064
ERR925065
ERR925030
ERR925066
ERR925067
ERR925068
ERR925069
ERR925070
ERR925071
ERR925072
ERR925073
ERR925074
ERR925075
ERR925031
ERR925076
ERR925077
ERR925078
ERR925079
ERR925080
ERR925081
ERR925083
ERR925084
ERR925085
ERR925032
ERR925086
ERR925087
ERR925088
ERR925089
ERR925090
ERR925091
ERR925092
ERR925093
ERR925094
ERR925033
ERR925095
ERR925097
ERR925098
ERR925099
ERR925100

ERR925101
ERR925102
ERR925103
ERR925105
ERR925106
ERR925107
ERR925109
ERR925110
ERR925111
ERR925112
ERR925114
ERR925035
ERR925115
ERR925116
ERR925117
ERR925119
ERR925120
ERR925122
ERR925123
ERR925212
ERR925221
ERR925312
ERR925313
ERR925314
ERR925315
ERR925316
ERR925222
ERR925321
ERR925322
ERR925323
ERR925327
ERR925328
ERR925329
ERR925330
ERR925331
ERR925332
ERR925333
ERR925334
ERR925335
ERR925336
ERR925337
ERR925338
ERR925339
ERR925224
ERR925340
ERR925341
ERR925342
ERR925343
ERR925344
ERR925345

ERR925346
ERR925347
ERR925348
ERR925225
ERR925351
ERR925353
ERR925355
ERR925356
ERR925357
ERR925358
ERR925359
ERR925226
ERR925360
ERR925363
ERR925364
ERR925366
ERR925368
ERR925369
ERR925227
ERR925370
ERR925371
ERR925372
ERR925373
ERR925374
ERR925375
ERR925376
ERR925377
ERR925378
ERR925228
ERR925380
ERR925381
ERR925382
ERR925383
ERR925384
ERR925385
ERR925386
ERR925387
ERR925389
ERR925229
ERR925391
ERR925393
ERR925395
ERR925396
ERR925397
ERR925398
ERR925399
ERR925230
ERR925400
ERR925401
ERR925213

ERR925231
ERR925232
ERR925234
ERR925236
ERR925237
ERR925238
ERR925240
ERR925214
ERR925241
ERR925242
ERR925243
ERR925244
ERR925245
ERR925247
ERR925248
ERR925249
ERR925215
ERR925251
ERR925252
ERR925253
ERR925255
ERR925256
ERR925257
ERR925258
ERR925259
ERR925260
ERR925216
ERR925261
ERR925262
ERR925265
ERR925266
ERR925268
ERR925269
ERR925270
ERR925272
ERR925273
ERR925274
ERR925275
ERR925277
ERR925278
ERR925279
ERR925280
ERR925218
ERR925281
ERR925282
ERR925283
ERR925284
ERR925285
ERR925286
ERR925287

ERR925288
ERR925289
ERR925290
ERR925219
ERR925291
ERR925293
ERR925294
ERR925295
ERR925296
ERR925297
ERR925298
ERR925300
ERR925220
ERR925301
ERR925303
ERR925305
ERR925307
ERR925308
ERR925309
ERR956278
ERR956280
ERR956281
ERR956282
ERR956283
ERR956284
ERR956285
ERR956287
ERR956288
ERR956289
ERR956290
ERR956293
ERR956295
ERR956297
ERR956298
ERR956299
ERR956301
ERR956302
ERR956303
ERR956304
ERR956305
ERR956306
ERR956307
ERR956310
ERR956312
ERR956313
ERR979874
ERR979884
ERR979885
ERR979886
ERR979887

ERR979888
ERR979889
ERR979890
ERR979891
ERR979892
ERR979875
ERR979893
ERR979894
ERR979895
ERR979896
ERR979897
ERR979898
ERR979899
ERR979901
ERR979902
ERR979903
ERR979904
ERR979905
ERR979906
ERR979907
ERR979908
ERR979909
ERR979910
ERR979911
ERR979912
ERR979877
ERR979913
ERR979916
ERR979917
ERR979918
ERR979919
ERR979920
ERR979921
ERR979922
ERR979878
ERR979923
ERR979924
ERR979925
ERR979926
ERR979927
ERR979928
ERR979929
ERR979931
ERR979932
ERR979879
ERR979933
ERR979934
ERR979935
ERR979936
ERR979937

ERR979938
ERR979940
ERR979941
ERR979942
ERR979880
ERR979943
ERR979944
ERR979945
ERR979946
ERR979947
ERR979948
ERR979949
ERR979950
ERR979951
ERR979952
ERR979881
ERR979953
ERR979954
ERR979956
ERR979957
ERR979966
ERR979968
ERR979969
ERR979970
ERR979973
ERR979975
ERR979958
ERR979976
ERR979977
ERR979980
ERR979981
ERR979982
ERR979983
ERR979984
ERR979985
ERR979959
ERR979986
ERR979987
ERR979988
ERR979989
ERR979990
ERR979991
ERR979992
ERR979993
ERR979994
ERR979995
ERR979960
ERR979996
ERR979997
ERR979998

ERR979999
ERR980000
ERR980001
ERR980002
ERR980003
ERR980004
ERR980005
ERR980006
ERR980007
ERR980008
ERR980009
ERR980010
ERR980011
ERR980012
ERR980013
ERR980014
ERR980015
ERR979962
ERR980017
ERR980020
ERR980021
ERR980022
ERR980023
ERR980025
ERR979963
ERR980026
ERR980027
ERR980028
ERR980029
ERR980030
ERR980031
ERR980032
ERR980033
ERR980034
ERR980035
ERR979964
ERR980036
ERR980037
ERR980038
ERR980039
ERR979965
ERR980040
ERR980049
ERR980050
ERR980052
ERR980053
ERR980054
ERR980057
ERR980058
ERR980059

ERR980060
ERR980061
ERR980062
ERR980063
ERR980064
ERR980065
ERR980066
ERR980067
ERR980068
ERR980042
ERR980069
ERR980070
ERR980071
ERR980072
ERR980073
ERR980074
ERR980075
ERR980076
ERR980077
ERR980043
ERR980078
ERR980079
ERR980080
ERR980081
ERR980082
ERR980083
ERR980084
ERR980085
ERR980086
ERR980087
ERR980044
ERR980088
ERR980089
ERR980090
ERR980091
ERR980092
ERR980093
ERR980094
ERR980095
ERR980096
ERR980097
ERR980045
ERR980098
ERR980100
ERR980101
ERR980103
ERR980104
ERR980105
ERR980106
ERR980107

ERR980046
ERR980108
ERR980109
ERR980111
ERR980112
ERR980113
ERR980114
ERR980115
ERR980116
ERR980117
ERR980047
ERR980118
ERR980119
ERR980120
ERR980121
ERR980048
ERR980132
ERR980133
ERR980134
ERR980135
ERR980136
ERR980123
ERR980141
ERR980143
ERR980144
ERR980149
ERR980150
ERR980124
ERR980151
ERR980153
ERR980161
ERR980164
ERR980165
ERR980166
ERR980168
ERR980169
ERR980170
ERR980126
ERR980172
ERR980173
ERR980176
ERR980180
ERR980181
ERR980182
ERR980183
ERR980184
ERR980186
ERR980187
ERR980188
ERR980189

ERR980128
ERR980191
ERR980192
ERR980194
ERR980129
ERR980198
ERR980201
ERR980130
ERR980202
ERR980210
ERR980212
ERR980213
ERR980214
ERR980215
ERR980216
ERR980217
ERR980203
ERR980218
ERR980219
ERR980220
ERR980221
ERR980222
ERR980223
ERR980204
ERR980232
ERR980233
ERR980234
ERR980205
ERR980236
ERR980239
ERR980241
ERR980242
ERR980244
ERR980247
ERR980248
ERR980249
ERR980250
ERR980251
ERR980207
ERR980252
ERR980253
ERR980254
ERR980257
ERR980258
ERR980259
ERR980260
ERR980208
ERR980261
ERR980262
ERR980263

ERR980264
ERR980265
ERR980267
ERR980269
ERR980209
ERR980270
ERR980271
ERR980272
ERR980273
ERR980283
ERR980284
ERR980286
ERR980287
ERR980288
ERR980289
ERR980291
ERR980292
ERR980275
ERR980293
ERR980294
ERR980295
ERR980296
ERR980298
ERR980300
ERR980301
ERR980302
ERR980276
ERR980303
ERR980304
ERR980305
ERR980306
ERR980307
ERR980309
ERR980310
ERR980311
ERR980312
ERR980277
ERR980313
ERR980315
ERR980317
ERR980318
ERR980319
ERR980320
ERR980321
ERR980278
ERR980323
ERR980326
ERR980327
ERR980328
ERR980329

ERR980330
ERR980331
ERR980332
ERR980279
ERR980333
ERR980334
ERR980335
ERR980336
ERR980337
ERR980280
ERR980340
ERR980342
ERR980343
ERR980344
ERR980345
ERR980346
ERR980347
ERR980281
ERR980349
ERR980350
ERR980351
ERR980353
ERR980355
ERR980356
ERR980282
ERR997462
ERR997471
ERR997473
ERR997474
ERR997475
ERR997476
ERR997477
ERR997480
ERR997463
ERR997481
ERR997482
ERR997483
ERR997484
ERR997485
ERR997486
ERR997487
ERR997488
ERR997489
ERR997490
ERR997464
ERR997491
ERR997492
ERR997493
ERR997494
ERR997495

ERR997496
ERR997497
ERR997498
ERR997499
ERR997500
ERR997465
ERR997501
ERR997502
ERR997504
ERR997506
ERR997507
ERR997508
ERR997509
ERR997510
ERR997511
ERR997512
ERR997513
ERR997514
ERR997515
ERR997516
ERR997517
ERR997518
ERR997519
ERR997467
ERR997521
ERR997522
ERR997523
ERR997524
ERR997525
ERR997526
ERR997527
ERR997528
ERR997529
ERR997530
ERR997468
ERR997531
ERR997532
ERR997533
ERR997534
ERR997535
ERR997536
ERR997537
ERR997538
ERR997539
ERR997540
ERR997469
ERR997541
ERR997542
ERR997551
ERR997552

ERR997553
ERR997554
ERR997555
ERR997556
ERR997557
ERR997558
ERR997559
ERR997560
ERR997543
ERR997561
ERR997562
ERR997563
ERR997564
ERR997565
ERR997566
ERR997567
ERR997568
ERR997569
ERR997570
ERR997544
ERR997571
ERR997572
ERR997573
ERR997574
ERR997575
ERR997576
ERR997577
ERR997578
ERR997579
ERR997580
ERR997545
ERR997581
ERR997582
ERR997583
ERR997584
ERR997585
ERR997587
ERR997588
ERR997589
ERR997590
ERR997546
ERR997591
ERR997592
ERR997593
ERR997594
ERR997595
ERR997596
ERR997597
ERR997598
ERR997599

ERR997600
ERR997547
ERR997601
ERR997602
ERR997603
ERR997604
ERR997605
ERR997606
ERR997607
ERR997608
ERR997609
ERR997610
ERR997548
ERR997611
ERR997614
ERR997615
ERR997616
ERR997617
ERR997618
ERR997549
ERR997621
ERR997550
ERR997622
ERR997631
ERR997632
ERR997633
ERR997635
ERR997637
ERR997638
ERR997639
ERR997640
ERR997641
ERR997642
ERR997643
ERR997644
ERR997645
ERR997646
ERR997647
ERR997648
ERR997649
ERR997650
ERR997624
ERR997652
ERR997654
ERR997655
ERR997656
ERR997657
ERR997658
ERR997659
ERR997660

ERR997625
ERR997661
ERR997662
ERR997663
ERR997664
ERR997665
ERR997666
ERR997667
ERR997668
ERR997669
ERR997670
ERR997626
ERR997671
ERR997672
ERR997673
ERR997674
ERR997675
ERR997676
ERR997677
ERR997678
ERR997679
ERR997680
ERR997627
ERR997681
ERR997682
ERR997683
ERR997684
ERR997685
ERR997686
ERR997687
ERR997688
ERR997689
ERR997690
ERR997628
ERR997691
ERR997692
ERR997693
ERR997694
ERR997696
ERR997697
ERR997698
ERR997699
ERR997700
ERR997629
ERR997701
ERR997630
ERR984238
ERR984247
ERR984248
ERR984249

ERR984250
ERR984251
ERR984252
ERR984253
ERR984254
ERR984255
ERR984256
ERR984239
ERR984257
ERR984258
ERR984259
ERR984261
ERR984262
ERR984263
ERR984264
ERR984265
ERR984266
ERR984240
ERR984267
ERR984268
ERR984269
ERR984270
ERR984271
ERR984272
ERR984273
ERR984274
ERR984275
ERR984276
ERR984241
ERR984277
ERR984278
ERR984279
ERR984280
ERR984281
ERR984282
ERR984283
ERR984284
ERR984285
ERR984286
ERR984242
ERR984287
ERR984288
ERR984289
ERR984290
ERR984291
ERR984292
ERR984293
ERR984294
ERR984295
ERR984296

ERR984243
ERR984297
ERR984298
ERR984244
ERR984245
ERR984246
ERR1065121
ERR1065122
ERR1065123
ERR1065125
ERR1065126
ERR1065127
ERR1065130
ERR1065113
ERR1065131
ERR1065133
ERR1065134
ERR1065135
ERR1065138
ERR1065139
ERR1065140
ERR1065141
ERR1065142
ERR1065143
ERR1065144
ERR1065146
ERR1065147
ERR1065148
ERR1065150
ERR1065115
ERR1065151
ERR1065154
ERR1065155
ERR1065156
ERR1065157
ERR1065158
ERR1065159
ERR1065160
ERR1065116
ERR1065161
ERR1065162
ERR1065163
ERR1065164
ERR1065165
ERR1065166
ERR1065167
ERR1065168
ERR1065170
ERR1065117
ERR1065172

ERR1065173
ERR1065174
ERR1065175
ERR1065176
ERR1065177
ERR1065178
ERR1065180
ERR1065118
ERR1065182
ERR1065183
ERR1065185
ERR1065187
ERR1065188
ERR1065189
ERR1065190
ERR1065119
ERR1065191
ERR1065193
ERR1065194
ERR1065195
ERR1065196
ERR1065197
ERR1065199
ERR1065201
ERR1065202
ERR1065203
ERR1065204
ERR1065205
ERR1065206
ERR1065215
ERR1065216
ERR1065217
ERR1065218
ERR1065219
ERR1065221
ERR1065222
ERR1065207
ERR1065226
ERR1065228
ERR1065229
ERR1065230
ERR1065232
ERR1065233
ERR1065235
ERR1065236
ERR1065238
ERR1065239
ERR1065241
ERR1065242
ERR1065243

ERR1065209
ERR1065244
ERR1065246
ERR1065248
ERR1065250
ERR1065251
ERR1065252
ERR1065210
ERR1065254
ERR1065255
ERR1065256
ERR1065258
ERR1065259
ERR1065260
ERR1065262
ERR1065211
ERR1065264
ERR1065265
ERR1065266
ERR1065267
ERR1065268
ERR1065269
ERR1065270
ERR1065271
ERR1065272
ERR1065273
ERR1065212
ERR1065274
ERR1065275
ERR1065277
ERR1065278
ERR1065279
ERR1065280
ERR1065213
ERR1065282
ERR1065283
ERR1065284
ERR1065285
ERR1065286
ERR1065287
ERR1065288
ERR1065289
ERR1065290
ERR1065291
ERR1065214
ERR1065292
ERR1065294
ERR1065295
ERR1065296
ERR1065297

ERR1065306
ERR1065307
ERR1065308
ERR1065309
ERR1065310
ERR1065311
ERR1065313
ERR1065315
ERR1065298
ERR1065316
ERR1065317
ERR1065318
ERR1065319
ERR1065320
ERR1065322
ERR1065323
ERR1065324
ERR1065325
ERR1065299
ERR1065326
ERR1065328
ERR1065329
ERR1065331
ERR1065332
ERR1065333
ERR1065334
ERR1065335
ERR1065300
ERR1065336
ERR1065337
ERR1065338
ERR1065339
ERR1065340
ERR1065341
ERR1065342
ERR1065343
ERR1065301
ERR1065347
ERR1065348
ERR1065349
ERR1065350
ERR1065351
ERR1065352
ERR1065353
ERR1065355
ERR1065302
ERR1065356
ERR1065357
ERR1065358
ERR1065359

ERR1065361
ERR1065362
ERR1065363
ERR1065364
ERR1065365
ERR1065303
ERR1065366
ERR1065367
ERR1065369
ERR1065370
ERR1065371
ERR1065372
ERR1065374
ERR1065375
ERR1065304
ERR1065376
ERR1065377
ERR1065378
ERR1065379
ERR1065380
ERR1065382
ERR1065383
ERR1065384
ERR1065385
ERR1065305
ERR1065386
ERR1065387
ERR1065388
ERR1065389
ERR1065390
ERR1065391
ERR1065400
ERR1065401
ERR1065402
ERR1065403
ERR1065404
ERR1065405
ERR1065406
ERR1065407
ERR1065408
ERR1065409
ERR1065392
ERR1065410
ERR1065411
ERR1065412
ERR1065414
ERR1065415
ERR1065416
ERR1065417
ERR1065419

ERR1065393
ERR1065421
ERR1065422
ERR1065424
ERR1065425
ERR1065427
ERR1065428
ERR1065429
ERR1065394
ERR1065430
ERR1065431
ERR1065432
ERR1065433
ERR1065435
ERR1065437
ERR1065438
ERR1065395
ERR1065440
ERR1065441
ERR1065443
ERR1065445
ERR1065446
ERR1065447
ERR1065449
ERR1065396
ERR1065451
ERR1065453
ERR1065454
ERR1065455
ERR1065456
ERR1065457
ERR1065458
ERR1065459
ERR1065397
ERR1065461
ERR1065462
ERR1065465
ERR1065467
ERR1065469
ERR1065398
ERR1065470
ERR1065471
ERR1065472
ERR1065475
ERR1065477
ERR1065479
ERR1065480
ERR1065481
ERR1065482
ERR1065483

ERR1065493
ERR1065495
ERR1065496
ERR1065498
ERR1065499
ERR1065501
ERR1065502
ERR1065485
ERR1065503
ERR1065504
ERR1065505
ERR1065506
ERR1065507
ERR1065510
ERR1065511
ERR1065512
ERR1065513
ERR1065514
ERR1065515
ERR1065518
ERR1065519
ERR1065521
ERR1065522
ERR1065487
ERR1065523
ERR1065524
ERR1065525
ERR1065526
ERR1065527
ERR1065528
ERR1065529
ERR1065530
ERR1065531
ERR1065532
ERR1065488
ERR1065533
ERR1065534
ERR1065535
ERR1065536
ERR1065537
ERR1065538
ERR1065539
ERR1065540
ERR1065542
ERR1065489
ERR1065544
ERR1065545
ERR1065547
ERR1065548
ERR1065550

ERR1065551
ERR1065552
ERR1065490
ERR1065553
ERR1065554
ERR1065555
ERR1065556
ERR1065557
ERR1065558
ERR1065559
ERR1065560
ERR1065561
ERR1065562
ERR1065491
ERR1065563
ERR1065564
ERR1065565
ERR1065567
ERR1065568
ERR1065570
ERR1065571
ERR1065572
ERR1065573
ERR1065574
ERR1065585
ERR1065586
ERR1065587
ERR1065589
ERR1065590
ERR1065591
ERR1065595
ERR1065596
ERR1065598
ERR1065599
ERR1065601
ERR1065602
ERR1065605
ERR1065607
ERR1065608
ERR1065609
ERR1065610
ERR1065611
ERR1065612
ERR1065578
ERR1065614
ERR1065616
ERR1065617
ERR1065618
ERR1065620
ERR1065622

ERR1065623
ERR1065624
ERR1065626
ERR1065627
ERR1065629
ERR1065631
ERR1065632
ERR1065633
ERR1065580
ERR1065635
ERR1065636
ERR1065638
ERR1065639
ERR1065583
ERR1065640
ERR1065649
ERR1065650
ERR1065652
ERR1065653
ERR1065654
ERR1065655
ERR1065656
ERR1065657
ERR1065641
ERR1065659
ERR1065660
ERR1065661
ERR1065662
ERR1065663
ERR1065664
ERR1065665
ERR1065666
ERR1065667
ERR1065668
ERR1065642
ERR1065669
ERR1065670
ERR1065671
ERR1065672
ERR1065673
ERR1065674
ERR1065675
ERR1065676
ERR1065677
ERR1065678
ERR1065643
ERR1065679
ERR1065680
ERR1065682
ERR1065683

ERR1065684
ERR1065685
ERR1065686
ERR1065687
ERR1065688
ERR1065689
ERR1065690
ERR1065691
ERR1065692
ERR1065694
ERR1065695
ERR1065696
ERR1065698
ERR1065645
ERR1065699
ERR1065701
ERR1065702
ERR1065703
ERR1065704
ERR1065705
ERR1065707
ERR1065708
ERR1065646
ERR1065710
ERR1065711
ERR1065712
ERR1065713
ERR1065714
ERR1065715
ERR1065716
ERR1065717
ERR1065718
ERR1065647
ERR1065719
ERR1065720
ERR1065721
ERR1065722
ERR1065723
ERR1065724
ERR1065725
ERR1065726
ERR1065727
ERR1065728
ERR1065648
ERR1065729
ERR1065730
ERR1065732
ERR1065733
ERR1065734
ERR1065743

ERR1065745
ERR1065746
ERR1065747
ERR1065749
ERR1065750
ERR1065751
ERR1065735
ERR1065754
ERR1065755
ERR1065756
ERR1065757
ERR1065758
ERR1065759
ERR1065760
ERR1065761
ERR1065762
ERR1065763
ERR1065764
ERR1065765
ERR1065766
ERR1065767
ERR1065768
ERR1065769
ERR1065770
ERR1065771
ERR1065772
ERR1065737
ERR1065773
ERR1065775
ERR1065777
ERR1065780
ERR1065781
ERR1065782
ERR1065738
ERR1065783
ERR1065784
ERR1065785
ERR1065786
ERR1065788
ERR1065789
ERR1065791
ERR1065792
ERR1065739
ERR1065794
ERR1065795
ERR1065796
ERR1065797
ERR1065798
ERR1065801
ERR1065802

ERR1065740
ERR1065803
ERR1065804
ERR1065805
ERR1065806
ERR1065807
ERR1065808
ERR1065809
ERR1065810
ERR1065811
ERR1065812
ERR1065741
ERR1065813
ERR1065814
ERR1065815
ERR1065816
ERR1065817
ERR1065818
ERR1065820
ERR1065821
ERR1065822
ERR1065742
ERR1065823
ERR1065824
ERR1065825
ERR1065826
ERR1065827
ERR1065836
ERR1065837
ERR1065838
ERR1065839
ERR1065840
ERR1065841
ERR1065842
ERR1065843
ERR1065844
ERR1065845
ERR1065828
ERR1065846
ERR1065847
ERR1065848
ERR1065849
ERR1065850
ERR1065851
ERR1065852
ERR1065853
ERR1065854
ERR1065855
ERR1065829
ERR1065856

ERR1065857
ERR1065858
ERR1065859
ERR1065860
ERR1065861
ERR1065862
ERR1065863
ERR1065864
ERR1065830
ERR1065866
ERR1065867
ERR1065868
ERR1065869
ERR1065870
ERR1065871
ERR1065872
ERR1065873
ERR1065874
ERR1065875
ERR1065831
ERR1065876
ERR1065877
ERR1065878
ERR1065879
ERR1065880
ERR1065881
ERR1065882
ERR1065883
ERR1065884
ERR1065885
ERR1065832
ERR1065886
ERR1065887
ERR1065888
ERR1065889
ERR1065890
ERR1065891
ERR1065892
ERR1065893
ERR1065894
ERR1065895
ERR1065833
ERR1065896
ERR1065897
ERR1065898
ERR1065899
ERR1065900
ERR1065901
ERR1065902
ERR1065903

ERR1065904
ERR1065905
ERR1065834
ERR1065906
ERR1065908
ERR1065909
ERR1065910
ERR1065911
ERR1065912
ERR1065913
ERR1065914
ERR1065915
ERR1065835
ERR1065916
ERR1065917
ERR1065918
ERR1065929
ERR1065930
ERR1065931
ERR1065933
ERR1065934
ERR1065936
ERR1065938
ERR1065921
ERR1065940
ERR1065942
ERR1065943
ERR1065944
ERR1065945
ERR1065946
ERR1065948
ERR1065922
ERR1065949
ERR1065950
ERR1065951
ERR1065952
ERR1065953
ERR1065954
ERR1065955
ERR1065956
ERR1065957
ERR1065958
ERR1065923
ERR1065959
ERR1065960
ERR1065961
ERR1065962
ERR1065963
ERR1065964
ERR1065965

ERR1065966
ERR1065967
ERR1065924
ERR1065969
ERR1065970
ERR1065971
ERR1065972
ERR1065973
ERR1065974
ERR1065975
ERR1065976
ERR1065977
ERR1065978
ERR1065925
ERR1065979
ERR1065980
ERR1065981
ERR1065986
ERR1065988
ERR1065926
ERR1065989
ERR1065990
ERR1065993
ERR1065994
ERR1065995
ERR1065996
ERR1065997
ERR1065998
ERR1065927
ERR1065999
ERR1066000
ERR1066001
ERR1066002
ERR1066003
ERR1066004
ERR1066005
ERR1066006
ERR1066008
ERR1066009
ERR1066010
ERR1066011
ERR1066012
ERR1066013
ERR1066024
ERR1066026
ERR1066028
ERR1066029
ERR1066030
ERR1066031
ERR1066014

ERR1066032
ERR1066033
ERR1066034
ERR1066035
ERR1066036
ERR1066038
ERR1066039
ERR1066040
ERR1066015
ERR1066042
ERR1066043
ERR1066044
ERR1066045
ERR1066046
ERR1066047
ERR1066048
ERR1066049
ERR1066050
ERR1066051
ERR1066016
ERR1066052
ERR1066053
ERR1066055
ERR1066056
ERR1066057
ERR1066059
ERR1066061
ERR1066017
ERR1066064
ERR1066065
ERR1066066
ERR1066067
ERR1066068
ERR1066069
ERR1066070
ERR1066071
ERR1066018
ERR1066073
ERR1066074
ERR1066076
ERR1066077
ERR1066078
ERR1066079
ERR1066080
ERR1066081
ERR1066019
ERR1066082
ERR1066084
ERR1066085
ERR1066086

ERR1066087
ERR1066088
ERR1066089
ERR1066090
ERR1066091
ERR1066020
ERR1066092
ERR1066093
ERR1066095
ERR1066097
ERR1066098
ERR1066099
ERR1066101
ERR1066021
ERR1066102
ERR1066103
ERR1066112
ERR1066113
ERR1066115
ERR1066116
ERR1066117
ERR1066118
ERR1066119
ERR1066120
ERR1066121
ERR1066104
ERR1066122
ERR1066124
ERR1066125
ERR1066126
ERR1066127
ERR1066128
ERR1066129
ERR1066130
ERR1066131
ERR1066105
ERR1066133
ERR1066134
ERR1066135
ERR1066136
ERR1066137
ERR1066138
ERR1066139
ERR1066141
ERR1066106
ERR1066142
ERR1066143
ERR1066144
ERR1066146
ERR1066147

ERR1066148
ERR1066149
ERR1066151
ERR1066107
ERR1066152
ERR1066153
ERR1066154
ERR1066155
ERR1066156
ERR1066157
ERR1066158
ERR1066160
ERR1066108
ERR1066162
ERR1066163
ERR1066164
ERR1066165
ERR1066166
ERR1066167
ERR1066168
ERR1066169
ERR1066170
ERR1066171
ERR1066109
ERR1066172
ERR1066176
ERR1066177
ERR1066178
ERR1066179
ERR1066180
ERR1066181
ERR1066110
ERR1066182
ERR1066183
ERR1066184
ERR1066185
ERR1066186
ERR1066187
ERR1066188
ERR1066189
ERR1066190
ERR1066191
ERR1066111
ERR1066192
ERR1066193
ERR1066194
ERR1066203
ERR1066204
ERR1066205
ERR1066206

ERR1066207
ERR1066208
ERR1066209
ERR1066210
ERR1066211
ERR1066212
ERR1066195
ERR1066213
ERR1066214
ERR1066215
ERR1066216
ERR1066217
ERR1066218
ERR1066219
ERR1066220
ERR1066221
ERR1066222
ERR1066196
ERR1066223
ERR1066224
ERR1066225
ERR1066226
ERR1066227
ERR1066229
ERR1066230
ERR1066231
ERR1066232
ERR1066197
ERR1066233
ERR1066234
ERR1066236
ERR1066237
ERR1066238
ERR1066239
ERR1066240
ERR1066241
ERR1066242
ERR1066243
ERR1066244
ERR1066245
ERR1066246
ERR1066247
ERR1066248
ERR1066249
ERR1066250
ERR1066251
ERR1066252
ERR1066199
ERR1066253
ERR1066255

ERR1066256
ERR1066257
ERR1066258
ERR1066259
ERR1066260
ERR1066261
ERR1066262
ERR1066200
ERR1066263
ERR1066264
ERR1066265
ERR1066266
ERR1066267
ERR1066268
ERR1066269
ERR1066270
ERR1066272
ERR1066201
ERR1066273
ERR1066274
ERR1066275
ERR1066277
ERR1066278
ERR1066279
ERR1066280
ERR1066281
ERR1066282
ERR1066202
ERR1066283
ERR1066285
ERR1066294
ERR1066295
ERR1066296
ERR1066297
ERR1066298
ERR1066299
ERR1066301
ERR1066302
ERR1066303
ERR1066286
ERR1066304
ERR1066305
ERR1066306
ERR1066307
ERR1066308
ERR1066309
ERR1066310
ERR1066311
ERR1066312
ERR1066313

ERR1066287
ERR1066314
ERR1066316
ERR1066317
ERR1066318
ERR1066319
ERR1066320
ERR1066321
ERR1066322
ERR1066323
ERR1066288
ERR1066324
ERR1066325
ERR1066326
ERR1066327
ERR1066328
ERR1066329
ERR1066330
ERR1066331
ERR1066332
ERR1066333
ERR1066289
ERR1066334
ERR1066335
ERR1066336
ERR1066337
ERR1066338
ERR1066339
ERR1066340
ERR1066341
ERR1066342
ERR1066343
ERR1066290
ERR1066344
ERR1066345
ERR1066346
ERR1066347
ERR1066348
ERR1066349
ERR1066350
ERR1066351
ERR1066352
ERR1066353
ERR1066291
ERR1066354
ERR1066355
ERR1066356
ERR1066357
ERR1066358
ERR1066359

ERR1066360
ERR1066361
ERR1066362
ERR1066363
ERR1066292
ERR1066364
ERR1066365
ERR1066366
ERR1066367
ERR1066368
ERR1066369
ERR1066370
ERR1066371
ERR1066372
ERR1066373
ERR1066293
ERR1066374
ERR1066375
ERR1066376
ERR1066377
ERR1106732
ERR1106733
ERR1106734
ERR1106735
ERR1106736
ERR1106737
ERR1106738
ERR1106739
ERR1106740
ERR1106741
ERR1106742
ERR1106743
ERR1106744
ERR1106745
ERR1106746
ERR1106747
ERR1106748
ERR1106749
ERR1106750
ERR1106751
ERR1106752
ERR1106753
ERR1106754
ERR1106755
ERR1106756
ERR1106757
ERR1106758
ERR1106759
ERR1106760
ERR1106761

ERR1106762
ERR1106763
ERR1106764
ERR1106765
ERR1106766
ERR1106767
ERR1106768
ERR1106769
ERR1106770
ERR1106771
ERR1106772
ERR1106773
ERR1106774
ERR1106775
ERR1106776
ERR1106777
ERR1106778
ERR1106779
ERR1106780
ERR1106781
ERR1106782
ERR1106783
ERR1106784
ERR1106785
ERR1106786
ERR1106787
ERR1106788
ERR1106789
ERR1106790
ERR1106791
ERR1106792
ERR1106793
ERR1106794
ERR1106795
ERR1106796
ERR1106797
ERR1106798
ERR1106801
ERR1106802
ERR1106803
ERR1106804
ERR1106805
ERR1106806
ERR1106807
ERR1106808
ERR1106809
ERR1106810
ERR1106811
ERR1106812
ERR1106813

ERR1191823
ERR1191832
ERR1191922
ERR1191923
ERR1191924
ERR1191925
ERR1191926
ERR1191927
ERR1191928
ERR1191929
ERR1191930
ERR1191931
ERR1191833
ERR1191932
ERR1191933
ERR1191934
ERR1191935
ERR1191936
ERR1191937
ERR1191938
ERR1191940
ERR1191941
ERR1191834
ERR1191942
ERR1191943
ERR1191945
ERR1191946
ERR1191947
ERR1191948
ERR1191949
ERR1191951
ERR1191835
ERR1191952
ERR1191953
ERR1191954
ERR1191955
ERR1191956
ERR1191957
ERR1191958
ERR1191959
ERR1191960
ERR1191836
ERR1191837
ERR1191838
ERR1191839
ERR1191996
ERR1191997
ERR1191998
ERR1191999
ERR1192001

ERR1191840
ERR1192002
ERR1192003
ERR1192004
ERR1192005
ERR1192006
ERR1192007
ERR1192008
ERR1192009
ERR1192010
ERR1191841
ERR1191824
ERR1191842
ERR1191843
ERR1191844
ERR1191845
ERR1191846
ERR1191847
ERR1191848
ERR1191849
ERR1191850
ERR1191851
ERR1191825
ERR1191852
ERR1191853
ERR1191856
ERR1191857
ERR1191859
ERR1191860
ERR1191861
ERR1191826
ERR1191862
ERR1191863
ERR1191864
ERR1191865
ERR1191866
ERR1191867
ERR1191870
ERR1191827
ERR1191872
ERR1191873
ERR1191878
ERR1191879
ERR1191880
ERR1191881
ERR1191828
ERR1191883
ERR1191884
ERR1191885
ERR1191888

ERR1191889
ERR1191890
ERR1191829
ERR1191893
ERR1191895
ERR1191896
ERR1191898
ERR1191899
ERR1191900
ERR1191901
ERR1191830
ERR1191902
ERR1191903
ERR1191906
ERR1191907
ERR1191909
ERR1191910
ERR1191911
ERR1191831
ERR1191912
ERR1191913
ERR1191914
ERR1191915
ERR1191916
ERR1191917
ERR1191918
ERR1191919
ERR1191920
ERR1191921
ERR1192012
ERR1192021
ERR1192111
ERR1192112
ERR1192113
ERR1192114
ERR1192115
ERR1192119
ERR1192120
ERR1192022
ERR1192125
ERR1192128
ERR1192129
ERR1192130
ERR1192023
ERR1192136
ERR1192137
ERR1192138
ERR1192139
ERR1192140
ERR1192024

ERR1192142
ERR1192143
ERR1192145
ERR1192146
ERR1192147
ERR1192149
ERR1192150
ERR1192025
ERR1192151
ERR1192152
ERR1192153
ERR1192154
ERR1192155
ERR1192156
ERR1192157
ERR1192158
ERR1192159
ERR1192160
ERR1192026
ERR1192161
ERR1192162
ERR1192163
ERR1192164
ERR1192165
ERR1192166
ERR1192167
ERR1192168
ERR1192169
ERR1192170
ERR1192027
ERR1192172
ERR1192174
ERR1192178
ERR1192181
ERR1192182
ERR1192185
ERR1192186
ERR1192196
ERR1192197
ERR1192198
ERR1192199
ERR1192200
ERR1192034
ERR1192035
ERR1192036
ERR1192037
ERR1192038
ERR1192040
ERR1192014
ERR1192041

ERR1192042
ERR1192043
ERR1192044
ERR1192045
ERR1192046
ERR1192047
ERR1192048
ERR1192049
ERR1192050
ERR1192015
ERR1192051
ERR1192052
ERR1192053
ERR1192054
ERR1192055
ERR1192056
ERR1192057
ERR1192058
ERR1192059
ERR1192060
ERR1192016
ERR1192061
ERR1192062
ERR1192063
ERR1192064
ERR1192065
ERR1192066
ERR1192067
ERR1192068
ERR1192069
ERR1192070
ERR1192017
ERR1192072
ERR1192073
ERR1192074
ERR1192075
ERR1192076
ERR1192077
ERR1192079
ERR1192080
ERR1192081
ERR1192082
ERR1192083
ERR1192085
ERR1192086
ERR1192087
ERR1192088
ERR1192089
ERR1192090
ERR1192019

ERR1192091
ERR1192092
ERR1192093
ERR1192094
ERR1192095
ERR1192096
ERR1192097
ERR1192098
ERR1192099
ERR1192100
ERR1192020
ERR1192101
ERR1192102
ERR1192103
ERR1192104
ERR1192105
ERR1192106
ERR1192107
ERR1192108
ERR1192109
ERR1192110
ERR1202071
ERR1202080
ERR1202170
ERR1202171
ERR1202172
ERR1202173
ERR1202174
ERR1202175
ERR1202176
ERR1202177
ERR1202178
ERR1202179
ERR1202081
ERR1202180
ERR1202181
ERR1202182
ERR1202183
ERR1202184
ERR1202185
ERR1202187
ERR1202188
ERR1202082
ERR1202192
ERR1202193
ERR1202194
ERR1202195
ERR1202196
ERR1202197
ERR1202198

ERR1202199
ERR1202200
ERR1202201
ERR1202202
ERR1202204
ERR1202205
ERR1202206
ERR1202207
ERR1202208
ERR1202209
ERR1202210
ERR1202211
ERR1202212
ERR1202215
ERR1202216
ERR1202217
ERR1202218
ERR1202219
ERR1202221
ERR1202222
ERR1202223
ERR1202224
ERR1202225
ERR1202226
ERR1202227
ERR1202228
ERR1202229
ERR1202086
ERR1202230
ERR1202231
ERR1202233
ERR1202234
ERR1202235
ERR1202236
ERR1202238
ERR1202239
ERR1202240
ERR1202241
ERR1202242
ERR1202243
ERR1202244
ERR1202245
ERR1202246
ERR1202248
ERR1202249
ERR1202252
ERR1202253
ERR1202254
ERR1202257
ERR1202258

ERR1202259
ERR1202260
ERR1202072
ERR1202093
ERR1202097
ERR1202109
ERR1202110
ERR1202111
ERR1202112
ERR1202113
ERR1202114
ERR1202115
ERR1202116
ERR1202117
ERR1202118
ERR1202119
ERR1202120
ERR1202121
ERR1202122
ERR1202123
ERR1202124
ERR1202125
ERR1202126
ERR1202127
ERR1202128
ERR1202129
ERR1202130
ERR1202131
ERR1202132
ERR1202133
ERR1202134
ERR1202135
ERR1202136
ERR1202137
ERR1202138
ERR1202139
ERR1202140
ERR1202141
ERR1202142
ERR1202143
ERR1202144
ERR1202145
ERR1202146
ERR1202147
ERR1202148
ERR1202149
ERR1202150
ERR1202151
ERR1202152
ERR1202153

ERR1202154
ERR1202155
ERR1202156
ERR1202157
ERR1202158
ERR1202159
ERR1202160
ERR1202161
ERR1202162
ERR1202163
ERR1202164
ERR1202165
ERR1202166
ERR1202167
ERR1202168
ERR1202169
ERR1202261
ERR1202270
ERR1202361
ERR1202363
ERR1202364
ERR1202366
ERR1202367
ERR1202368
ERR1202369
ERR1202271
ERR1202370
ERR1202371
ERR1202373
ERR1202374
ERR1202375
ERR1202376
ERR1202377
ERR1202379
ERR1202380
ERR1202381
ERR1202382
ERR1202383
ERR1202384
ERR1202385
ERR1202386
ERR1202387
ERR1202388
ERR1202389
ERR1202390
ERR1202391
ERR1202392
ERR1202393
ERR1202394
ERR1202395

ERR1202396
ERR1202397
ERR1202398
ERR1202274
ERR1202400
ERR1202401
ERR1202402
ERR1202403
ERR1202404
ERR1202405
ERR1202406
ERR1202407
ERR1202408
ERR1202409
ERR1202411
ERR1202412
ERR1202413
ERR1202414
ERR1202415
ERR1202416
ERR1202417
ERR1202418
ERR1202419
ERR1202276
ERR1202420
ERR1202421
ERR1202422
ERR1202423
ERR1202424
ERR1202425
ERR1202426
ERR1202427
ERR1202428
ERR1202429
ERR1202430
ERR1202431
ERR1202432
ERR1202433
ERR1202434
ERR1202435
ERR1202436
ERR1202438
ERR1202439
ERR1202278
ERR1202440
ERR1202441
ERR1202442
ERR1202443
ERR1202444
ERR1202445

ERR1202447
ERR1202448
ERR1202449
ERR1202279
ERR1202450
ERR1202280
ERR1202282
ERR1202283
ERR1202284
ERR1202285
ERR1202286
ERR1202287
ERR1202288
ERR1202296
ERR1202264
ERR1202300
ERR1202301
ERR1202302
ERR1202303
ERR1202306
ERR1202307
ERR1202308
ERR1202309
ERR1202310
ERR1202311
ERR1202312
ERR1202315
ERR1202319
ERR1202320
ERR1202322
ERR1202326
ERR1202329
ERR1202330
ERR1202331
ERR1202332
ERR1202333
ERR1202336
ERR1202337
ERR1202338
ERR1202339
ERR1202268
ERR1202342
ERR1202348
ERR1202349
ERR1202269
ERR1202350
ERR1202351
ERR1202352
ERR1202353
ERR1202354

ERR1202355
ERR1202357
ERR1202358
ERR1202359
ERR1202451
ERR1202460
ERR1202550
ERR1202551
ERR1202552
ERR1202553
ERR1202554
ERR1202555
ERR1202556
ERR1202557
ERR1202558
ERR1202461
ERR1202560
ERR1202561
ERR1202563
ERR1202564
ERR1202565
ERR1202566
ERR1202567
ERR1202568
ERR1202569
ERR1202462
ERR1202570
ERR1202571
ERR1202572
ERR1202573
ERR1202574
ERR1202575
ERR1202576
ERR1202577
ERR1202578
ERR1202579
ERR1202463
ERR1202580
ERR1202583
ERR1202584
ERR1202585
ERR1202586
ERR1202587
ERR1202588
ERR1202589
ERR1202464
ERR1202590
ERR1202591
ERR1202592
ERR1202593

ERR1202594
ERR1202595
ERR1202596
ERR1202465
ERR1202466
ERR1202467
ERR1202468
ERR1202469
ERR1202452
ERR1202470
ERR1202471
ERR1202472
ERR1202473
ERR1202474
ERR1202475
ERR1202476
ERR1202477
ERR1202453
ERR1202482
ERR1202483
ERR1202484
ERR1202485
ERR1202486
ERR1202487
ERR1202489
ERR1202454
ERR1202490
ERR1202493
ERR1202494
ERR1202496
ERR1202499
ERR1202455
ERR1202500
ERR1202502
ERR1202503
ERR1202504
ERR1202505
ERR1202506
ERR1202507
ERR1202508
ERR1202509
ERR1202456
ERR1202510
ERR1202511
ERR1202512
ERR1202513
ERR1202514
ERR1202516
ERR1202518
ERR1202521

ERR1202522
ERR1202523
ERR1202524
ERR1202525
ERR1202526
ERR1202527
ERR1202528
ERR1202529
ERR1202458
ERR1202530
ERR1202531
ERR1202532
ERR1202533
ERR1202534
ERR1202535
ERR1202536
ERR1202537
ERR1202538
ERR1202539
ERR1202459
ERR1202540
ERR1202541
ERR1202542
ERR1202543
ERR1202544
ERR1202545
ERR1202546
ERR1202547
ERR1202548
ERR1202549
ERR1214420
ERR1214428
ERR1214429
ERR1214430
ERR1214431
ERR1214432
ERR1214433
ERR1214434
ERR1214435
ERR1214436
ERR1214437
ERR1214438
ERR1214439
ERR1214440
ERR1214441
ERR1214442
ERR1214443
ERR1214444
ERR1214445
ERR1214446

ERR1214447
ERR1214448
ERR1214449
ERR1214450
ERR1214451
ERR1214452
ERR1214453
ERR1214454
ERR1214455
ERR1214457
ERR1214464
ERR1214465
ERR1214507
ERR1214508
ERR1214382
ERR1214383
ERR1214385
ERR1214386
ERR1214387
ERR1214388
ERR1214390
ERR1214391
ERR1214392
ERR1214393
ERR1214394
ERR1214396
ERR1214398
ERR1214399
ERR1214401
ERR1214403
ERR1214404
ERR1214405
ERR1214409
ERR1214410
ERR1214411
ERR1214412
ERR1214414
ERR1214415
ERR1214416
ERR1214417
ERR1214418
ERR1214509
ERR1214518
ERR1214608
ERR1214609
ERR1214610
ERR1214611
ERR1214612
ERR1214613
ERR1214614

ERR1214615
ERR1214616
ERR1214617
ERR1214519
ERR1214618
ERR1214619
ERR1214620
ERR1214621
ERR1214622
ERR1214623
ERR1214624
ERR1214625
ERR1214626
ERR1214627
ERR1214520
ERR1214628
ERR1214629
ERR1214630
ERR1214631
ERR1214632
ERR1214633
ERR1214634
ERR1214635
ERR1214636
ERR1214637
ERR1214521
ERR1214638
ERR1214639
ERR1214640
ERR1214641
ERR1214642
ERR1214643
ERR1214645
ERR1214646
ERR1214647
ERR1214522
ERR1214648
ERR1214649
ERR1214650
ERR1214651
ERR1214652
ERR1214653
ERR1214654
ERR1214655
ERR1214656
ERR1214523
ERR1214658
ERR1214659
ERR1214660
ERR1214661

ERR1214662
ERR1214663
ERR1214664
ERR1214665
ERR1214666
ERR1214667
ERR1214524
ERR1214668
ERR1214669
ERR1214670
ERR1214671
ERR1214672
ERR1214673
ERR1214674
ERR1214675
ERR1214676
ERR1214677
ERR1214525
ERR1214678
ERR1214679
ERR1214680
ERR1214681
ERR1214682
ERR1214683
ERR1214684
ERR1214685
ERR1214686
ERR1214526
ERR1214688
ERR1214689
ERR1214690
ERR1214691
ERR1214692
ERR1214693
ERR1214694
ERR1214695
ERR1214696
ERR1214527
ERR1214510
ERR1214528
ERR1214529
ERR1214530
ERR1214531
ERR1214532
ERR1214533
ERR1214534
ERR1214535
ERR1214536
ERR1214537
ERR1214511

ERR1214538
ERR1214539
ERR1214540
ERR1214541
ERR1214542
ERR1214544
ERR1214545
ERR1214546
ERR1214547
ERR1214512
ERR1214548
ERR1214549
ERR1214550
ERR1214551
ERR1214552
ERR1214553
ERR1214554
ERR1214555
ERR1214556
ERR1214557
ERR1214513
ERR1214558
ERR1214559
ERR1214560
ERR1214561
ERR1214562
ERR1214563
ERR1214567
ERR1214514
ERR1214568
ERR1214569
ERR1214570
ERR1214571
ERR1214572
ERR1214573
ERR1214574
ERR1214575
ERR1214576
ERR1214577
ERR1214515
ERR1214578
ERR1214579
ERR1214580
ERR1214581
ERR1214582
ERR1214583
ERR1214584
ERR1214585
ERR1214586
ERR1214587

ERR1214516
ERR1214588
ERR1214589
ERR1214590
ERR1214591
ERR1214592
ERR1214593
ERR1214594
ERR1214595
ERR1214596
ERR1214597
ERR1214517
ERR1214598
ERR1214599
ERR1214600
ERR1214601
ERR1214602
ERR1214603
ERR1214604
ERR1214605
ERR1214606
ERR1214607
ERR1214697
ERR1214698
ERR1214699
ERR1214700
ERR1214703
ERR1214704
ERR1214705
ERR1214706
ERR1214707
ERR1288810
ERR1288819
ERR1288910
ERR1288913
ERR1288914
ERR1288915
ERR1288917
ERR1288918
ERR1288820
ERR1288919
ERR1288920
ERR1288922
ERR1288924
ERR1288925
ERR1288926
ERR1288927
ERR1288928
ERR1288821
ERR1288929

ERR1288930
ERR1288932
ERR1288933
ERR1288934
ERR1288935
ERR1288936
ERR1288937
ERR1288822
ERR1288823
ERR1288824
ERR1288967
ERR1288968
ERR1288825
ERR1288971
ERR1288972
ERR1288973
ERR1288974
ERR1288975
ERR1288976
ERR1288977
ERR1288978
ERR1288826
ERR1288979
ERR1288980
ERR1288981
ERR1288982
ERR1288983
ERR1288984
ERR1288985
ERR1288986
ERR1288987
ERR1288988
ERR1288827
ERR1288989
ERR1288811
ERR1288829
ERR1288830
ERR1288831
ERR1288832
ERR1288833
ERR1288834
ERR1288835
ERR1288836
ERR1288837
ERR1288838
ERR1288812
ERR1288839
ERR1288840
ERR1288841
ERR1288842

ERR1288843
ERR1288844
ERR1288845
ERR1288846
ERR1288847
ERR1288848
ERR1288813
ERR1288849
ERR1288850
ERR1288851
ERR1288852
ERR1288853
ERR1288854
ERR1288855
ERR1288856
ERR1288857
ERR1288858
ERR1288814
ERR1288859
ERR1288860
ERR1288861
ERR1288862
ERR1288863
ERR1288864
ERR1288865
ERR1288866
ERR1288867
ERR1288868
ERR1288815
ERR1288869
ERR1288870
ERR1288871
ERR1288873
ERR1288875
ERR1288876
ERR1288877
ERR1288878
ERR1288816
ERR1288879
ERR1288880
ERR1288881
ERR1288882
ERR1288883
ERR1288884
ERR1288885
ERR1288886
ERR1288887
ERR1288888
ERR1288817
ERR1288889

ERR1288890
ERR1288891
ERR1288892
ERR1288893
ERR1288894
ERR1288895
ERR1288896
ERR1288897
ERR1288818
ERR1288900
ERR1288901
ERR1288902
ERR1288903
ERR1288904
ERR1288905
ERR1288908
ERR1333961
ERR1333962
ERR1333963
ERR1333964
ERR1333965
ERR1333972
ERR1333973
ERR1422339
ERR1422429
ERR1422430
ERR1422431
ERR1422432
ERR1422433
ERR1422434
ERR1422435
ERR1422436
ERR1422437
ERR1422438
ERR1422340
ERR1422439
ERR1422440
ERR1422441
ERR1422442
ERR1422443
ERR1422444
ERR1422445
ERR1422446
ERR1422447
ERR1422448
ERR1422341
ERR1422449
ERR1422450
ERR1422451
ERR1422452

ERR1422453
ERR1422454
ERR1422455
ERR1422456
ERR1422457
ERR1422458
ERR1422342
ERR1422459
ERR1422460
ERR1422461
ERR1422462
ERR1422463
ERR1422464
ERR1422465
ERR1422466
ERR1422467
ERR1422468
ERR1422343
ERR1422469
ERR1422470
ERR1422471
ERR1422472
ERR1422473
ERR1422474
ERR1422475
ERR1422476
ERR1422477
ERR1422478
ERR1422344
ERR1422479
ERR1422480
ERR1422481
ERR1422482
ERR1422483
ERR1422484
ERR1422485
ERR1422486
ERR1422487
ERR1422488
ERR1422345
ERR1422489
ERR1422490
ERR1422491
ERR1422492
ERR1422493
ERR1422494
ERR1422495
ERR1422496
ERR1422497
ERR1422498

ERR1422346
ERR1422499
ERR1422500
ERR1422501
ERR1422502
ERR1422503
ERR1422504
ERR1422505
ERR1422506
ERR1422507
ERR1422508
ERR1422347
ERR1422509
ERR1422510
ERR1422511
ERR1422512
ERR1422513
ERR1422348
ERR1422349
ERR1422350
ERR1422351
ERR1422352
ERR1422353
ERR1422354
ERR1422355
ERR1422356
ERR1422357
ERR1422358
ERR1422359
ERR1422360
ERR1422361
ERR1422362
ERR1422363
ERR1422364
ERR1422365
ERR1422366
ERR1422367
ERR1422368
ERR1422369
ERR1422370
ERR1422371
ERR1422372
ERR1422373
ERR1422374
ERR1422375
ERR1422376
ERR1422377
ERR1422378
ERR1422379
ERR1422380

ERR1422381
ERR1422382
ERR1422383
ERR1422384
ERR1422385
ERR1422386
ERR1422387
ERR1422388
ERR1422389
ERR1422390
ERR1422391
ERR1422392
ERR1422393
ERR1422394
ERR1422395
ERR1422396
ERR1422397
ERR1422336
ERR1422402
ERR1422403
ERR1422404
ERR1422405
ERR1422406
ERR1422407
ERR1422408
ERR1422337
ERR1422409
ERR1422410
ERR1422411
ERR1422412
ERR1422415
ERR1422416
ERR1422417
ERR1422418
ERR1422338
ERR1422419
ERR1422420
ERR1422421
ERR1422422
ERR1422423
ERR1422424
ERR1422425
ERR1422426
ERR1422427
ERR1422428
ERR1422514
ERR1422523
ERR1422613
ERR1422614
ERR1422615

ERR1422616
ERR1422617
ERR1422618
ERR1422619
ERR1422620
ERR1422621
ERR1422622
ERR1422524
ERR1422623
ERR1422624
ERR1422625
ERR1422626
ERR1422627
ERR1422628
ERR1422632
ERR1422525
ERR1422526
ERR1422646
ERR1422647
ERR1422648
ERR1422649
ERR1422650
ERR1422651
ERR1422652
ERR1422527
ERR1422653
ERR1422654
ERR1422658
ERR1422659
ERR1422660
ERR1422661
ERR1422662
ERR1422528
ERR1422663
ERR1422665
ERR1422666
ERR1422667
ERR1422668
ERR1422669
ERR1422670
ERR1422671
ERR1422672
ERR1422529
ERR1422673
ERR1422674
ERR1422675
ERR1422676
ERR1422677
ERR1422678
ERR1422679

ERR1422680
ERR1422681
ERR1422682
ERR1422530
ERR1422683
ERR1422684
ERR1422685
ERR1422686
ERR1422687
ERR1422688
ERR1422689
ERR1422690
ERR1422691
ERR1422692
ERR1422531
ERR1422693
ERR1422694
ERR1422695
ERR1422696
ERR1422532
ERR1422515
ERR1422533
ERR1422534
ERR1422535
ERR1422536
ERR1422537
ERR1422538
ERR1422539
ERR1422540
ERR1422541
ERR1422542
ERR1422516
ERR1422543
ERR1422544
ERR1422545
ERR1422546
ERR1422547
ERR1422548
ERR1422549
ERR1422550
ERR1422551
ERR1422552
ERR1422517
ERR1422553
ERR1422554
ERR1422555
ERR1422556
ERR1422557
ERR1422558
ERR1422559

ERR1422560
ERR1422561
ERR1422562
ERR1422518
ERR1422563
ERR1422564
ERR1422565
ERR1422566
ERR1422567
ERR1422568
ERR1422569
ERR1422570
ERR1422571
ERR1422572
ERR1422519
ERR1422574
ERR1422575
ERR1422576
ERR1422577
ERR1422578
ERR1422579
ERR1422580
ERR1422581
ERR1422582
ERR1422520
ERR1422583
ERR1422584
ERR1422585
ERR1422586
ERR1422588
ERR1422589
ERR1422591
ERR1422592
ERR1422521
ERR1422593
ERR1422594
ERR1422595
ERR1422596
ERR1422597
ERR1422598
ERR1422599
ERR1422600
ERR1422601
ERR1422602
ERR1422522
ERR1422603
ERR1422605
ERR1422606
ERR1422607
ERR1422608

ERR1422609
ERR1422610
ERR1422611
ERR1422612
ERR1430808
ERR1430898
ERR1430899
ERR1430901
ERR1430902
ERR1430903
ERR1430905
ERR1430809
ERR1430810
ERR1430811
ERR1430812
ERR1430813
ERR1430814
ERR1430815
ERR1430817
ERR1430818
ERR1430819
ERR1430820
ERR1430821
ERR1430822
ERR1430823
ERR1430824
ERR1430825
ERR1430826
ERR1430827
ERR1430801
ERR1430828
ERR1430829
ERR1430830
ERR1430831
ERR1430832
ERR1430833
ERR1430834
ERR1430835
ERR1430836
ERR1430837
ERR1430802
ERR1430838
ERR1430839
ERR1430840
ERR1430841
ERR1430842
ERR1430843
ERR1430844
ERR1430845
ERR1430846

ERR1430847
ERR1430803
ERR1430848
ERR1430849
ERR1430850
ERR1430851
ERR1430852
ERR1430853
ERR1430854
ERR1430855
ERR1430856
ERR1430857
ERR1430804
ERR1430858
ERR1430859
ERR1430860
ERR1430861
ERR1430862
ERR1430863
ERR1430864
ERR1430865
ERR1430866
ERR1430867
ERR1430805
ERR1430869
ERR1430870
ERR1430871
ERR1430872
ERR1430873
ERR1430874
ERR1430875
ERR1430876
ERR1430877
ERR1430806
ERR1430878
ERR1430879
ERR1430881
ERR1430882
ERR1430883
ERR1430884
ERR1430885
ERR1430886
ERR1430887
ERR1430807
ERR1430888
ERR1430889
ERR1430890
ERR1430891
ERR1430892
ERR1430893

ERR1430896
ERR1430897
ERR1453567
ERR1453568
ERR1453569
ERR1453570
ERR1453571
ERR1453572
ERR1453573
ERR1453574
ERR1453577
ERR1453578
ERR1453579
ERR1453580
ERR1453581
ERR1453583
ERR1453584
ERR1453585
ERR1453586
ERR1453587
ERR1453588
ERR1453589
ERR1453590
ERR1453591
ERR1453592
ERR1453593
ERR1453594
ERR1453595
ERR1453597
ERR1453598
ERR1453599
ERR1453600
ERR1453601
ERR1453602
ERR1453603
ERR1453604
ERR1453605
ERR1453606
ERR1453607
ERR1453608
ERR1453609
ERR1453611
ERR1453612
ERR1453613
ERR1453614
ERR1453615
ERR1453616
ERR1453617
ERR1453618
ERR1453619

ERR1453620
ERR1453621
ERR1453622
ERR1453623
ERR1453624
ERR1453625
ERR1453626
ERR1453627
ERR1453628
ERR1453629
ERR1453630
ERR1453631
ERR1453632
ERR1453633
ERR1453634
ERR1453635
ERR1453636
ERR1453637
ERR1453638
ERR1453639
ERR1453640
ERR1453641
ERR1453642
ERR1453643
ERR1453644
ERR1453645
ERR1453646
ERR1453647
ERR1453648
ERR1453486
ERR1453490
ERR1453491
ERR1453493
ERR1453494
ERR1453495
ERR1453498
ERR1453499
ERR1453500
ERR1453501
ERR1453502
ERR1453503
ERR1453504
ERR1453505
ERR1453506
ERR1453507
ERR1453508
ERR1453509
ERR1453510
ERR1453511
ERR1453512

ERR1453513
ERR1453514
ERR1453515
ERR1453516
ERR1453517
ERR1453518
ERR1453519
ERR1453520
ERR1453521
ERR1453522
ERR1453523
ERR1453524
ERR1453526
ERR1453527
ERR1453528
ERR1453529
ERR1453530
ERR1453531
ERR1453532
ERR1453533
ERR1453534
ERR1453535
ERR1453536
ERR1453537
ERR1453538
ERR1453540
ERR1453541
ERR1453542
ERR1453543
ERR1453544
ERR1453545
ERR1453546
ERR1453547
ERR1453548
ERR1453549
ERR1453550
ERR1453551
ERR1453553
ERR1453554
ERR1453555
ERR1453556
ERR1453557
ERR1453558
ERR1453559
ERR1453560
ERR1453561
ERR1453562
ERR1453563
ERR1453564
ERR1453565

ERR1453566
ERR1453649
ERR1453658
ERR1453748
ERR1453749
ERR1453750
ERR1453751
ERR1453752
ERR1453753
ERR1453754
ERR1453755
ERR1453756
ERR1453757
ERR1453659
ERR1453758
ERR1453759
ERR1453760
ERR1453761
ERR1453762
ERR1453763
ERR1453764
ERR1453765
ERR1453766
ERR1453767
ERR1453660
ERR1453768
ERR1453769
ERR1453770
ERR1453771
ERR1453772
ERR1453773
ERR1453774
ERR1453775
ERR1453776
ERR1453777
ERR1453661
ERR1453778
ERR1453779
ERR1453662
ERR1453664
ERR1453665
ERR1453666
ERR1453667
ERR1453650
ERR1453668
ERR1453669
ERR1453670
ERR1453671
ERR1453673
ERR1453674

ERR1453675
ERR1453676
ERR1453677
ERR1453651
ERR1453678
ERR1453679
ERR1453680
ERR1453681
ERR1453682
ERR1453683
ERR1453684
ERR1453685
ERR1453686
ERR1453687
ERR1453652
ERR1453688
ERR1453689
ERR1453690
ERR1453691
ERR1453692
ERR1453693
ERR1453694
ERR1453695
ERR1453696
ERR1453697
ERR1453653
ERR1453698
ERR1453699
ERR1453700
ERR1453701
ERR1453702
ERR1453703
ERR1453704
ERR1453705
ERR1453706
ERR1453707
ERR1453654
ERR1453708
ERR1453709
ERR1453710
ERR1453711
ERR1453713
ERR1453714
ERR1453715
ERR1453716
ERR1453717
ERR1453655
ERR1453718
ERR1453719
ERR1453720

ERR1453721
ERR1453722
ERR1453723
ERR1453724
ERR1453725
ERR1453726
ERR1453727
ERR1453656
ERR1453728
ERR1453729
ERR1453730
ERR1453731
ERR1453732
ERR1453733
ERR1453734
ERR1453735
ERR1453736
ERR1453737
ERR1453657
ERR1453738
ERR1453739
ERR1453740
ERR1453741
ERR1453742
ERR1453743
ERR1453744
ERR1453745
ERR1453746
ERR1453747
ERR1540700
ERR1540701
ERR1540702
ERR1540703
ERR1540704
ERR1540705
ERR1540706
ERR1583705
ERR1583711
ERR1583714
ERR1638454
ERR1638455
ERR1638457
ERR1638463
ERR1638464
ERR1638466
ERR1756395
ERR1756485
ERR1756486
ERR1756487
ERR1756488

ERR1756489
ERR1756490
ERR1756491
ERR1756492
ERR1756398
ERR1756399
ERR1756400
ERR1756401
ERR1756403
ERR1756404
ERR1756387
ERR1756405
ERR1756406
ERR1756407
ERR1756408
ERR1756409
ERR1756410
ERR1756411
ERR1756413
ERR1756414
ERR1756415
ERR1756416
ERR1756417
ERR1756419
ERR1756420
ERR1756422
ERR1756424
ERR1756389
ERR1756426
ERR1756427
ERR1756430
ERR1756432
ERR1756433
ERR1756434
ERR1756390
ERR1756435
ERR1756436
ERR1756437
ERR1756438
ERR1756440
ERR1756441
ERR1756442
ERR1756443
ERR1756444
ERR1756391
ERR1756445
ERR1756446
ERR1756447
ERR1756448
ERR1756449

ERR1756450
ERR1756451
ERR1756452
ERR1756453
ERR1756454
ERR1756392
ERR1756455
ERR1756456
ERR1756457
ERR1756458
ERR1756460
ERR1756461
ERR1756462
ERR1756464
ERR1756465
ERR1756466
ERR1756468
ERR1756469
ERR1756472
ERR1756473
ERR1756474
ERR1756476
ERR1756477
ERR1756478
ERR1756481
ERR1756482
ERR1756483
ERR1756484
ERR1764035
ERR1764036
ERR1764037
ERR1764038
ERR1764039
ERR1764040
ERR1764041
ERR1764042
ERR1764043
ERR1764044
ERR1764045
ERR1764046
ERR1764047
ERR1764048
ERR1764049
ERR1764050
ERR1764051
ERR1764052
ERR1764053
ERR1764054
ERR1764055
ERR1764056

ERR1764057
ERR1764058
ERR1764059
ERR1788139
ERR1788140
ERR1788141
ERR1788142
ERR1788143
ERR1788144
ERR1788145
ERR1788146
ERR1788147
ERR1788148
ERR1788149
ERR1788150
ERR1788151
ERR1788152
ERR1788153
ERR1788154
ERR1788155
ERR1788156
ERR1788157
ERR1788158
ERR1788159
ERR1788160
ERR1788161
ERR1788162
ERR1788163
ERR1788164
ERR1788165
ERR1788167
ERR1788169
ERR1788170
ERR1788171
ERR1788172
ERR1788173
ERR1788174
ERR1788176
ERR1788177
ERR1788178
ERR1788179
ERR1788180
ERR1788181
ERR1788182
ERR1788183
ERR1788184
ERR1788186
ERR1788187
ERR1788189
ERR1788191

ERR1788192
ERR1788193
ERR1788194
ERR1788195
ERR1788196
ERR1788197
ERR1788198
ERR1788199
ERR1788200
ERR1788209
ERR1788298
ERR1788300
ERR1788302
ERR1788303
ERR1788304
ERR1788305
ERR1788306
ERR1788307
ERR1788210
ERR1788308
ERR1788315
ERR1788317
ERR1788211
ERR1788318
ERR1788320
ERR1788321
ERR1788322
ERR1788323
ERR1788324
ERR1788212
ERR1788213
ERR1788214
ERR1788354
ERR1788355
ERR1788356
ERR1788215
ERR1788359
ERR1788360
ERR1788361
ERR1788362
ERR1788363
ERR1788216
ERR1788367
ERR1788368
ERR1788369
ERR1788370
ERR1788371
ERR1788372
ERR1788375
ERR1788376

ERR1788217
ERR1788378
ERR1788379
ERR1788380
ERR1788381
ERR1788382
ERR1788383
ERR1788386
ERR1788218
ERR1788201
ERR1788219
ERR1788220
ERR1788221
ERR1788222
ERR1788223
ERR1788224
ERR1788225
ERR1788226
ERR1788228
ERR1788202
ERR1788229
ERR1788230
ERR1788232
ERR1788233
ERR1788234
ERR1788236
ERR1788237
ERR1788203
ERR1788238
ERR1788239
ERR1788240
ERR1788241
ERR1788242
ERR1788243
ERR1788244
ERR1788246
ERR1788247
ERR1788204
ERR1788248
ERR1788250
ERR1788251
ERR1788252
ERR1788253
ERR1788254
ERR1788255
ERR1788256
ERR1788257
ERR1788205
ERR1788258
ERR1788261

ERR1788262
ERR1788265
ERR1788206
ERR1788268
ERR1788269
ERR1788270
ERR1788271
ERR1788273
ERR1788274
ERR1788275
ERR1788276
ERR1788277
ERR1788207
ERR1788278
ERR1788279
ERR1788281
ERR1788282
ERR1788283
ERR1788285
ERR1788287
ERR1788208
ERR1788288
ERR1788290
ERR1788291
ERR1788292
ERR1788293
ERR1788294
ERR1788295
ERR1788297
ERR1788387
ERR1788396
ERR1788485
ERR1788486
ERR1788487
ERR1788488
ERR1788489
ERR1788490
ERR1788491
ERR1788492
ERR1788493
ERR1788494
ERR1788397
ERR1788495
ERR1788496
ERR1788497
ERR1788498
ERR1788499
ERR1788500
ERR1788501
ERR1788502

ERR1788503
ERR1788504
ERR1788398
ERR1788505
ERR1788506
ERR1788507
ERR1788508
ERR1788511
ERR1788512
ERR1788513
ERR1788514
ERR1788399
ERR1788515
ERR1788516
ERR1788517
ERR1788519
ERR1788521
ERR1788522
ERR1788523
ERR1788524
ERR1788400
ERR1788525
ERR1788526
ERR1788527
ERR1788528
ERR1788529
ERR1788530
ERR1788531
ERR1788533
ERR1788534
ERR1788535
ERR1788537
ERR1788538
ERR1788539
ERR1788540
ERR1788541
ERR1788542
ERR1788543
ERR1788402
ERR1788545
ERR1788546
ERR1788547
ERR1788549
ERR1788550
ERR1788551
ERR1788552
ERR1788553
ERR1788554
ERR1788403
ERR1788555

ERR1788556
ERR1788558
ERR1788559
ERR1788560
ERR1788561
ERR1788562
ERR1788563
ERR1788564
ERR1788404
ERR1788566
ERR1788567
ERR1788568
ERR1788569
ERR1788570
ERR1788571
ERR1788572
ERR1788573
ERR1788574
ERR1788388
ERR1788407
ERR1788408
ERR1788409
ERR1788410
ERR1788411
ERR1788412
ERR1788415
ERR1788417
ERR1788418
ERR1788421
ERR1788423
ERR1788390
ERR1788426
ERR1788427
ERR1788428
ERR1788429
ERR1788430
ERR1788431
ERR1788432
ERR1788433
ERR1788434
ERR1788391
ERR1788435
ERR1788436
ERR1788437
ERR1788438
ERR1788439
ERR1788440
ERR1788441
ERR1788442
ERR1788443

ERR1788444
ERR1788392
ERR1788445
ERR1788446
ERR1788447
ERR1788448
ERR1788449
ERR1788451
ERR1788452
ERR1788453
ERR1788454
ERR1788393
ERR1788455
ERR1788456
ERR1788457
ERR1788458
ERR1788459
ERR1788460
ERR1788461
ERR1788462
ERR1788463
ERR1788464
ERR1788394
ERR1788465
ERR1788466
ERR1788467
ERR1788468
ERR1788469
ERR1788470
ERR1788471
ERR1788472
ERR1788473
ERR1788474
ERR1788395
ERR1788475
ERR1788476
ERR1788477
ERR1788478
ERR1788479
ERR1788480
ERR1788481
ERR1788482
ERR1788483
ERR1788484
ERR1795451
ERR1795452
ERR1795453
ERR1795455
ERR1795456
ERR1795457

ERR1795458
ERR1795459
ERR1795460
ERR1795461
ERR1795464
ERR1795465
ERR1795466
ERR1795467
ERR1795468
ERR1795469
ERR1795470
ERR1795471
ERR1795472
ERR1795473
ERR1795474
ERR1795475
ERR1795476
ERR1795477
ERR1795478
ERR1795480
ERR1795481
ERR1795482
ERR1795484
ERR1795485
ERR1795487
ERR1836939
ERR1836940
ERR1836941
ERR1836942
ERR2089362
ERR2089363
ERR2089364
ERR2089365
ERR2089366
ERR2089367
ERR2089368
ERR2089369
ERR2089370
ERR2089371
ERR2089372
ERR2089373
ERR2089374
ERR2089375
ERR2089376
ERR2089377
ERR2089378
ERR2089379
ERR2089380
ERR2089381
ERR2089382

ERR2089383
ERR2089384
ERR2089385
ERR2089386
ERR2089387
ERR2089388
ERR2089389
ERR2089390
ERR2089391
ERR2089392
ERR2089393
ERR2089394
ERR2089395
ERR2089396
ERR2089397
ERR2089398
ERR2089399
ERR2089400
ERR2089401
ERR2089402
ERR2089403
ERR2089404
ERR2089405
ERR2089406
ERR2089407
ERR2089408
ERR2089409
ERR2089410
ERR2089411
ERR2089412
ERR2089413
ERR2089414
ERR2089415
ERR2089416
ERR2089417
ERR2089418
ERR2089419
ERR2089421
ERR2089422
ERR2089423
ERR2089424
ERR2089425
ERR2089427
ERR2089428
ERR2089429
ERR2089430
ERR2089431
ERR2089432
ERR2089433
ERR2089434

ERR2089435
ERR2089436
ERR2089437
ERR2089438
ERR2089439
ERR2089440
ERR2089442
ERR2089443
ERR2089444
ERR2089445
ERR2089358
ERR2089359
ERR2089360
ERR2089361
ERR2089446
ERR2089455
ERR2089545
ERR2089546
ERR2089547
ERR2089548
ERR2089550
ERR2089551
ERR2089552
ERR2089553
ERR2089554
ERR2089456
ERR2089555
ERR2089556
ERR2089557
ERR2089558
ERR2089559
ERR2089560
ERR2089561
ERR2089562
ERR2089564
ERR2089457
ERR2089565
ERR2089566
ERR2089567
ERR2089569
ERR2089570
ERR2089572
ERR2089458
ERR2089459
ERR2089460
ERR2089461
ERR2089462
ERR2089463
ERR2089447
ERR2089465

ERR2089466
ERR2089467
ERR2089468
ERR2089469
ERR2089470
ERR2089471
ERR2089472
ERR2089473
ERR2089474
ERR2089448
ERR2089475
ERR2089476
ERR2089477
ERR2089480
ERR2089481
ERR2089482
ERR2089483
ERR2089484
ERR2089449
ERR2089485
ERR2089486
ERR2089487
ERR2089488
ERR2089489
ERR2089490
ERR2089491
ERR2089492
ERR2089493
ERR2089494
ERR2089450
ERR2089495
ERR2089496
ERR2089497
ERR2089498
ERR2089499
ERR2089500
ERR2089503
ERR2089504
ERR2089451
ERR2089506
ERR2089507
ERR2089508
ERR2089509
ERR2089510
ERR2089511
ERR2089512
ERR2089513
ERR2089514
ERR2089452
ERR2089516

ERR2089517
ERR2089518
ERR2089519
ERR2089522
ERR2089524
ERR2089453
ERR2089525
ERR2089526
ERR2089527
ERR2089528
ERR2089529
ERR2089530
ERR2089531
ERR2089532
ERR2089533
ERR2089534
ERR2089454
ERR2089535
ERR2089536
ERR2089537
ERR2089538
ERR2089539
ERR2089540
ERR2089541
ERR2089542
ERR2089543
ERR2089544
ERR2089721
ERR2089722
ERR2089723
ERR2089724
ERR2089725
ERR2089726
ERR2089727
ERR2089728
ERR2089729
ERR2089730
ERR2089731
ERR2089732
ERR2089733
ERR2089734
ERR2089735
ERR2089736
ERR2089737
ERR2089738
ERR2089739
ERR2089740
ERR2089741
ERR2089742
ERR2089743

ERR2089744
ERR2089745
ERR2089746
ERR2089747
ERR2089748
ERR2089749
ERR2089750
ERR2089751
ERR2089752
ERR2089753
ERR2089754
ERR2089755
ERR2089756
ERR2089757
ERR2089758
ERR2089759
ERR2089760
ERR2089761
ERR2089762
ERR2089763
ERR2089764
ERR2089765
ERR2089766
ERR2089767
ERR2089768
ERR2089769
ERR2089770
ERR2089771
ERR2089772
ERR2089773
ERR2089774
ERR2089775
ERR2089776
ERR2089777
ERR2089778
ERR2089779
ERR2089780
ERR2089781
ERR2089782
ERR2089783
ERR2089784
ERR2089786
ERR2089639
ERR2089640
ERR2089641
ERR2089642
ERR2089643
ERR2089644
ERR2089645
ERR2089646

ERR2089647
ERR2089648
ERR2089649
ERR2089650
ERR2089651
ERR2089652
ERR2089653
ERR2089654
ERR2089655
ERR2089656
ERR2089657
ERR2089658
ERR2089659
ERR2089661
ERR2089662
ERR2089663
ERR2089664
ERR2089665
ERR2089666
ERR2089667
ERR2089668
ERR2089669
ERR2089670
ERR2089671
ERR2089672
ERR2089673
ERR2089674
ERR2089675
ERR2089676
ERR2089677
ERR2089679
ERR2089680
ERR2089681
ERR2089682
ERR2089683
ERR2089684
ERR2089685
ERR2089686
ERR2089687
ERR2089688
ERR2089689
ERR2089690
ERR2089691
ERR2089692
ERR2089693
ERR2089695
ERR2089696
ERR2089697
ERR2089698
ERR2089699

ERR2089700
ERR2089701
ERR2089702
ERR2089703
ERR2089704
ERR2089705
ERR2089706
ERR2089707
ERR2089708
ERR2089709
ERR2089710
ERR2089711
ERR2089712
ERR2089713
ERR2089714
ERR2089715
ERR2089716
ERR2089717
ERR2089718
ERR2089789
ERR2089798
ERR2089799
ERR2089800
ERR2089801
ERR2089802
ERR2089803
ERR2089804
ERR2089805
ERR2089806
ERR2089807
ERR2089790
ERR2089808
ERR2089809
ERR2089810
ERR2089811
ERR2089812
ERR2089813
ERR2089814
ERR2089815
ERR2089816
ERR2089817
ERR2089791
ERR2089818
ERR2089819
ERR2089820
ERR2089821
ERR2089822
ERR2089823
ERR2089824
ERR2089825

ERR2089826
ERR2089827
ERR2089792
ERR2089828
ERR2089829
ERR2089830
ERR2089831
ERR2089832
ERR2089833
ERR2089834
ERR2089835
ERR2089836
ERR2089837
ERR2089793
ERR2089838
ERR2089839
ERR2089840
ERR2089841
ERR2089842
ERR2089843
ERR2089844
ERR2089845
ERR2089846
ERR2089847
ERR2089794
ERR2089848
ERR2089849
ERR2089850
ERR2089851
ERR2089852
ERR2089853
ERR2089854
ERR2089855
ERR2089856
ERR2089857
ERR2089795
ERR2089858
ERR2089859
ERR2089860
ERR2089862
ERR2089863
ERR2089864
ERR2089865
ERR2089866
ERR2089867
ERR2089796
ERR2089868
ERR2089869
ERR2089870
ERR2089871

ERR2089872
ERR2089873
ERR2089874
ERR2089875
ERR2089876
ERR2089797
ERR2090393
ERR2090394
ERR2090395
ERR2090396
ERR2090397
ERR2090398
ERR2090399
ERR2090400
ERR2090401
ERR2090402
ERR2090403
ERR2090404
ERR2090405
ERR2090406
ERR2090407
ERR2090408
ERR2090409
ERR2090410
ERR2090411
ERR2090412
ERR2090413
ERR2090414
ERR2090415
ERR2090416
ERR2090417
ERR2090418
ERR2090419
ERR2090420
ERR2090421
ERR2090422
ERR2090423
ERR2090424
ERR2090425
ERR2090426
ERR2090427
ERR2090428
ERR2090429
ERR2090430
ERR2090431
ERR2090432
ERR2090433
ERR2090434
ERR2090435
ERR2090436

ERR2090437
ERR2090438
ERR2090439
ERR2090440
ERR2090441
ERR2090442
ERR2090443
ERR2090444
ERR2090445
ERR2090446
ERR2090447
ERR2090448
ERR2090449
ERR2090450
ERR2090451
ERR2090452
ERR2090453
ERR2090454
ERR2090455
ERR2090456
ERR2090457
ERR2090458
ERR2090459
ERR2090460
ERR2090461
ERR2090462
ERR2090463
ERR2090505
ERR2090506
ERR2090507
ERR2090508
ERR2090509
ERR2090510
ERR2090511
ERR2090512
ERR2090513
ERR2090514
ERR2090515
ERR2090516
ERR2090517
ERR2090519
ERR2090520
ERR2090521
ERR2090522
ERR2090523
ERR2090524
ERR2090525
ERR2090526
ERR2090529
ERR2090530

ERR2090531
ERR2090532
ERR2090533
ERR2090534
ERR2090535
ERR2090536
ERR2090537
ERR2090539
ERR2090540
ERR2090541
ERR2090542
ERR2090551
ERR2090641
ERR2090642
ERR2090643
ERR2090644
ERR2090645
ERR2090646
ERR2090647
ERR2090648
ERR2090649
ERR2090650
ERR2090552
ERR2090651
ERR2090652
ERR2090653
ERR2090654
ERR2090655
ERR2090656
ERR2090657
ERR2090658
ERR2090659
ERR2090660
ERR2090553
ERR2090661
ERR2090662
ERR2090663
ERR2090664
ERR2090665
ERR2090666
ERR2090667
ERR2090668
ERR2090669
ERR2090670
ERR2090671
ERR2090673
ERR2090674
ERR2090675
ERR2090676
ERR2090677

ERR2090678
ERR2090679
ERR2090680
ERR2090555
ERR2090681
ERR2090682
ERR2090683
ERR2090684
ERR2090685
ERR2090686
ERR2090687
ERR2090688
ERR2090689
ERR2090690
ERR2090556
ERR2090691
ERR2090692
ERR2090693
ERR2090694
ERR2090695
ERR2090696
ERR2090697
ERR2090698
ERR2090699
ERR2090700
ERR2090557
ERR2090701
ERR2090702
ERR2090703
ERR2090704
ERR2090705
ERR2090706
ERR2090707
ERR2090708
ERR2090709
ERR2090710
ERR2090558
ERR2090711
ERR2090712
ERR2090713
ERR2090714
ERR2090715
ERR2090716
ERR2090717
ERR2090718
ERR2090719
ERR2090720
ERR2090559
ERR2090721
ERR2090722

ERR2090723
ERR2090724
ERR2090725
ERR2090726
ERR2090727
ERR2090728
ERR2090729
ERR2090730
ERR2090560
ERR2090731
ERR2090543
ERR2090561
ERR2090562
ERR2090563
ERR2090564
ERR2090565
ERR2090566
ERR2090567
ERR2090568
ERR2090569
ERR2090570
ERR2090544
ERR2090571
ERR2090572
ERR2090573
ERR2090574
ERR2090576
ERR2090577
ERR2090578
ERR2090579
ERR2090580
ERR2090545
ERR2090581
ERR2090582
ERR2090583
ERR2090584
ERR2090585
ERR2090586
ERR2090587
ERR2090588
ERR2090589
ERR2090590
ERR2090546
ERR2090591
ERR2090592
ERR2090593
ERR2090594
ERR2090595
ERR2090596
ERR2090597

ERR2090598
ERR2090599
ERR2090600
ERR2090547
ERR2090601
ERR2090602
ERR2090603
ERR2090604
ERR2090605
ERR2090606
ERR2090607
ERR2090608
ERR2090609
ERR2090610
ERR2090548
ERR2090611
ERR2090613
ERR2090615
ERR2090616
ERR2090617
ERR2090619
ERR2090549
ERR2090621
ERR2090622
ERR2090623
ERR2090624
ERR2090625
ERR2090626
ERR2090627
ERR2090628
ERR2090629
ERR2090630
ERR2090550
ERR2090631
ERR2090632
ERR2090633
ERR2090634
ERR2090635
ERR2090636
ERR2090637
ERR2090638
ERR2090639
ERR2090640
ERR2090732
ERR2090741
ERR2090831
ERR2090832
ERR2090833
ERR2090834
ERR2090835

ERR2090836
ERR2090837
ERR2090838
ERR2090839
ERR2090840
ERR2090742
ERR2090841
ERR2090842
ERR2090843
ERR2090844
ERR2090845
ERR2090847
ERR2090848
ERR2090849
ERR2090850
ERR2090743
ERR2090851
ERR2090853
ERR2090854
ERR2090856
ERR2090857
ERR2090858
ERR2090859
ERR2090860
ERR2090744
ERR2090861
ERR2090862
ERR2090863
ERR2090864
ERR2090865
ERR2090866
ERR2090867
ERR2090868
ERR2090869
ERR2090870
ERR2090745
ERR2090871
ERR2090872
ERR2090873
ERR2090874
ERR2090875
ERR2090876
ERR2090877
ERR2090878
ERR2090879
ERR2090880
ERR2090746
ERR2090881
ERR2090882
ERR2090883

ERR2090884
ERR2090885
ERR2090886
ERR2090887
ERR2090888
ERR2090889
ERR2090890
ERR2090747
ERR2090891
ERR2090892
ERR2090893
ERR2090894
ERR2090895
ERR2090896
ERR2090897
ERR2090898
ERR2090899
ERR2090900
ERR2090748
ERR2090901
ERR2090902
ERR2090903
ERR2090904
ERR2090905
ERR2090906
ERR2090907
ERR2090908
ERR2090909
ERR2090910
ERR2090749
ERR2090911
ERR2090912
ERR2090913
ERR2090914
ERR2090915
ERR2090917
ERR2090918
ERR2090919
ERR2090920
ERR2090750
ERR2090921
ERR2090733
ERR2090751
ERR2090752
ERR2090753
ERR2090754
ERR2090755
ERR2090756
ERR2090757
ERR2090758

ERR2090759
ERR2090760
ERR2090734
ERR2090761
ERR2090762
ERR2090763
ERR2090764
ERR2090765
ERR2090766
ERR2090767
ERR2090768
ERR2090769
ERR2090770
ERR2090735
ERR2090771
ERR2090772
ERR2090773
ERR2090774
ERR2090775
ERR2090776
ERR2090777
ERR2090778
ERR2090779
ERR2090780
ERR2090736
ERR2090781
ERR2090782
ERR2090783
ERR2090784
ERR2090785
ERR2090786
ERR2090787
ERR2090788
ERR2090789
ERR2090790
ERR2090737
ERR2090791
ERR2090792
ERR2090793
ERR2090794
ERR2090795
ERR2090796
ERR2090797
ERR2090798
ERR2090799
ERR2090800
ERR2090738
ERR2090801
ERR2090802
ERR2090803

ERR2090804
ERR2090805
ERR2090806
ERR2090807
ERR2090808
ERR2090809
ERR2090810
ERR2090739
ERR2090811
ERR2090812
ERR2090813
ERR2090814
ERR2090815
ERR2090816
ERR2090817
ERR2090818
ERR2090819
ERR2090820
ERR2090740
ERR2090821
ERR2090822
ERR2090823
ERR2090824
ERR2090825
ERR2090826
ERR2090827
ERR2090828
ERR2090829
ERR2090830
ERR2090922
ERR2091021
ERR2091022
ERR2091023
ERR2091024
ERR2091025
ERR2091026
ERR2091027
ERR2091028
ERR2091029
ERR2091030
ERR2090932
ERR2091031
ERR2091032
ERR2091033
ERR2091034
ERR2091035
ERR2091036
ERR2091037
ERR2091038
ERR2091039

ERR2091040
ERR2090933
ERR2091041
ERR2091042
ERR2091043
ERR2091044
ERR2091045
ERR2091046
ERR2091047
ERR2091048
ERR2091049
ERR2091050
ERR2090934
ERR2091051
ERR2091052
ERR2091053
ERR2091054
ERR2091055
ERR2091056
ERR2091057
ERR2091058
ERR2091059
ERR2091060
ERR2090935
ERR2091061
ERR2091062
ERR2091065
ERR2091066
ERR2091067
ERR2091068
ERR2091069
ERR2091070
ERR2090936
ERR2091071
ERR2091072
ERR2091073
ERR2091074
ERR2091075
ERR2091076
ERR2091077
ERR2091078
ERR2091079
ERR2091080
ERR2090937
ERR2091081
ERR2091082
ERR2091083
ERR2091084
ERR2091085
ERR2091086

ERR2091087
ERR2091088
ERR2091089
ERR2091090
ERR2090938
ERR2091091
ERR2091092
ERR2091093
ERR2091094
ERR2091095
ERR2091096
ERR2091097
ERR2091098
ERR2091099
ERR2091100
ERR2090939
ERR2091101
ERR2091102
ERR2091103
ERR2091104
ERR2091105
ERR2091106
ERR2091107
ERR2091108
ERR2091109
ERR2090923
ERR2090941
ERR2090942
ERR2090943
ERR2090944
ERR2090945
ERR2090946
ERR2090947
ERR2090948
ERR2090949
ERR2090950
ERR2090924
ERR2090951
ERR2090952
ERR2090953
ERR2090954
ERR2090955
ERR2090956
ERR2090957
ERR2090958
ERR2090959
ERR2090960
ERR2090925
ERR2090961
ERR2090962

ERR2090963
ERR2090964
ERR2090965
ERR2090966
ERR2090967
ERR2090968
ERR2090969
ERR2090970
ERR2090926
ERR2090971
ERR2090972
ERR2090973
ERR2090974
ERR2090975
ERR2090976
ERR2090977
ERR2090978
ERR2090979
ERR2090980
ERR2090927
ERR2090981
ERR2090982
ERR2090983
ERR2090984
ERR2090985
ERR2090986
ERR2090987
ERR2090988
ERR2090989
ERR2090990
ERR2090928
ERR2090991
ERR2090992
ERR2090993
ERR2090994
ERR2090995
ERR2090996
ERR2090997
ERR2090998
ERR2090999
ERR2091000
ERR2090929
ERR2091001
ERR2091002
ERR2091003
ERR2091004
ERR2091005
ERR2091006
ERR2091008
ERR2091009

ERR2090930
ERR2091011
ERR2091012
ERR2091013
ERR2091014
ERR2091015
ERR2091016
ERR2091017
ERR2091018
ERR2091019
ERR2091020
ERR2091110
ERR2091119
ERR2091209
ERR2091210
ERR2091211
ERR2091212
ERR2091214
ERR2091215
ERR2091216
ERR2091217
ERR2091218
ERR2091120
ERR2091219
ERR2091220
ERR2091221
ERR2091222
ERR2091223
ERR2091224
ERR2091225
ERR2091226
ERR2091227
ERR2091228
ERR2091121
ERR2091229
ERR2091230
ERR2091231
ERR2091232
ERR2091233
ERR2091234
ERR2091235
ERR2091236
ERR2091237
ERR2091238
ERR2091122
ERR2091239
ERR2091240
ERR2091241
ERR2091242
ERR2091243

ERR2091244
ERR2091245
ERR2091246
ERR2091247
ERR2091248
ERR2091123
ERR2091249
ERR2091250
ERR2091251
ERR2091252
ERR2091253
ERR2091254
ERR2091255
ERR2091256
ERR2091257
ERR2091258
ERR2091124
ERR2091259
ERR2091260
ERR2091261
ERR2091262
ERR2091263
ERR2091264
ERR2091265
ERR2091266
ERR2091267
ERR2091268
ERR2091125
ERR2091269
ERR2091270
ERR2091271
ERR2091272
ERR2091273
ERR2091274
ERR2091275
ERR2091276
ERR2091277
ERR2091278
ERR2091126
ERR2091279
ERR2091280
ERR2091281
ERR2091282
ERR2091283
ERR2091284
ERR2091285
ERR2091286
ERR2091287
ERR2091288
ERR2091127

ERR2091289
ERR2091290
ERR2091291
ERR2091292
ERR2091293
ERR2091294
ERR2091295
ERR2091296
ERR2091297
ERR2091128
ERR2091111
ERR2091129
ERR2091130
ERR2091131
ERR2091132
ERR2091133
ERR2091134
ERR2091135
ERR2091136
ERR2091137
ERR2091138
ERR2091112
ERR2091139
ERR2091140
ERR2091141
ERR2091142
ERR2091143
ERR2091144
ERR2091145
ERR2091146
ERR2091147
ERR2091148
ERR2091113
ERR2091149
ERR2091150
ERR2091151
ERR2091152
ERR2091153
ERR2091154
ERR2091155
ERR2091156
ERR2091157
ERR2091158
ERR2091114
ERR2091159
ERR2091160
ERR2091161
ERR2091162
ERR2091163
ERR2091164

ERR2091165
ERR2091166
ERR2091167
ERR2091168
ERR2091115
ERR2091169
ERR2091170
ERR2091171
ERR2091172
ERR2091173
ERR2091174
ERR2091175
ERR2091176
ERR2091177
ERR2091178
ERR2091116
ERR2091179
ERR2091180
ERR2091181
ERR2091182
ERR2091183
ERR2091184
ERR2091185
ERR2091186
ERR2091187
ERR2091188
ERR2091117
ERR2091189
ERR2091190
ERR2091191
ERR2091192
ERR2091193
ERR2091194
ERR2091195
ERR2091196
ERR2091197
ERR2091118
ERR2091199
ERR2091201
ERR2091202
ERR2091203
ERR2091204
ERR2091205
ERR2091207
ERR2091208
ERR2612060
ERR2612061
ERR2612063
ERR2612064
ERR2612065

ERR2612066
ERR2612067
ERR2612069
ERR2612070
ERR2612071
ERR2612072
ERR2612073
ERR2612074
ERR2612075
ERR2612076
ERR2612077
ERR2612078
ERR2612079
ERR2612080
ERR2612081
ERR2612082
ERR2612083
ERR2612084
ERR2612085
ERR2612086
ERR2612087
ERR2612088
ERR2612089
ERR2612090
ERR2612091
ERR2612092
ERR2612093
ERR2612094
ERR2612095
ERR2612096
ERR2612097
ERR2612098
ERR2612099
ERR2612100
ERR2612101
ERR2612102
ERR2612103
ERR2612104
ERR2612105
ERR2612106
ERR2612107
ERR2612108
ERR2612109
ERR2612110
ERR2612111
ERR2612112
ERR2612113
ERR2091298
ERR2091299
ERR2091300

ERR2091301
ERR2091302
ERR2612038
ERR2612039
ERR2612040
ERR2612041
ERR2612042
ERR2612043
ERR2612044
ERR2612045
ERR2612046
ERR2612047
ERR2612048
ERR2612049
ERR2612050
ERR2612051
ERR2612052
ERR2612053
ERR2612058
ERR2612059
ERR2667128
ERR2667129
ERR2667130
ERR2667131
ERR2667132
ERR2667133
ERR2667134
ERR2667135
ERR2667136
ERR2667137
ERR2667138
ERR2667139
ERR2667140
ERR2667141
ERR2667142
ERR2667143
ERR2667144
ERR2667145
ERR2667146
ERR2667147
ERR2667148
ERR2667149
ERR2667150
ERR2667151
ERR2667075
ERR2667076
ERR2667077
ERR2667078
ERR2667079
ERR2667081

ERR2667082
ERR2667083
ERR2667084
ERR2667085
ERR2667086
ERR2667087
ERR2667088
ERR2667089
ERR2667090
ERR2667091
ERR2667092
ERR2667093
ERR2667094
ERR2667095
ERR2667096
ERR2667097
ERR2667098
ERR2667099
ERR2667100
ERR2667101
ERR2667102
ERR2667103
ERR2667104
ERR2667105
ERR2667106
ERR2667107
ERR2667108
ERR2667109
ERR2667110
ERR2667111
ERR2667112
ERR2667113
ERR2667114
ERR2667115
ERR2667116
ERR2667117
ERR2667118
ERR2667119
ERR2667120
ERR2667121
ERR2667122
ERR2667123
ERR2667124
ERR2667125
ERR2667126
ERR2667127
ERR311064
ERR311074
ERR311075
ERR311076

ERR311077
ERR311079
ERR311080
ERR311065
ERR311083
ERR311085
ERR311088
ERR311089
ERR311092
ERR311066
ERR311094
ERR311095
ERR311097
ERR311099
ERR311100
ERR311102
ERR311067
ERR311103
ERR311106
ERR311071
ERR316572
ERR316582
ERR316583
ERR316584
ERR316586
ERR316587
ERR316588
ERR316589
ERR316590
ERR316573
ERR316591
ERR316592
ERR316593
ERR316594
ERR316595
ERR316596
ERR316597
ERR316598
ERR316599
ERR316600
ERR316574
ERR316601
ERR316602
ERR316604
ERR316605
ERR316606
ERR316607
ERR316608
ERR316609
ERR316610

ERR316575
ERR316611
ERR316612
ERR316613
ERR316614
ERR316616
ERR316620
ERR316621
ERR316622
ERR316624
ERR316625
ERR316626
ERR316628
ERR316629
ERR316577
ERR316631
ERR316632
ERR316634
ERR316635
ERR316636
ERR316637
ERR316638
ERR316639
ERR316640
ERR316578
ERR316642
ERR316643
ERR316645
ERR316646
ERR316647
ERR316648
ERR316649
ERR316650
ERR316579
ERR316651
ERR316652
ERR316653
ERR316654
ERR316655
ERR316657
ERR316659
ERR316580
ERR316661
ERR316662
ERR316663
ERR316672
ERR316673
ERR316678
ERR316681
ERR316698

ERR316699
ERR316666
ERR316702
ERR316704
ERR316708
ERR316709
ERR316711
ERR316712
ERR316713
ERR316714
ERR316715
ERR316716
ERR316719
ERR316720
ERR316668
ERR316722
ERR316723
ERR316724
ERR316727
ERR316728
ERR316731
ERR316669
ERR316732
ERR316735
ERR316736
ERR316737
ERR316738
ERR316739
ERR316740
ERR316670
ERR316742
ERR316743
ERR316744
ERR316745
ERR316750
ERR316671
ERR316753
ERR316765
ERR316766
ERR316767
ERR316781
ERR316782
ERR316785
ERR316789
ERR316790
ERR316792
ERR316757
ERR316793
ERR316798
ERR316799

ERR316801
ERR316809
ERR316813
ERR316814
ERR316817
ERR316821
ERR316823
ERR316824
ERR316825
ERR316832
ERR316833
ERR316839
ERR316841
ERR316842
ERR316858
ERR316859
ERR316866
ERR316867
ERR316874
ERR316878
ERR316882
ERR316883
ERR316848
ERR316884
ERR316885
ERR316890
ERR316891
ERR316893
ERR316849
ERR316895
ERR316898
ERR316901
ERR316903
ERR316850
ERR316904
ERR316905
ERR316909
ERR316910
ERR316911
ERR316912
ERR316913
ERR316851
ERR316917
ERR316918
ERR316919
ERR316920
ERR316921
ERR316922
ERR316927
ERR316928

ERR316929
ERR316930
ERR316933
ERR316934
